# Amplified Light-Induced p*K*_a_ Modulation with Diarylethene Photoswitches

**DOI:** 10.1021/acs.joc.4c01606

**Published:** 2024-11-30

**Authors:** Marc Villabona, Arnau Marco, Rosa M. Sebastián, Gonzalo Guirado, Jordi Hernando

**Affiliations:** a Departament de Química, Universitat Autònoma de Barcelona, Edifici C/n, Campus UAB, 08193 Cerdanyola del Vallès, Spain; b Centro de Innovación en Química Avanzada (ORFEO−CINQA), Universitat Autònoma de Barcelona, 08193 Cerdanyola del Vallès, Spain

## Abstract

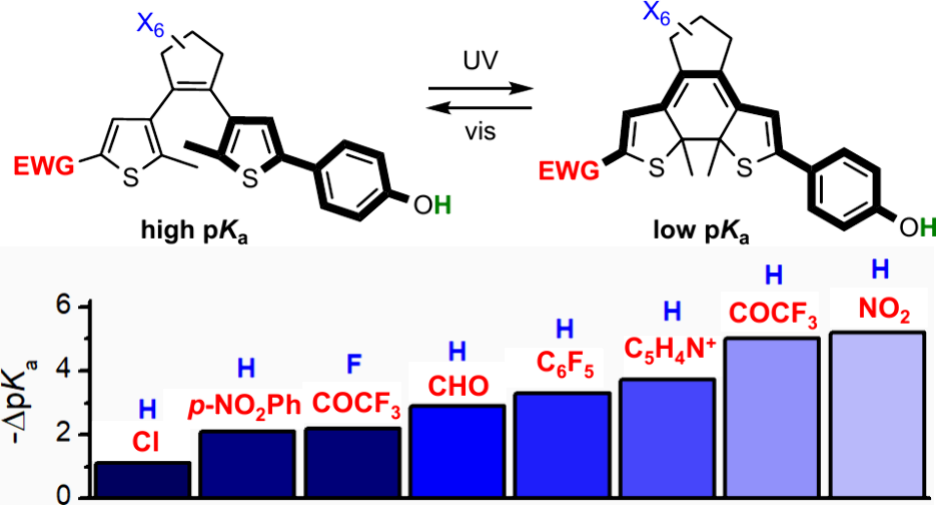

The reversible modulation of acidity using molecular photoswitches enables the remote control of a variety of (bio)chemical processes with light. Herein we investigated the structural features that allow amplifying photoinduced p*K*_a_ variation in phenol-diarylethene conjugates, which toggle between low- and high-acidity states by switching the conjugation between the ionizable moiety and electron-withdrawing groups upon photoisomerization. By tuning the structure of these conjugates, high p*K*_a_ modulation amplitudes were accomplished that surpass those previously reported.

## Introduction

By introducing molecular photoswitches into the structure of reagents, catalysts or ligands, the optical control of (bio)chemical processes can be accomplished in a reversible manner with high spatiotemporal precision.^1,2^ Among the different systems to which this strategy has been applied, the development of photoswitchable acids and bases has drawn particular interest,^3–6^ as the on-demand regulation of p*K*_a_ under light irradiation can find application in a variety of fields–e.g., polymer synthesis and processing,^4a,4d,5f,7^ catalysis,^3a,5h^ nanoparticle assembly,^8^ and CO_2_ capture·^5g,9^ To date, most of these light-sensitive compounds have been based on T-type photochromes such as azobenzenes and spiropyrans, with which large changes in acidity or basicity can be achieved upon photoisomerization.^3,4,6^ However, the photoinduced state of these systems is not thermally stable and spontaneously reverts back to the initial isomer in the dark. As a result, the modulation in acidity or basicity caused upon photoswitching is not permanent and it decays rapidly when illumination ceases, typically on the minute time scale.^3,4,6^

To overcome this constraint, the use of diarylethenes (DAEs) has been explored,^5^ which are P-type photochromes that toggle between two thermally stable open (**o**) and closed (**c**) isomers with light.^10^ Yet, moderate p*K*_a_ modulation has been attained experimentally with most of the DAE-based photoswitchable acid–base pairs reported so far. Thus, except for a thiazolone derivative described by Hecht et al. (Δp*K*_a_ = 2.8),^5e^ all the attempts to photoregulate the p*K*_a_ of more common acid groups such as carboxylic acids,^5d,5f^ phenols^5a,5b^ or ammonium ions^5c^ with DAEs have led to modest results (|Δp*K*_a_| < 1.7). Herein we present a systematic approach to overcome this limitation and develop DAE-based acids with enhanced light-induced p*K*_a_ variation. By amplifying p*K*_a_ photomodulation in these systems, larger and long-lived changes in pH could be induced under irradiation, which could be used for the manipulation of (bio)chemical processes with light.

**Scheme 1:**
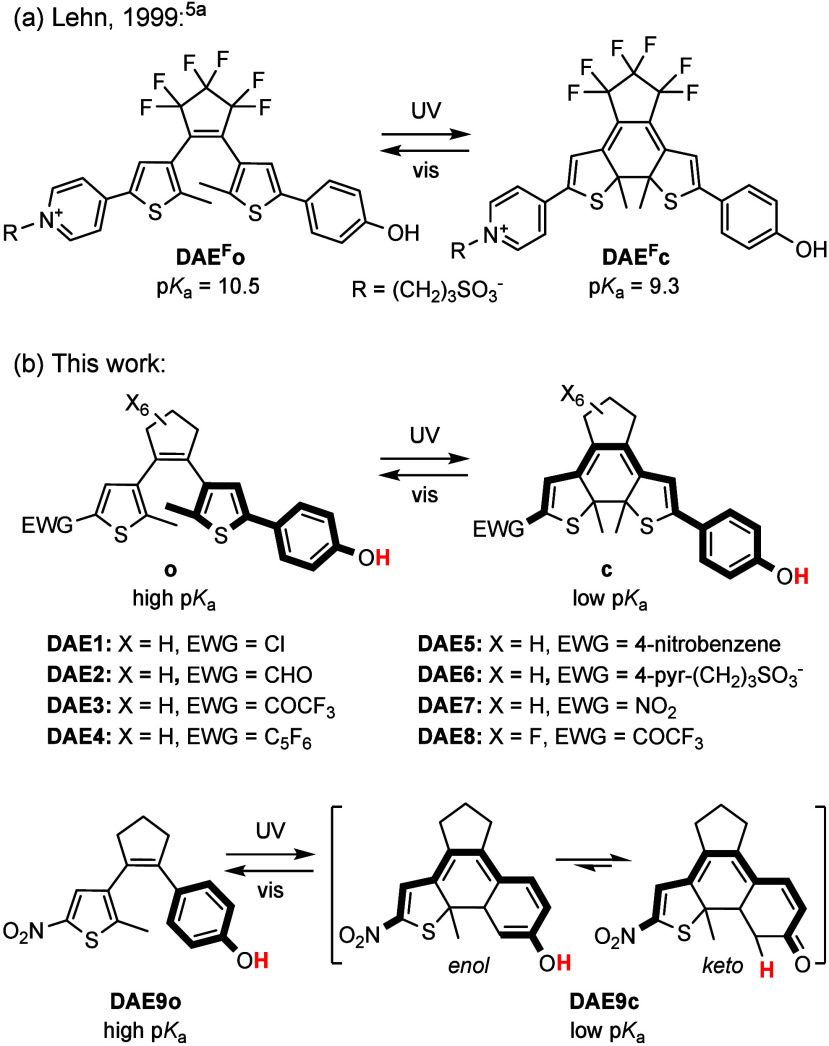
DAE-Based Phenols for Light-Modulated Acidity

Our strategy was inspired by the pioneering work from Lehn et al., who first utilized the change in conjugation occurring upon DAE photoisomerization to modulate the acidity of an appended group.^5a^ With this aim, they synthesized a DAE switch (**DAE**^**F**^) bearing two different functional units at the external thiophene positions: a phenol acid group and an electron-withdrawing 4-pyridinium moiety, which are electronically insulated in the open state of the system but become selectively conjugated in the closed isomer (Scheme 1a). As a result, the electronic communication created between these two groups upon photocyclization further stabilizes the phenolate conjugate base of **DAE**^**F**^ and reduces the p*K*_a_ of its closed form. However, the photomodulation in phenol acidity achieved with this design was relatively low (Δp*K*_a_ = −1.2), which we ascribe to two main factors. First, the electron-withdrawing strength of the 4-pyridinium substituent of **DAE**^**F**^ must be decreased by the attractive electrostatic interaction with its anionic *N*-propyl-1-sulfonate side chain. Therefore, a lower effect should be caused on the stability of the phenolate conjugate base of the closed isomer. Second, the perfluorinated cyclopentane central ring in **DAE**^**F**^ must exert stabilizing inductive effects on the conjugate bases of both the open and closed isomers and, consequently, dampen the p*K*_a_ variation between these two states.

In this work we addressed these two aspects to develop DAE-based photoswitchable phenols with amplified acidity modulation under irradiation. For this purpose, we synthesized a series of DAE-appended phenols where two distinct structural features were introduced relative to **DAE**^**F**^ (**DAE1–7**, Scheme 1b). First, they were functionalized with electron-withdrawing groups (EWG) of variable strength—i.e., low (Cl, 4-C_6_H_4_NO_2_, C_6_F_5_), medium (CHO, 4-pyridinium) and high (COCF_3_, NO_2_) electron-withdrawing power (Table S1). In this way, we could identify the better EWGs for maximizing Δp*K*_a_ between the two states of the switch. Second, a perhydrogenated cyclopentene central ring was introduced in **DAE1–7**, whose negligible inductive strength should neither affect the p*K*_a_ of these compounds nor attenuate the p*K*_a_ variation between their two isomers, in contrast to **DAE**^**F**^. To validate this hypothesis, we also prepared reference compound **DAE8**, which bears the same strong EWG as **DAE3** (COCF_3_) but contains the perfluorinated central ring of **DAE**^**F**^ (Scheme 1b). Finally, to further broaden our study on light-induced acidity modulation, compound **DAE9** was also synthesized, where the phenol group was directly integrated into the photoswitchable structure instead of appended to a thiophene ring (Scheme 1b). Although this should favor the electronic communication between the phenol moiety and the strong nitro EWG upon photocyclization, the keto–enol tautomerization expected to occur in the closed isomer of the system^11^ could reduce its effect on p*K*_a_ photomodulation.

## Results and Discussion

### Synthesis of DAE1–9

**Scheme 2:**
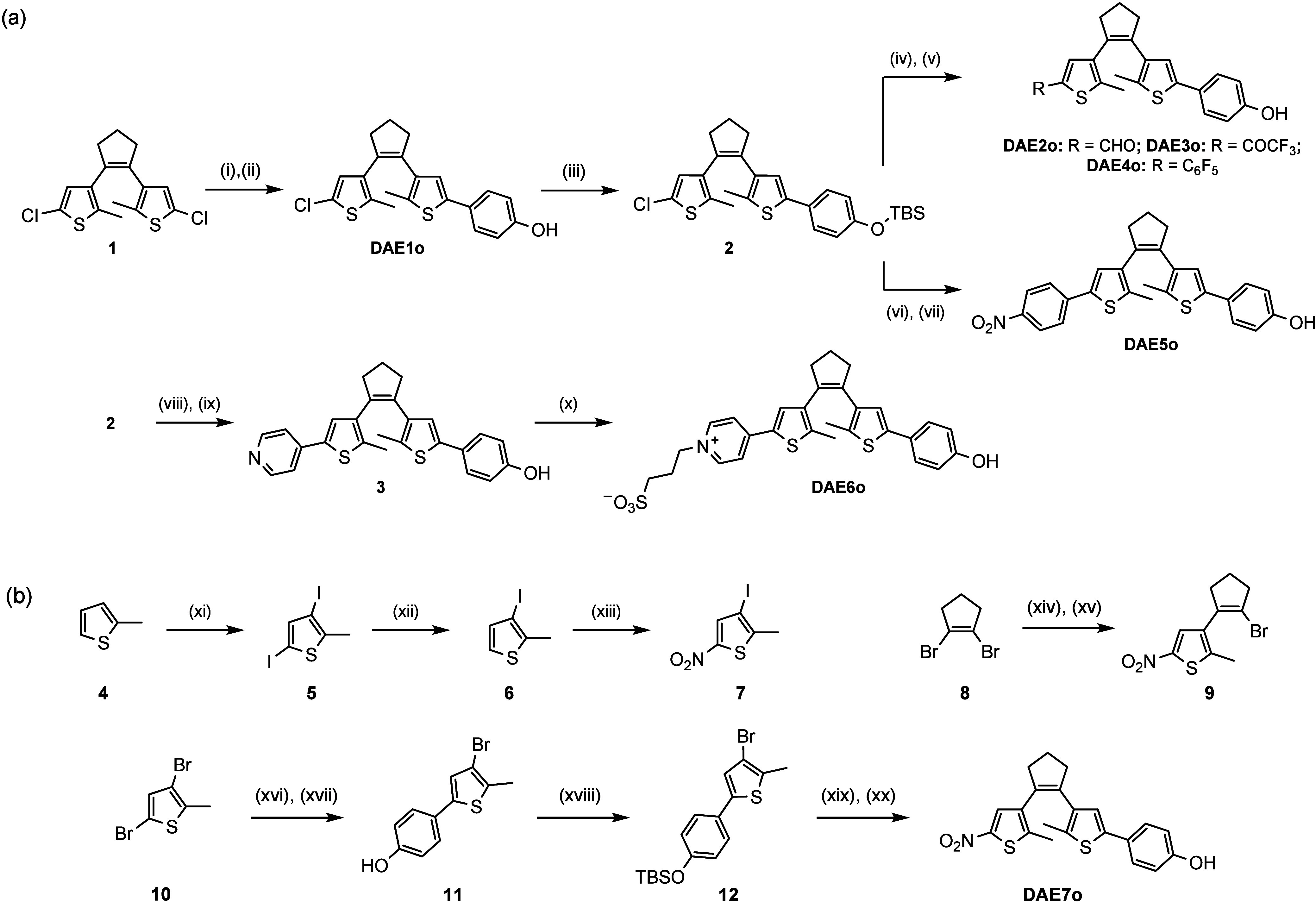
Synthesis of DAE1-7^*a*^ Reagents and conditions: (i) *t*BuLi, B(OBu)_3_, THF, −78 °C; (ii) 4-iodophenol, Pd(PPh_3_)_4_, Na_2_CO_3_, THF:H_2_O, reflux, (61% over two steps); (iii) TBSCl, imidazole, CH_2_Cl_2_, rt (85%); (iv) *t*BuLi, electrophile (DMF for **DAE2o**, CF_3_COOEt for **DAE3o** or C_6_F_6_ for **DAE4o**), THF, −78 °C; (v) TBAF, AcOH, CHCl_3_, rt (90% for **DAE2o**, 35% for **DAE3o** and 74% for **DAE4o**); (vi) as in (i); (vii) 1-iodo-4-nitrobenzene, Pd(PPh_3_)_4_, Na_2_CO_3_, THF:H_2_O, reflux, (46% over two steps); (viii) as in (i); (ix) 4-bromopyridinium hydrochloride, Pd(PPh_3_)_4_, Na_2_CO_3_, THF:H_2_O, reflux, (54% over two steps); (x) 1,3-propane sultone, acetonitrile, reflux (30%); (xi) I_2_, NaIO_4_, MeOH, H_2_SO_4_, reflux (99%); (xii) *n*BuLi, MeOH, THF, −78 °C (81%); (xiii) HNO_3_, acetic anhydride, 0 °C (46%); (xiv) *n*BuLi, B(OBu)_3_, THF, −78 °C; (xv) **7**, Pd(PPh_3_)_4_, Na_2_CO_3_, THF:H_2_O, reflux (81% over two steps); (xvi) as in (xiv); (xvii) as in (ii), (67% over two steps); (xviii) as in (iii) (80%); (xix) as in (xiv); (xx) **9**, Pd(PPh_3_)_4_, Na_2_CO_3_, THF:H_2_O, reflux (52% over two steps). Abreviatures: TBSCl: *tert*-butyldimethylchlorosilane; TBAF: tetrabutylammonium fluoride.

For the preparation of **DAE1–9** in their open state, different synthetic strategies had to be utilized (Scheme 2 and Schemes S1–2). Compounds **DAE1o–6o** were synthesized from 1,2-bis(2-chloro-5-methylthien-4-yl)cyclopentene (**1**), which is a common intermediate for the preparation of DAEs via lithiation-initiated processes (Scheme 2a).^12^ First, **DAE1o** was obtained by a two-step process consisting of a *t*-butyllithium-mediated borylation and a Suzuki coupling with 4-iodophenol.^13^ The synthesis of **DAE2o–6o** proceeded through the protection of the phenol group of **DAE1o** as a silyl ether (**2**),^14^ and then the desired EWGs were introduced by two different methods: (1) **DAE2o–4o** were prepared through *t*-butyllithium-mediated reaction with the corresponding electrophile^12a^–i.e., DMF for **DAE2o**, ethyl trifluoroacetate for **DAE3o**, and perfluorobenzene for **DAE4o**; (2) **DAE5o** and **DAE6o** were obtained through successive *t*-butyllithium-mediated borylation and Suzuki coupling with 1-iodo-4-nitrobenzene (**DAE5o**) and 4-bromopyridine, the latter of which produced intermediate **3** that was finally *N*-alkylated to obtain **DAE6o**.^5a^

Because nitro groups are not compatible with organolithium reagents, a more complex synthetic strategy had to be devised for the synthesis of **DAE7o** (Scheme 2b). It consisted in preparing the two substituted thiophene rings **7** and **12** separately, and then tethering them to 1,2-dibromocyclopentene (**8**). Due to regioselectivity issues, a doble iodination process was required to iodinate position 3 of 2-methylthiophene (**4**),^15^ and consequently, this reaction had to be followed by *n*-butyllithium mediated removal of the iodide in position 5 of intermediate **5** to produce **6**.^16^ Next, nitration of **6** was conducted to obtain thiophene **7** using acetic anhydride as a solvent.^17^ Thiophene **12** was prepared from 3,5-dibromo-2-methylthiophene (**10**) following *n*-butyllithium-mediated borylation, Suzuki coupling (**11**), and silyl ether protection. Once synthesized **7** and **12**, **DAE7o** was prepared by successively coupling these thiophene derivatives with **8**. In both cases, this was accomplished through Suzuki coupling, previously installing the boronic ester in the fragment that did not contain the nitro group through lithiation and borylation.

As for compounds **DAE8o–9o**, they were synthesized through other procedures. For **DAE8o**, a common method for the preparation of asymmetric perfluorinated DAEs was used (Scheme S1).^12a^ First, thiophene derivatives **13** and **12** were sequentially introduced to perfluorocyclopentene via lithiation to obtain intermediate **15**. Then, **DAE8o** was furnished by further lithiation of **15** and reaction with ethyl trifluoroacetate. In the case of **DAE9o**, its synthesis started with the protection of 4-bromophenol (**Br-PhOH**) as a silyl ether (**16**). Then it was coupled to previously prepared intermediate **9** via *n*-butyllithium-mediated borylation and Suzuki coupling, and **DAE9o** was finally obtained after removal of the silyl ether group (Scheme S2).

### Photochemical Characterization of DAE1–9

**Figure 1:**
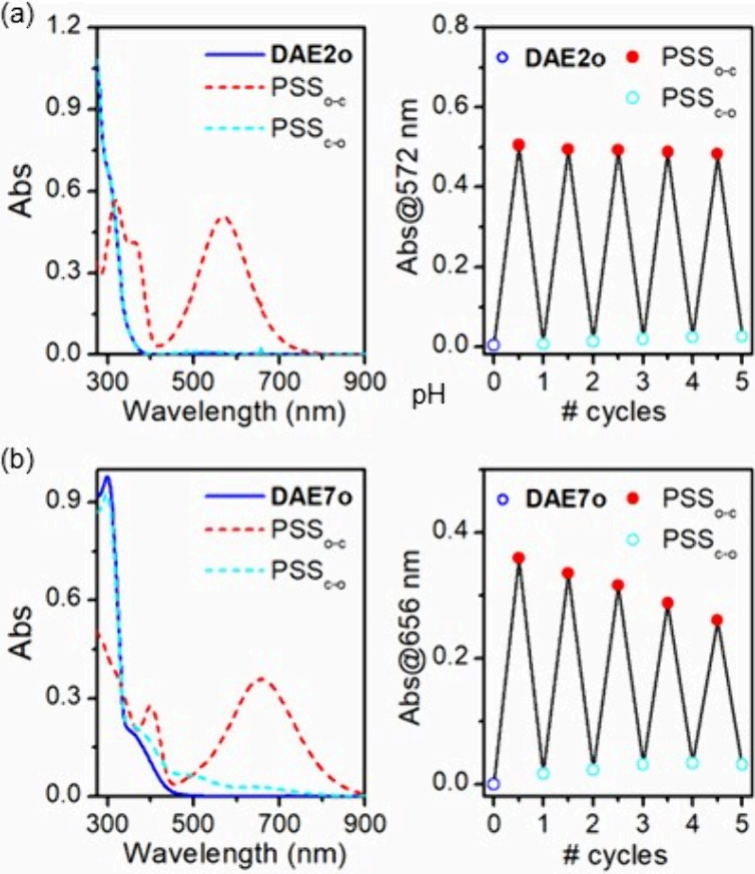
Photochemical data for (a) **DAE2** and (b) **DAE7** in acetonitrile solution (*c* = 5.0 × 10^–5^ M). (left) Absorption spectra of the initial open isomer, PSS_o-c_ (λ_exc_ = 312 nm), and PSS_c-o_ (λ_exc_ = 650 nm). (right) Variation of the absorbance at the spectral maximum of the closed isomer upon repetitive photoswitching with λ_exc_ = 312 and 650 nm.

**Figure 2:**
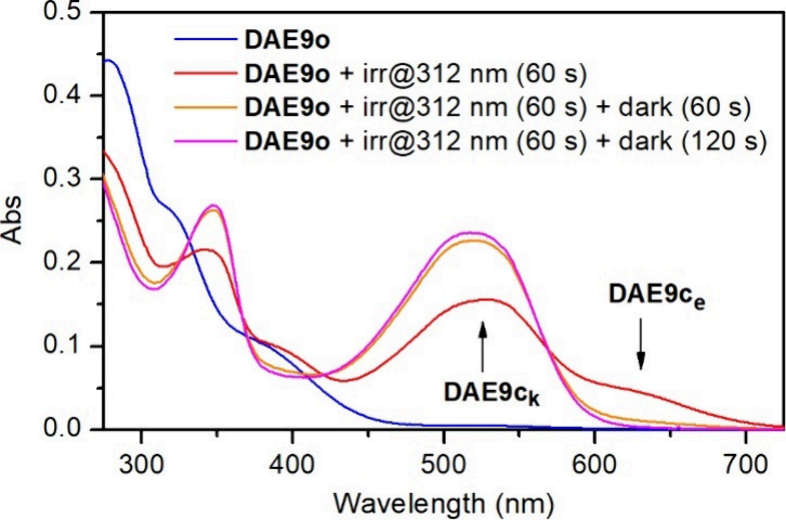
Variation of the absorption spectrum of **DAE9** (*c* = 3.4 × 10^–5^ M) upon photocyclization in dichloromethane at room temperature: pure open-state **DAE9o**, after irradiation at λ_exc_ = 312 nm for 60 s, after 60 s in the dark postirradiation, and after 120 s in the dark postirradiation.

All the phenols **DAE1–9** prepared exhibited the characteristic photoswitchable behavior of diarylethenes in solution.^10^ As illustrated in Figure 1 for **DAE2** and **DAE7**, their colorless open isomer underwent photocyclization to the colored closed state upon irradiation with UV or violet light (312, 365, or 405 nm), whereas this process could be reverted by illumination with visible radiation (532 or 650 nm) (see also Table 1 and Figure S1). In the case of **DAE9**, an additional feature was observed: the absorption spectrum measured upon UV-induced photocyclization evolved in time in the dark (Figure 2). In particular, a transient closed-state species absorbing at λ_abs_ ∼ 620 nm was initially formed under UV irradiation, which rapidly transformed into a new colored form with λ_abs_ ∼ 520 nm that could be photoisomerized back to the open state. As previously reported for similar compounds,^11^ we ascribed this behavior to the keto–enol tautomerization of the closed isomer of **DAE9** (see Scheme 1b). Thus, the photocyclization of **DAE9o** first produced the enol tautomer of **DAE9c** (**DAE9c**_**e**_, λ_abs_ ∼ 620 nm), which then evolved to the more stable closed-state keto isomer (**DAE9c**_**k**_, λ_abs_ ∼ 520 nm). Importantly, such tautomerization process was found to occur more rapidly (∼2 min) than for other analog DAE switches already described (∼20 min),^11^ which we attributed to the electron-withdrawing effect of the nitro substituent of **DAE9c**.

**Table 1:** Photochemical Properties and p*K*_a_ of DAE1-9^a^

	λ_*abs*_^o^ [nm] (ε [M^–1^ cm^–1^])^b^	λ_*abs*_^c^ [nm] (ε [M^–1^ cm^–1^])^c^	PSS_o-c_ [%]^d^	PSS_c-o_ [%]^e^	Φ_o-c_^f^	Φ_c-o_^f^	p*K*_a_^o^^g^	p*K*_a_^c^^g^
**DAE1**	281 (17.5 × 10^3^)	484 (11.8 × 10^3^)	24:76	100:0	0.20	0.020	26.2 ± 0.4	25.1 ± 0.2
**DAE2**	272 (21.7 × 10^3^)	572 (10.4 × 10^3^)	3:97	100:0	0.25	0.021	26.2 ± 0.2	23.0 ± 0.2
**DAE3**	287 (25.5 × 10^3^)	641 (10.4 × 10^3^)	89:11	100:0	0.029	0.049	25.6 ± 0.1	20.6 ± 0.1
**DAE4**	276 (30.1 × 10^3^)	518 (18.6 × 10^3^)	28:72	100:0	0.15	0.090	25.7 ± 0.1	22.4 ± 0.1
**DAE5**	266 (14.6 × 10^3^)	595 (11.7 × 10^3^)	2:98	100:0	0.17	4.4 × 10^–3^	25.9 ± 0.1	23.8 ± 0.2
**DAE6**	282 (17.0 × 10^3^)	664 (13.7 × 10^3^)	67:33	100:0	1.5 × 10^–3^	0.022	25.7 ± 0.3	22.0 ± 0.4
**DAE7**	298 (19.6 × 10^3^)	656 (8.7 × 10^3^)	17:83	100:0	0.076	0.018	26.0 ± 0.7	20.8 ± 0.3
**DAE8**	279 (13.4 × 10^3^)	662 (10.4 × 10^3^)	50:50	100:0	0.16	0.039	22.6 ± 0.5	20.4 ± 0.1
**DAE9**	280 (13.7 × 10^3^)	511 (11.4 × 10^3^)	6:94^h^	100:0	0.66	0.013	26.6 ± 0.1	24.9 ± 0.2

a In acetonitrile, except for the photochemical properties of **DAE9** that were measured in cyclohexane.

b Wavelength and molar absorptivity coefficient of the absorption band maximum of the open isomer.

c Wavelength and molar absorptivity coefficient of the absorption band maximum of the closed isomer.

d Open:closed concentration ratio in the PSS_o-c_ obtained under irradiation at 312 nm (**DAE1–2**, **DAE4** and **DAE7**), 355 nm (**DAE5–6**, and **DAE8–9**) or 405 nm (**DAE3**).

e Open:closed concentration ratio in the PSS_c-o_ obtained under irradiation at 532 nm (**DAE1**, **DAE4** and **DAE9**) or 650 nm (**DAE2–3**, **DAE5–9**).

f Photocyclization (Φ_o-c_) and photocycloreversion (Φ_c-o_) quantum yields, which were measured at the same irradiation wavelengths as the PSS compositions.

g Acidity constants of the open (p) and closed (p) isomers referred to the acetonitrile scale.

h The open:closed concentration ratio in the PSS_o-c_ decreased to 34:66 in CH_2_Cl_2_, while no photocyclization was observed in acetonitrile.

**Figure 3:**
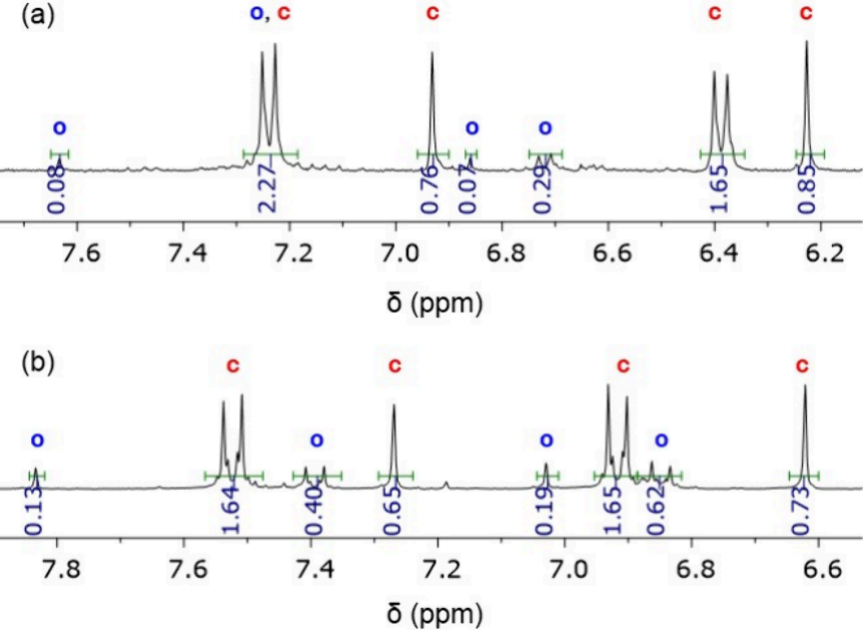
(a) Low field region of the ^1^H NMR spectrum (250 MHz, CD_3_CN) of the PSS_o-c_ of **DAE2** obtained at λ_exc_ = 312 nm. (b) Low field region of the ^1^H NMR spectrum (250 MHz, acetone-*d*_6_) of the PSS_o-c_ of **DAE7** obtained at λ_exc_ = 355 nm, which was used together with UV–vis absorption data to determine the PSS_o-c_ composition in acetonitrile (see the Supporting Information for further details). In both cases, the ^1^H NMR resonances of the open and closed isomers are denoted as **o** and **c**, respectively.

Whereas the light-induced ring-opening process of **DAE1–9** was observed to proceed quantitatively in all cases, variable efficiencies were measured for their photocyclization reaction in acetonitrile solution by UV–vis and NMR spectroscopies (Table 1 and Figure 3). For **DAE2** and **DAE5**, high ring-closing conversions (>95%) were determined in acetonitrile, which allowed repetitive photoswitching with minor photodegradation effects (Figure 1a and Figure 3a). Lower though still large photocyclization efficacies (>50%) were registered for **DAE1**, **DAE4**, **DAE7** and **DAE8** in acetonitrile, which exhibited less fatigue resistance to successive photoswitching (Figure 1b and Figure 3b). Finally, low ring-closing conversions (<35%) were measured for **DAE3** and **DAE6** in acetonitrile, while no photocyclization could be registered for **DAE9** in this solvent. These differences in photocyclization efficiency for **DAE1**–**DAE9** can be mainly rationalized based on their ring-closing (Φ_o-c_) and ring-opening (Φ_c-o_) quantum yields. For DAE with high Φ_o-c_/Φ_c-o_ ratio, photocyclization must be largely favored over photocycloreversion under illumination with UV light, which is absorbed by both isomers. As a result, they should exhibit UV-induced photostationary states with a large closed-state content.^10^ This is the case herein of **DAE2** and **DAE5** (Φ_o-c_/Φ_c-o_ > 10) and, in a lower extent, of **DAE1**, **DAE4**, **DAE7** and **DAE8** (Φ_o-c_/Φ_c-o_ ∼ 1.5–10), all of which showed rather high ring-closing conversions. In contrast, the Φ_o-c_/Φ_c-o_ ratio determined for **DAE3** and **DAE6** were very low (Φ_o-c_/Φ_c-o_ < 0.6), which agrees with their poor photocyclization efficiencies.

The variation of the Φ_o-c_/Φ_c-o_ ratio and, therefore, of the ring-closing photoconversion for **DAE1–9** can be fairly related to their Φ_o-c_ values. Thus, while small ring-opening quantum yields were measured in all the cases (Φ_c-o_ < 0.05), their Φ_o-c_ values varied broadly in acetonitrile, and they were especially low (or even null) for **DAE3**, **DAE6** and **DAE9** (Φ_o-c_ < 0.03). We ascribe this behavior to the electronic asymmetry of these compounds, where both electron-rich–i.e., phenol– and electron-poor–i.e., EWG-substituted thiophene–aryl groups– coexist. As previously reported, such push–pull character can hamper DAE photocyclization and lead to low Φ_o-c_ values,^18^ as it is herein the case of **DAE3**, **DAE6** and **DAE9**. Two main factors could account for this situation. First, the presence of electron-rich and -poor aryl groups in DAE can favor the unreactive parallel conformation of the open isomer through π–π stacking.^18^ Second, electron asymmetry can promote internal charge transfer processes after photoexcitation that compete with photoisomerization.^18^ The latter is especially favored in polar solvents such as acetonitrile, which we validated by additional experimental data. On the one hand, we found Φ_o-c_ to increase up to 0.20 for **DAE3** in hexane, which led to higher ring-closing conversion (48%). On the other hand, whereas negligible photoisomerization was observed for **DAE9** in acetonitrile, it could be photoswitched in dichloromethane and cyclohexane with moderate-to-high efficacy (66% and 94%, respectively, Table 1).

### Light-Induced p*K*_a_ Modulation for DAE1–9

**Figure 4:**
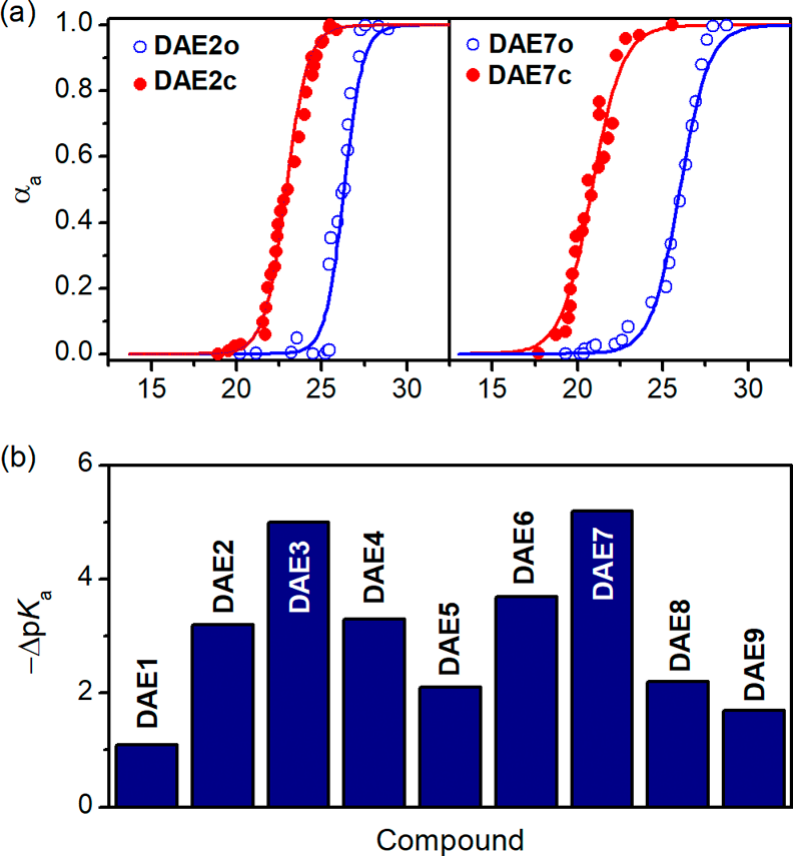
(a) Titration curves of the open and closed isomers of **DAE2** and **DAE7** with TBAOH in acetonitrile, where the degree of deprotonation (α_a_) determined spectrophotometrically is plotted against pH. (b) p*K*_a_ modulation (−Δp*K*_a_ = p – p) measured for **DAE1–9** in acetonitrile.

The acidity constants of the open and closed isomers of **DAE1–9** could be obtained by spectrophotometric titration with a stock solution of tetrabutylammonium hydroxide (TBAOH), as a pronounced bathochromic shift of their absorption bands was observed in all the cases upon deprotonation (Table 1, Figure 4a and Figure S2; see the Supporting Information for further details). As **DAE1–9** are poorly soluble in aqueous media, these experiments were conducted in acetonitrile. As a result, p*K*_a_ values were measured relative to the acetonitrile solvent (_*s*_^*s*^p*K*_a_), whose _*s*_^*s*^pH scale spans from 0 to 34.^19^

For all the open states of the compounds bearing a perhydrogenated central cyclopentene ring—i.e., **DAE1–7** and **DAE9**—similar p*K*_a_ values (p) were found that range from 25.6 to 26.6 and resemble that reported for phenol in acetonitrile (p*K*_a_ = 26.6^20^). Therefore, this result suggests that negligible electronic communication exists between their phenol and EWG substituents. On the other hand, a significantly lower p constant was determined for **DAE8** (p = 22.6). This result proves that its perfluorinated cyclopentene group imparts strong inductive electron-withdrawing effects on the phenol moiety of the open state, thus substantially increasing acidity.

Upon photocyclization, lower p*K*_a_ values were measured for the closed isomer of **DAE1–9** (p), which reveals that light-induced acidity modulation occurs in all cases (Figure 4b). This behavior can be ascribed to two main factors that should aid stabilizing the negative charge generated upon deprotonation and, therefore, rising acidity. These factors are the larger conjugation pathway of the photocyclized form, and the electronic communication established with the lateral EWG upon ring-closing. Indeed, a clear relationship was observed between p and the strength of the external EWG for most of our DAE phenol conjugates. As anticipated in our molecular design, lower acidities were registered for closed isomers bearing the less electron-withdrawing substituents such as Cl (p = 25.1). In contrast, a much larger decrement in p was measured for the compounds with the strongest EWGs such as **DAE3** (EWG = COCF_3_, p = 20.6) and **DAE7** (EWG = NO_2_, p = 20.8), which turned to be as acid as 4-nitrophenol (p*K*_a_ = 20.7 in acetonitrile^20^). The only exception to this behavior is **DAE9**, for which the highest p constant was measured despite bearing a strong nitro EWG (p = 24.9). This result can be attributed to the ketone structure of **DAE9c** after tautomerization, whose deprotonation should take place at the α-carbonylic position that is less acidic than the ionizable phenol group found in **DAE1c–8c** (Scheme S3). Consequently, this feature should counterbalance the electronic stabilizing effect caused by its nitro group upon photocyclization, thus leading to low acidity modulation (Δp*K*_a_ = −1.7). In view of this, separation of the acid phenol moiety from the photoswitching unit is preferable to maximize light-induced p*K*_a_ variation, as we did in **DAE1–8**.

However, when considering the acidity modulation obtained for **DAE1–8**, another case was found not to follow the correlation between Δp*K*_a_ and EWG strength (Figure S3). Thus, though the lowest p constant was determined for **DAE8** with a strong trifluoroacetyl EWG (p = 20.4), the Δp*K*_a_ accomplished was lower than expected (Δp*K*_a_ = −2.2). This result is due to the introduction of a fluorinated central ring in this compound, an inductive EWG that affects the phenol moiety of both its open and closed isomers. As a consequence, it increases the acidity of both states, thus strongly attenuating the light-induced p*K*_a_ variation achieved. Instead, when installing an innocent perhydrogenated cyclopentene in the DAE core, much higher Δp*K*_a_ values were reached with the same (or similar) lateral EWGs: Δp*K*_a_ = −5.0 and −5.2 for **DAE3** (EWG = COCF_3_) and **DAE7** (EWG = NO_2_), respectively (Figure 4b).

**Figure 5:**
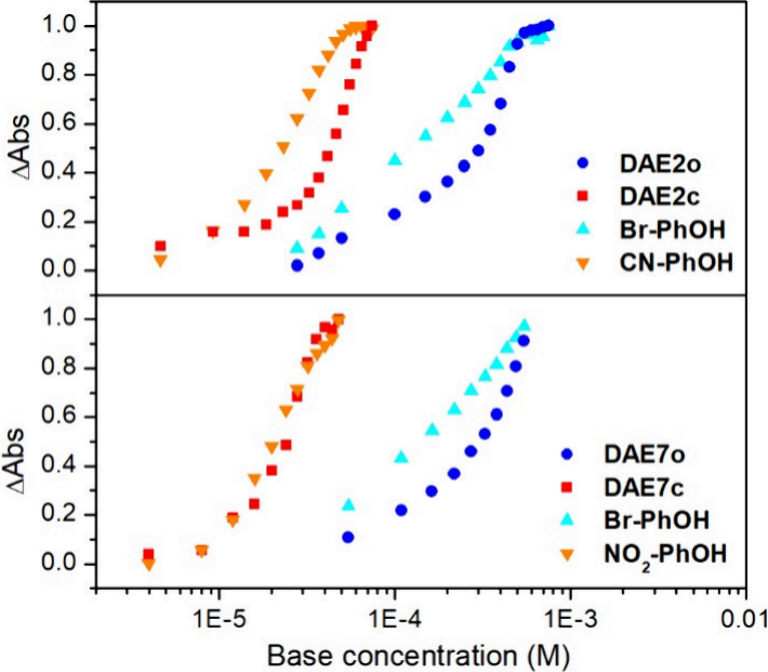
(a) Increment of the absorbance of the deprotonated phenolate form measured upon titration with TBAOH of: a mixture of **DAE2o** (λ_abs_ = 360 nm) and **Br-PhOH** (λ_abs_ = 320 nm); a mixture of **DAE2c** (λ_abs_ = 700 nm) and **CN-PhOH** (λ_abs_ = 295 nm). (b) Increment of the absorbance of the deprotonated phenolate form measured upon titration with TBAOH of: a mixture of **DAE7o** (λ_abs_ = 360 nm) and **Br-PhOH** (λ_abs_ = 320 nm); a mixture of **DAE7c** (λ_abs_ = 800 nm) and **NO**_**2**_**–PhOH** (λ_abs_ = 305 nm). In all the cases,  = 1.5 × 10^–5^ M and  = 3 × 10^–5^ M.

To corroborate the ample light-induced modulation achieved with these compounds, additional experiments were conducted on **DAE2** and **DAE7** as representative examples of DAE-appended phenols with medium and high Δp*K*_a_. These experiments consisted in the titration of binary mixtures of the open or closed isomers of these compounds with a phenol of similar reported p*K*_a_ value in acetonitrile: 4-bromophenol (**Br-PhOH**, p*K*_a_ = 25.5^20^) for both **DAE2o** and **DAE7o**, 4-cyanophenol (**CN-PhOH**, p*K*_a_ = 22.7^20^) for **DAE2c**, and 4-nitrophenol (**NO**_**2**_-PhOH, p*K*_a_ = 20.7^20^) for **DAE7c** (Figure S4). By spectrophotometrically monitoring the deprotonation process of these mixtures of phenols, we could then correlate the p*K*_a_ values of the compounds developed herein with those of the phenols already reported (Figure S5). In this way, we could first demonstrate that **DAE2o** and **DAE7o** have slightly higher p*K*_a_ than 4-bromophenol (p*K*_a_ > 25.5), as they required larger base concentrations to be deprotonated (Figure 5). Therefore, this result gives further proof of the high p*K*_a_ values measured for **DAE2o** (p = 26.2) and **DAE7o** (p = 26.0), as well as for most of the open-state DAE-based phenols described in this work. On the other hand, we observed that **DAE2c** was deprotonated with a slightly larger base amount than 4-cyanophenol, which corroborates that its p*K*_a_ constant must be higher than 22.7, as experimentally obtained (p = 23.0) (Figure 5a). Finally, the deprotonation of **DAE7c** and 4-nitrophenol occurred almost simultaneously, which proved the low p*K*_a_ value measured for **DAE7c** (p = 20.8) (Figure 5b). Overall, these data show the capacity to photomodulate phenol acidity with DAE photochromes and, more importantly, confirm the large Δp*K*_a_ achieved for some of the compounds prepared such as **DAE7**.

Indeed, based on our Δp*K*_a_ measurements, **DAE3** and **DAE7** are the best candidates to optically control phenol acidity with DAE photoswitches (Figure 4b). Interestingly, the performance of these compounds as photoswitchable acids surpasses the performance of most DAE-based systems reported to date (Table S2). In acetonitrile, experimental Δp*K*_a_ values disclosed so far lie within −1.7–0.4,^5c,5^ and are therefore about 3-fold lower than those achieved herein. Actually, our results approach the acidity modulation predicted computationally for DAEs bearing central *N*-heterocyclic imines as acid–base groups (Δp*K*_a_ = −6.1 to −8.7), which is the best case described in the literature.^5g^ To further compare the behavior of **DAE3** and **DAE7** with DAE-based photoswitchable acids investigated in aqueous media, we estimated the Δp*K*_a_ values of our compounds in water using reported data on phenol acidity: Δp*K*_a_ = −2.9 and −3.0 for **DAE3** and **DAE7**, respectively (Figure S6 and Table S2; see the Supporting Information for further details). These values also top those previously described for aqueous solutions of photoswichable phenols (Δp*K*_a_ = −0.4 to −1.2)^5a,5b^ and thiazolones (Δp*K*_a_ = 2.8).^5e^ Of special interest is the comparison with the acidity modulation of precursor **DAE**_**F**_ (Δp*K*_a_ = −1.2),^5a^ which is about 2.5-fold lower than the best values obtained herein. Therefore, this result validates our molecular approach toward amplified photoswitchable acidity in DAE-phenol conjugates.

## Conclusions

In this work we reported a systematic study of the structural parameters that allow enhancing light-induced p*K*_a_ modulation in acid groups appended to diarylethene photoswitches. By preparing a series of phenol-DAE conjugates, three main motifs were identified that lead to optimal acidity variation between their ring-open and ring-closed states: (1) the separation of the acid phenol moiety from the photoswitching unit, and the introduction of (2) a perhydrogenated cyclopentene ring in the DAE core, and (3) stronger lateral EWGs. In this way, p*K*_a_ modulation values as high as Δp*K*_a_ = −5.2 in acetonitrile (Δp*K*_a_ ∼ −3.0 in aqueous media) could be obtained, which surpass those previously determined experimentally for other DAE-based photoswitchable acids and bases.

## Experimental Section

### Materials and Methods

All commercially available reagents were used as received. Anhydrous THF and CH_2_Cl_2_ were used after column drying in a solvent dispenser from Innovative technology (PureSolv-MD-2) while the rest of organic solvents were dried with molecular sieves, 3 A beads, 4–8 mesh from Sigma-Aldrich.

NMR spectra were recorded on Bruker DPX250 (250 MHz for ^1^H), DPX360 (360 MHz for ^1^H), AvanceIII 400NB (400 MHz for ^1^H), Bruker Avance NEO 300 MHz (300 MHz for ^1^H) and 600 Ascend LH (600 MHz for ^1^H) spectrometers. The δ-scale was normalized relative to the residual solvent signal for ^1^H NMR and ^13^C{^1^H} NMR (CDCl_3_ (7.26 ppm for ^1^H; 77.2 ppm for ^13^C), CD_3_CN (1.94 ppm for ^1^H; 118.3 and 1.3 ppm for ^13^C), acetone-*d*_6_ (2.05 ppm for 1H; 206.2 and 29.8 ppm for ^13^C), DMSO-*d*_6_ (2.50 ppm for ^1^H; 39.5 ppm for ^13^C), and toluene-*d*_8_ (7.09, 7.01, 6.97, and 2.08 ppm for ^1^H; 137.5, 128.9, 128.0, 125.1, and 20.4 ppm for ^13^C)), and relative to CFCl_3_ for ^19^F NMR (0.00 ppm in all the solvents). The abbreviations used to describe signal multiplicities are s (singlet), bs (broad singlet), d (doublet), t (triplet), q (quadruplet), p (pentuplet), h (hexaplet), sept (septuplet), dd (double doublet), dt (double triplet), dq (double quadruplet), tt (triple triplet) and m (multiplet). NMR signals for **DAE1–9** were assigned with the help of COSY, HSQC and HMBC measurements.

IR-ATR spectra were recorded on a Bruker Tensor 27 Golden Gate spectrometer with a diamond tip. Infrared peaks are reported in cm^–1^. Mass spectra were recorded on a Bruker microTOFQ spectrometer using ESIMS and on a Bruker Esquire 3000+ spectrometer using ESI or APCI. UV–vis absorption spectra were recorded on an Agilent HP 8453 spectrophotometer. Samples were measured in Hellma Analytics quartz high precision cells with a path length of 10 mm at ambient temperature.

pH measurements upon base addition to acetonitrile solutions were performed at room temperature with a Crison 5028 pH electrode in a Crison BASIC 20+ potentiometer and a Hamilton MiniTrode 238100 pH electrode in a VioLab pH 50 potentiometer. pH values are given relative to the acetonitrile solvent ( scale).^19^ To calibrate the electrode system we used reference buffers in acetonitrile (pyridine-pyridinium bromide, phenol-sodium phenolate, and 4-nitrophenol-sodium 4-nitrophenolate), whose  can be derived from the Henderson–Hasselbach equation using the p*K*_a_ values in acetonitrile reported for these systems.^20^

### Synthesis of DAE1–9

Synthesis of starting materials **1**,^12b^**8**,^21^**10**^22^ and **13**(23) was conducted according to the literature. Procedures for the synthesis of already reported intermediates **5**,^15^**6**,^24^**14**^15^ and **16**(25) are provided in the Supporting Information.

#### 4-(4-(2-(5-Chloro-2-methylthiophen-3-yl)cyclopent-1-en-1-yl)-5-methylthiophen-2-yl)phenol (**DAE1o**)

A solution of 1.824 g of **1** (5.54 mmol) in 30 mL of anhydrous THF was cooled down to −78 °C. Then 3.8 mL of a 1.7 M solution of *tert-*butyllithium in pentane (6.40 mmol) were added cautiously. After stirring for 30 min at −78 °C, the mixture was quenched with 1.8 mL of tributyl borate (6.70 mmol) and then it was stirred for 30 min more until it reached room temperature. Later the reaction mixture was added onto a degassed solution of 1.221 g of 4-iodophenol (7.06 mmol) and 0.248 mg of Pd(PPh_3_)_4_ (0.22 mmol) in 30 mL of THF and 30 mL of a 2 M Na_2_CO_3_ aqueous solution. The two-phase system was heated with a metallic heat-on block under reflux for 2 h. Afterward, the crude was cooled down to room temperature and 50 mL of Et_2_O were added. After separating the organic layer, the aqueous phase was extracted with additional 50 mL of Et_2_O. The combined organic layers were dried with anhydrous Na_2_SO_4_ and, after filtration and removal of the solvent under vacuum, a red oil was obtained. The product was then purified through flash column chromatography (silica gel, EtOAc/hexane 1:9) to yield 1.309 g of compound **DAE1o** (3.38 mmol; 61% yield). The ^1^H NMR spectrum of this compound matched previously reported data.^13 1^H NMR (600 MHz, CDCl_3_): δ 7.37 (d, *J* = 8.6 Hz, 2H, H-3 and H-5), 6.86 (s, 1H, H-8), 6.81 (d, *J* = 8.8 Hz, 2H, H-2 and H-6), 6.62 (s, 1H, H-18), 4.91 (s, 1H, OH), 2.80 (t, *J* = 7.4 Hz, 2H, H-14), 2.74 (t, *J* = 7.5 Hz, 2H, H-16), 2.04 (p, *J* = 7.5 Hz, 2H, H-15), 1.98 (s, 3H, H-11), 1.89 (s, 3H, H-21). ^13^C{^1^H} NMR (151 MHz, CDCl_3_): δ 155.0 (C-1), 139.8 (C-7), 136.3 (C-10), 135.5 (C-12 or C-13), 135.3 (C-20), 133.7 (C-12 or C-13), 133.6 (C-9), 133.4 (C-19), 127.7 (C-4), 127.0 (C-18), 126.9 (C-3 and C-5), 125.1 (C-17), 122.9 (C-8), 115.8 (C-2 and C-6), 38.6 (C-14), 38.5 (C-16), 23.1 (C-15), 14.5 (C-11), 14.3 (C-21).

#### 1-(2-Methyl-5-(4-tert-butyldimethylsilyl)oxy)phenyl)thien-3-yl)-2-(5-chloro-2-methylthien-3-yl) Cyclopentene (**2**)

0.227 g of **DAE1o** (0.58 mmol), 0.101 g of TBSCl (0.67 mmol) and 0.071 g of imidazole (1.03 mmol) were stirred in 10 mL of anhydrous CH_2_Cl_2_ at room temperature overnight. Then the crude was washed with 10 mL of water. The organic phase was dried over Na_2_SO_4_, filtered and the solvent removed under vacuum. Compound **2** was then purified via flash column chromatography (silica gel, hexane) to obtain 0.249 g (0.50 mmol, 85% yield). ^1^H NMR (400 MHz, CDCl_3_): δ 7.36 (d, *J* = 8.7 Hz, 2H), 6.87 (s, 1H), 6.81 (d, *J* = 8.7 Hz, 2H), 6.62 (s, 1H), 2.80 (t, *J* = 7.5 Hz, 2H), 2.74 (t, *J* = 7.5 Hz, 2H), 2.04 (p, *J* = 7.5 Hz, 2H), 1.98 (s, 3H), 1.89 (s, 3H), 0.99 (s, 9H), 0.21 (s, 6H). ^13^C{^1^H} NMR (101 MHz, CDCl_3_): δ 155.2, 140.0, 136.3, 135.6, 135.4, 133.7, 133.6, 133.4, 128.0, 127.0, 126.6, 125.1, 122.9, 120.5, 38.6, 38.5, 25.8, 23.1, 18.4, 14.5, 14.4, −4.2. IR (ATR): 2930, 2858, 1606, 1512, 1473, 1441, 1363, 1255, 1217, 1168, 1104, 1007, 972, 910, 828, 807, 782, 755, 669 cm^–1^. HRMS (ESI): *m*/*z* calcd for C_27_H_34_ClOS_2_Si^+^: 501.1503 [M-H]^+^; found 501.1494.

#### 4-(2-(5-(4-Hydroxyphenyl)-2-methylthiophen-3-yl)cyclopent-1-en-1-yl)-5-methylthiophene-2-carbaldehyde (**DAE2o**)

In a 100 mL Schlenk tube, a solution of 0.249 g of **2** (0.50 mmol) and 20 mL of anhydrous THF was cooled down to −78 °C under inert atmosphere. 0.5 mL of a 1.7 M *tert-*butyllithium solution in pentane (0.9 mmol) was added dropwise during a minute under vigorous stirring. The solution was stirred for 30 min and then 0.35 mL of anhydrous DMF (4.52 mmol) were added at once. The solution was stirred until it reached room temperature, and then it was poured onto 50 mL water and the mixture was extracted with 2 × 50 mL of Et_2_O. After solvent removal, the resulting mixture was dissolved in 15 mL of CHCl_3_ containing 0.230 g of TBAF (0.85 mmol) and 60 μL of acetic acid. One hour later, the solution was washed twice with 10 mL of water and dried with anhydrous Na_2_SO_4_. After filtering and removing the solvent under vacuum, the crude was purified through flash column chromatography (silica gel, hexane/EtOAc, 3:1) obtaining 0.171 g of pure **DAE2o** (0.45 mmol, 90% yield). ^1^H NMR (400 MHz, CDCl_3_): δ 9.71 (s, 1H, CHO), 7.51 (s, 1H, H-18), 7.34 (d, *J* = 8.6 Hz, 2H, H-3 and H-5), 6.88–6.81 (m, 3H, H-8, H-2 and H-6), 2.88–2.76 (m, 4H, (H-14 and H-16), 2.15–2.03 (m, 5H, H-15 and H-21), 1.93 (s, 3H, H-11). ^13^C{^1^H} NMR (101 MHz, CDCl_3_): δ 183.3 (CHO), 155.6 (C-1), 147.6 (C-20), 140.5 (C-7), 139.4 (C-17), 139.0 (C-19), 138.2 (C-18), 136.9 (C-12 or C-13), 135.9 (C-10), 133.3 (C-9), 132.9 (C-12 or C-13), 127.0 (C-4), 126.9 (C-3 and C-5), 122.4 (C-8), 115.9 (C-2 and C-6), 38.6 (C-14 or C-16), 38.3 (C-14 or C-16), 23.0 (C-15), 15.6 (C-21), 14.3 (C-11). IR (ATR): 3307, 2915, 2842, 2051, 1640, 1610, 1513, 1434, 1369, 1252, 1166, 1142, 1103, 1026, 948, 919, 824, 752, 721, 650 cm^–1^. HRMS (ESI): *m*/*z* calcd for C_22_H_21_O_2_S_2_^+^: 381.0977 [M-H]^+^; found 381.0978.

#### 2,2,2-Trifluoro-1-(4-(2-(5-(4-hydroxyphenyl)-2-methylthiophen-3-yl)cyclopent-1-en-1-yl)-5-methylthiophen-2-yl)ethan-1-one (**DAE3o**)

In a 100 mL Schlenk tube, a solution of 0.125 g of **2** (0.25 mmol) and 20 mL of anhydrous THF was cooled down to −78 °C under inert atmosphere. 0.25 mL of a 1.7 M *tert-*butyllithium solution in pentane (0.43 mmol) was added dropwise during a minute under vigorous stirring. The solution was stirred for 30 min and then 0.15 mL of anhydrous ethyl trifluoroacetate were added at once (1.26 mmol). The solution was stirred until it reached room temperature, and then it was poured onto 50 mL of water and the mixture was extracted with 2 × 50 mL of Et_2_O. After solvent removal, the resulting mixture was dissolved in 15 mL of CHCl_3_ containing 0.120 g of TBAF (0.45 mmol) and 30 μL of acetic acid. One hour later, the solution was washed twice with 10 mL of water and dried with anhydrous Na_2_SO_4_. After removing the solvent under vacuum, the crude was purified through flash column chromatography (silica gel, hexane/EtOAc, 9:1) obtaining 0.041 g of pure **DAE3o** (0.090 mmol, 35% yield). ^1^H NMR (250 MHz, CDCl_3_): δ 7.67 (s, 1H, H-18), 7.35 (d, *J* = 8.7 Hz, 2H, H-3 and H-5), 6.87–6.75 (m, 3H, H-2, H-6 and H-8), 4.86 (s, 1H, OH), 2.83 (t, *J* = 6.7 Hz, 4H, H-14 and H-16), 2.22–1.99 (m, 5H, H-15 and H-21), 1.95 (s, 3H, H-11). ^19^F NMR (235 MHz, CDCl_3_): δ −72.38 (CF_3_). ^13^C{^1^H} NMR (91 MHz, CDCl_3_): δ 173.1 (q, *J* = 36.2 Hz, CO), 155.2 (C-1), 150.4 (C-20), 140.6 (C-7), 139.1 (C-18), 138.3 (q, *J* = 2.9 Hz, C-17), 137.7 (C-12 or C-13), 135.8 (C-10), 133.6 (C-9), 132.6 (C-12 or C-13), 132.0 (C-19), 127.4 (C-4), 127.0 (C-3 and C-5), 122.5 (C-8), 116.6 (q, *J* = 290.8 Hz, CF_3_), 115.8 (C-2 and C-6), 38.6 (C-14 and C-16), 38.4 (C-14 and C-16), 23.1 (C-15), 15.6 (C-21), 14.4 (C-11). IR (ATR): 3368, 2920, 2051, 1679, 1611, 1514, 1432, 1371, 1195, 1141, 1045, 948, 926, 869, 824, 754, 718, 678, 660 cm^–1^. HRMS (ESI): *m*/*z* calcd for C_23_H_18_F_3_O_2_S_2_^–^: 447.0706 [M]^−^; found 447.0692.

#### 4-(5-Methyl-4-(2-(2-methyl-5-(perfluorophenyl)thiophen-3-yl)cyclopent-1-en-1-yl)thiophen-2-yl)phenol (**DAE4o**)

In a 100 mL Schlenk tube, a solution of 0.103 g of **2** (0.22 mmol) and 10 mL of anhydrous THF was cooled down to −78 °C under inert atmosphere. Under vigorous stirring, 0.25 mL of a 1.7 M *tert-*butyllithium solution in pentane (0.43 mmol) were added. The solution was stirred for 30 min and then 0.1 mL of perfluorobenzene (0.85 mmol) were added at once. The solution was stirred until it reached room temperature, and then it was poured onto 20 mL of water. The mixture was then extracted with 2 × 50 mL of Et_2_O. After solvent removal, the resulting mixture was dissolved in 10 mL of CHCl_3_ containing 0.120 g of TBAF (0.45 mmol) and 30 μL of acetic acid. One hour later, the solution was washed twice with 10 mL of water and dried with anhydrous Na_2_SO_4_. After filtration and removal of the solvent, the crude was purified through flash column chromatography (silica gel, hexane:AcOEt 9:1) to obtain 0.083 g of **DAE4o** as a white powder (0.16 mmol, 74% yield). ^1^H NMR (600 MHz, CDCl_3_): δ 7.36 (d, *J* = 8.8 Hz, 2H, H-3 and H-5), 7.20 (s, 1H, H-18), 6.86 (s, 1H, H-8), 6.80 (d, *J* = 8.8 Hz, 2H, H-2 and H-6), 4.85 (s, 1H, OH), 2.84 (t, *J* = 7.5 Hz, 4H, H-14 and H-16), 2.14–2.04 (m, 5H, H-15 and H-21), 1.97 (s, 3H, H-11). ^19^F NMR (235 MHz, CDCl_3_): δ −140.81 (dd, *J*_*1*_ = 22.2, *J*_*2*_ = 6.8 Hz, 2F, F-23 and F-27), −157.41 (t, *J* = 21.1 Hz, 1F, F-25), −162.92 (td, *J*_*1*_ = 22.0, *J*_*2*_ = 7.0 Hz, 2F, F-24 and F-26). ^13^C{^1^H} NMR (151 MHz, CDCl_3_): δ 154.9 (C-1), 144.0 (dm, *J* = 250.0 Hz, C-23 and C-27) 139.8 (C-7), 139.7 (dm, *J* = 236.0 Hz, C-25) 138.2 (t, *J* = 3.3 Hz, C-17), 138.0 (dm, *J* = 234.3 Hz, C-24 and C-26), 136.5 (C-20), 136.3 (C-10), 135.8 (C-12 or C-13), 133.9 (C-12 or C-13), 133.6 (C-9), 131.6 (t, *J* = 5.1 Hz, C-18), 127.8 (C-4), 127.0 (C-3 and C-5), 123.0 (C-8), 122.0 (C-19) 115.8 (C-2 and C-6), 110.3 (td, *J* = 15.2, 3.9 Hz, C-22), 38.6 (C-14 or C-16), 38.5 (C-14 or C-16), 23.1 (C-15), 14.4 (C-11 or C-21), 14.3 (C-11 or C-21). IR (ATR): 3339, 2920, 2849, 2325, 2051, 1706, 1611, 1516, 1494, 1437, 1371, 1242, 1171, 1104, 1036, 986, 825, 767, 740, 631 cm^–1^. HRMS (ESI): *m*/*z* calcd for C_27_H_18_F_5_OS_2_^–^: 517.0725 [M]^−^; found 517.0704.

#### 4-(5-Methyl-4-(2-(2-methyl-5-(4-nitrophenyl)thiophen-3-yl)cyclopent-1-en-1-yl)thiophen-2-yl)phenol (**DAE5o**)

A solution of 0.100 g of **2** (0.20 mmol) in 10 mL of anhydrous THF was cooled down to −78 °C before adding dropwise 0.5 mL of a 1.7 M solution of *tert*-butyllithium in pentane (0.85 mmol). After stirring for 30 min at −78 °C, the mixture was quenched with 0.5 mL of tributyl borate (1.85 mmol) and stirred for additional 30 min until it reached room temperature. Then the solution was added onto a degassed mixture of 0.080 g of 4-iodo-1-nitrobenzene (0.32 mmol), 0.040 g of Pd(PPh_3_)_4_ (0.03 mmol) in 10 mL of THF, and 15 mL of a 2 M Na_2_CO_3_ aqueous solution. The two-phase system was then heated with a metallic heat-on block under reflux for 2 h. Once cooled down the reaction mixture, 25 mL of water and 25 mL of Et_2_O were added and the organic layer was separated by extraction. It must be noted that during the Suzuki reaction, the phenol moiety was partially deprotected. To ensure complete silane removal, the organic phase was washed with a 0.1 M TBAF aqueous solution, and after drying with anhydrous Na_2_SO_4_, filtration and further removal of the solvent under vacuum, a brownish oil was obtained. The crude was then purified through flash column chromatography (silica gel, hexane/EtOAc, 9:1) to yield 0.044 mg of compound **DAE5o** (0.092 mmol, 46% yield). ^1^H NMR (400 MHz, CDCl_3_): δ 8.18 (d, *J* = 8.8 Hz, 2H, H-24 and H-26), 7.59 (d, *J* = 8.8 Hz, 2H, H-23 and H-27), 7.37 (d, *J* = 8.6 Hz, 2H, H-3 and H-5), 7.18 (s, 1H, H-18), 6.89 (s, 1H, H-8), 6.81 (d, *J* = 8.6 Hz, 2H, H-2 and H-6), 2.85 (t, *J* = 7.4 Hz, 4H, H-14 and H-16), 2.10 (p, *J* = 7.5 Hz, 2H, H-15), 2.06 (s, 3H, H-21), 1.98 (s, 3H, H-11). ^13^C{^1^H} NMR (151 MHz, CDCl_3_): δ 155.1 (C-1), 146.3 (C-25), 140.9 (C-22), 140.0 (C-7), 138.2 (C-17), 137.8 (C-20), 137.0 (C-22), 136.4 (C-10), 135.9 (C-12 or C-13), 133.9 (C-12 or C-13), 133.6 (C-9), 127.6 (C-4), 127.1 (C-18), 127.0 (C-3 and C-5), 125.4 (C-23 and C-27), 124.5 (C-24 and C-26), 122.9 (C-8), 115.8 (C-2 and C-6), 38.6 (C-14 or C-16), 38.5 (C-14 or C-16), 23.2 (C-15), 14.8 (C-21), 14.5 (C-11). IR (ATR): 3329, 2919, 2850, 2051, 1700, 1593, 1510, 1435, 1335, 1261, 1170, 1109, 950, 825, 750, 691, 668 cm^–1^. HRMS (ESI): *m*/*z* calcd for C_27_H_22_NO_3_S_2_^–^: 472.1047 [M]^−^; found 472.1051.

#### 1-(2-Methyl-5-(4-hydroxyl)phenyl)thien-3-yl)-2-(5-pyridyl-2-methylthien-3-yl)cyclopentene (**3**)

A solution of 0.228 g of **2** (0.46 mmol) in 30 mL of anhydrous THF was cooled down to −78 °C before adding dropwise 0.65 mL of a 1.7 M solution of *tert-*butyllithium in pentane (0.94 mmol). After stirring for 30 min at −78 °C, the mixture was quenched with 0.25 mL of tributyl borate (0.93 mmol) and stirred for 30 min more until it reached room temperature. Later the solution was added onto a degassed mixture of 0.107 g of 4-bromopyridinium hydrochloride (0.55 mmol) and 0.038 mg of Pd(PPh_3_)_4_ (0.03 mmol) in 25 mL of THF and 25 mL of a 2 M Na_2_CO_3_ aqueous solution. The two-phase system was then heated metallic with a metallic heat-on block under reflux for 2 h. Afterward, and once cooled down the reaction mixture, 50 mL of water and 50 mL of Et_2_O were added and the organic layer was separated by extraction. It must be noted that during the Suzuki coupling reaction, the phenol moiety was partially deprotected. To ensure complete silane removal, the organic phase was washed with 0.1 M TBAF aqueous solution, and after drying with anhydrous Na_2_SO_4_, filtration and further removal of the solvent under vacuum, a brownish oil was obtained. The crude was then purified through flash column chromatography (silica gel, EtOAc/hexane 1:3) to yield 0.106 g of compound **3** (0.25 mmol, 54% yield). ^1^H NMR (400 MHz, CDCl_3_): δ 8.50 (d, *J* = 6.0 Hz, 2H), 7.37 (d, *J* = 6.0 Hz, 2H), 7.32 (d, *J* = 8.6 Hz, 2H), 7.22 (s, 1H), 6.84–6.79 (m, 3H), 2.88–2.80 (m, 4H), 2.13–2.06 (m, 5H), 2.04 (s, 3H). ^13^C{^1^H} NMR (101 MHz, CDCl_3_): δ 156.1, 149.8, 142.2, 140.1, 137.9, 137.6, 136.4, 136.1, 136.0, 134.0, 133.4, 127.1, 127.0, 126.9, 122.9, 119.7, 116.0, 38.4, 38.4, 23.2, 14.8, 14.5. IR (ATR): 3061, 2916, 2845, 1774, 1598, 1514, 1437, 1371, 1273, 1249, 1217, 1169, 1104, 1065, 1006, 947, 908, 816, 756, 730, 665, 636 cm^–1^. HRMS (ESI): *m*/*z* calcd for C_26_H_24_NOS_2_^+^: 430.1294 [M-H]^+^; found 430.1294.

#### 3-(4-(4-(2-(5-(4-Hydroxyphenyl)-2-methylthiophen-3-yl)cyclopent-1-en-1-yl)-5-methylthiophen-2-yl)pyridin-1-ium-1-yl)propane-1-sulfonate (**DAE6o**)

0.086 g of **3** (0.20 mmol) were heated with a metallic heat-on block under reflux in acetonitrile overnight with 20 μL of 1,3-propanesultone (0.23 mmol). The resulting precipitate was isolated through filtration and washed with acetone twice obtaining 0.034 g of a light brown powder identified as **DAE6o** (0.06 mmol, 30% yield). ^1^H NMR (400 MHz, DMSO-*d*_6_): δ 9.58 (s, 1H, OH), 8.89 (d, *J* = 6.8 Hz, 2H, H-22 and H-26), 8.19 (d, *J* = 6.8 Hz, 2H, H-23 and H-25), 8.13 (s, 1H, H-18), 7.32 (d, *J* = 8.5 Hz, 2H, H-3 and H-5), 7.03 (s, 1H, H-8), 6.75 (d, *J* = 8.5 Hz, 2H, H-2 and H-6), 4.61 (t, *J* = 6.7 Hz, 2H, H-27), 2.85 (t, *J* = 7.3 Hz, 4H, H-14 and H-16), 2.44 (t, *J* = 6.9 Hz, 2H, H-29), 2.21 (p, *J* = 6.8 Hz, 2H, H-28), 2.07 (p, *J* = 7.5 Hz, 2H, H-15), 2.00 (s, 3H, H-21) 1.89 (s, 3H, H-11). ^13^C{^1^H} NMR (101 MHz, DMSO-*d*_6_): δ 157.0 (C-1), 147.6 (C-24), 144.7 (C-22 and C-26), 143.4 (C-20), 139.8 (C-7), 139.0 (C-19), 136.0 (C-10), 135.9 (C-12 or C-13), 133.3 (C-18), 132.8 (C-17), 132.7 (C-12 or C-13), 131.8 (C-9), 126.3 (C-3 and C-5), 124.7 (C-4), 122.2 (C-8), 121.5 (C-23 and C 25), 115.7 (C2 and C-6), 58.3 (C-29), 47.0 (C-27), 38.1 (C-14 or C-16), 38.1 (C-14 or C-16), 27.1 (C-28), 22.3 (C-15), 14.6 (C-21), 14.0 (C-11). IR (ATR): 3047, 2946, 2850, 2112, 1634, 1610, 1548, 1510, 1472, 1436, 1360, 1313, 1282, 1236, 1209, 1164, 1105, 1035, 873, 829, 814, 782, 749, 734, 663, 607 cm^–1^. HRMS (ESI): *m*/*z* calcd for C_29_H_30_NO_4_S_3_^+^: 552.1331 [M-H]^+^; found 552.1317.

#### 3-Iodo-2-methyl-5-nitrothiophene (**7**)

3.312 g of **6** (14.7 mmol) in 50 mL of acetic anhydride were cooled down with an ice-bath. Over a period of 20 min, 2 mL of concentrated nitric acid were added dropwise at 0 °C. Once added, the mixture was stirred at room temperature for 2 h. The solvent was removed under vacuum and after evaporation 30 mL of water and 30 mL of diethyl ether were added. The organic phase was extracted and washed once with 20 mL of water. The organic phase was dried with Na_2_SO_4_, filtered and the solvent removed under vacuum. The product was then purified via flash column chromatography (silica gel, hexane/EtOAc, 49:1) to yield 1.813 g of **7** as a reddish liquid (6.74 mmol, 46% yield). ^1^H NMR (360 MHz, CDCl_3_): δ 7.83 (s, 1H), 2.46 (s, 3H). ^13^C NMR (91 MHz, CDCl_3_): δ 150.1, 147.6, 136.1, 80.4, 19.3. IR (ATR): 3086, 2411, 2307, 1717, 1513, 1492, 1422, 1383, 1341, 1317, 1171, 1152, 1096, 991, 857, 818, 777, 729, 707, 639 cm^–1^. HRMS (ESI): *m*/*z* calcd for C_5_H_3_INO_2_S^–^: 267.8935 [M]^−^; found 267.8922

#### 2-Bromo-1-(2-methyl-5-nitro-thien-3-yl)cyclopentene (**9**)

A solution of 1.428 g of **8** (5.58 mmol) in 20 mL of anhydrous THF was cooled down to −78 °C before adding dropwise 3 mL of a 2.5 M solution of *n*-butyllithium in pentane (7.50 mmol). After stirring for 30 min at −78 °C, 2.2 mL of tributyl borate (8.15 mmol) were added and the mixture was stirred for 30 min more until it reached room temperature. Then the reaction mixture was quenched with 25 mL of a 2 M Na_2_CO_3_ aqueous solution_._ Later the resulting solution was added onto a degassed mixture of 1.131 g of **7** (4.2 mmol) and 0.237 g of Pd(PPh_3_)_4_ (0.21 mmol) in 25 mL of THF. The two-phase system was then heated with a metallic heat-on block under reflux for 2 h. Once the reaction mixture had cooled down, 50 mL of water and 50 mL of Et_2_O were added and the organic phase was separated by extractions. The organic phase was then washed with a 0.2 M NaHCO_3_ aqueous solution, and after drying with anhydrous Na_2_SO_4_, filtration and solvent removal under vacuum, a dark oil was obtained. The crude was then purified through flash column chromatography (silica gel, hexane/EtOAc, 49:1) to obtain 0.957 g of **9** as a brownish liquid (3.42 mmol, 81% yield). ^1^H NMR (250 MHz, CDCl_3_): δ 7.76 (s, 1H), 2.82 (tt, *J*_*1*_ = 7.5 Hz, *J*_*2*_ = 2.5 Hz, 2H), 2.61 (tt, *J*_*1*_ = 7.5 Hz, *J*_*2*_ = 2.5 Hz, 2H), 2.45 (s, 3H), 2.09 (p, *J* = 7.5 Hz, 2H). ^13^C{^1^H} NMR (63 MHz, CDCl_3_): δ 147.9, 145.0, 134.5, 134.3, 129.5, 122.0, 41.0, 36.8, 22.5, 15.7. IR (ATR): 2921, 2848, 1649, 1499, 1420, 1375, 1322, 1234, 1201, 1153, 1093, 1029, 896, 869, 815, 768, 736, 710, 656, 633 cm^–1^. HRMS (APCI): *m*/*z* calcd for C_10_H_9_BrNO_2_S^–^: 285.9543 [M]^−^; found 285.9553.

#### 3-Bromo-5-(4-hydroxylphenyl)-2-methylthiophene (**11**)

A solution of 2.003 g of **10** (7.84 mmol) in 20 mL of anhydrous THF was cooled down to −78 °C before adding dropwise 4 mL of a 2.5 M solution of *n*-butyllithium in pentane (10 mmol). After stirring for 30 min at −78 °C, the mixture was quenched with 3 mL of tributyl borate (11.1 mmol) and stirred for 30 min more until it reached room temperature. Then the resulting solution was added onto a degassed two-phase mixture of 1.585 g of 4-iodophenol (7.20 mmol) and 0.295 g of Pd(PPh_3_)_4_ (0.022 mmol) in 25 mL of THF and 40 mL of a 2 M Na_2_CO_3_ aqueous solution. The two-phase system was then heated with a metallic heat-on block under reflux for 2 h. When the mixture reached room temperature, 25 mL of water and 25 mL of Et_2_O were added and the organic layer was separated by extraction. The organic phase was then washed with 0.2 M NaHCO_3_ aqueous solution. After drying with anhydrous Na_2_SO_4_, filtration and further removal of the solvent under vacuum, a red oil was obtained. The crude was then purified through flash column chromatography (silica gel, EtOAc/hexane, 1:9) to yield 1.311 g of **11** (4.83 mmol, 67% yield). ^1^H NMR (250 MHz, CDCl_3_): δ 7.38 (d, *J* = 8.7 Hz, 2H), 6.98 (s, 1H), 6.83 (d, *J* = 8.7 Hz, 2H), 4,91 (bs, 1H), 2.40 (s, 3H). ^13^C{^1^H} NMR (101 MHz, CDCl_3_): δ 155.5, 141.1, 132.8, 127.1, 126.8, 124.7, 116.0, 109.8, 14.9. IR (ATR): 3313, 2921, 2053, 1886, 1610, 1545, 1512, 1442, 1381, 1290, 1254, 1178, 1108, 1016, 949, 813, 791, 757, 705, 670 cm^–1^. HRMS (ESI): *m*/*z* calcd for C_11_H_8_BrOS^–^: 266.9485 [M]^−^; found 266.9492.

#### 3-Bromo-5-(4-(tertbutyldimethylsyliloxy)phenyl)-2-methylthiophene (**12**)

0.152 g of **11** (0.57 mmol), 0.090 g of TBSCl (0.60 mmol) and 0.040 g of imidazole (0.062 mmol) were stirred in 5 mL of anhydrous CH_2_Cl_2_ at room temperature overnight. Then the resulting organic solution was washed with 20 mL of water. The organic phase was dried over Na_2_SO_4_, filtered and the solvent removed under vacuum. Compound **12** was then purified via flash column chromatography (silica gel, hexane) to obtain 0.173 g (0.45 mmol, 80% yield). ^1^H NMR (250 MHz, CDCl_3_): δ 7.37 (d, *J* = 8.7 Hz, 2H), 6.99 (s, 1H), 6.84 (d, *J* = 8.7 Hz, 2H), 2.40 (s, 3H), 1.00 (s, 9H), 0.22 (s, 6H). ^13^C{^1^H} NMR (63 MHz, CDCl_3_): δ 155.8, 141.3, 132.7, 127.1, 126.7, 124.7, 120.6, 109.7, 25.8, 18.4, 14.9, −4.2. IR (ATR): 2929, 2857, 1604, 1509, 1473, 1362, 1255, 1172, 1103, 1007, 905, 838, 821, 806, 781, 730, 674, 649 cm^–1^. HRMS (ESI): *m*/*z* calcd for C_17_H_23_BrOSSi^–^: 382.0417 [M]^−^; found 382.0419.

#### 4-(5-Methyl-4-(2-(2-methyl-5-nitrothiophen-3-yl)cyclopent-1-en-1-yl)thiophen-2-yl)phenol (**DAE7o**)

A solution of 0.61 g of **12** (1.59 mmol) in 20 mL of anhydrous THF was cooled down to −78 °C before adding dropwise 0.75 mL of a 2.5 M solution of *n*-butyllithium in pentane (1.88 mmol). After stirring for 30 min at −78 °C, 0.50 mL of tributyl borate (1.69 mmol) were added and the mixture was stirred for 30 min more until it reached room temperature. Then the reaction mixture was quenched with 25 mL of 2 M Na_2_CO_3_ aqueous solution. Later the resulting mixture was added onto a degassed mixture of 0.480 g of **9** (1.66 mmol) and 0.058 g of Pd(PPh_3_)_4_ (0.05 mmol) in 25 mL of THF. The two-phase system was then heated with a metallic heat-on block under reflux for 2 h. Afterward, once cooled down the crude, 25 mL of water and 25 mL of Et_2_O were added and the organic layer was separated after extractions. It must be noted that the phenol moiety was partially deprotected during the Suzuki coupling reaction. To ensure complete silane removal, the organic phase was then washed with a 0.1 M TBAF aqueous solution, and after drying with anhydrous Na_2_SO_4_, filtration and further removal of the solvent under vacuum, a dark oil was obtained. The crude was then purified through flash column chromatography (silica gel, hexane/EtOAc, 9:1) to obtain 0.331 g of **DAE7o** as a brownish oil (0.80 mmol, 52% yield). ^1^H NMR (400 MHz, CDCl_3_): δ 7.70 (s, 1H, H-18), 7.35 (d, *J* = 8.6 Hz, 2H, H-3 and H-5), 6.88–6.76 (m, 3H, H-2, H-6 and H-8), 5.05 (bs, 1H, OH), 2.89–2.73 (m, 4H, H-14 and H-16), 2.09 (p, *J* = 7.7 Hz, 2H, H-15), 1.99 (s, 3H, H-11 or H-21), 1.98 (s, 3H, H-11 or H-21). ^13^C{^1^H} NMR (101 MHz, CDCl_3_): δ 155.2 (C-1), 147.8 (C-17), 144.4 (C-20), 140.6 (C-7), 138.0 (C-12 or C-13), 136.6 (C-19), 135.7 (C-10), 133.6 (C-9), 132.3 (C-12 or C-13), 129.9 (C-18), 127.4 (C-4), 127.0 (C-3 and C-5), 122.5 (C-8), 115.9 (C-2 and C-6), 38.7 (C-14 or C-16), 38.5 (C-14 or C-16), 23.0 (C-15), 15.3 (C-21), 14.5 (C-11). IR (ATR,): 3356, 2920, 2848, 2051, 1707, 1611, 1496, 1432, 1374, 1319, 1264, 1168, 1094, 1027, 949, 869, 823, 757, 736, 656 cm^–1^. HRMS (ESI): *m*/*z* calcd for C_21_H_18_NNaO_3_S_2_^+^: 420.0698 [M-Na]^+^; found 420.0705.

#### 1-(5-Chloro-2-methylthien-3-yl)-2-(5-(4-tert-butyldimethylsilyloxy)phenyl)-2-methylthien-3-yl)-3,3,4,4,5,5-hexafluorocyclopentene (**15**)

In a 100 mL Schlenk tube, a solution of 0.231 g of **12** (0.60 mmol) and 10 mL of anhydrous THF was cooled down to −78 °C under inert atmosphere. Under vigorous stirring, 0.25 mL of a 2.5 M *n*-butyllithium solution in pentane (0.75 mmol) were added dropwise during 1 min. The solution was stirred for 30 min after the addition, and 0.147 g of **14** (0.45 mmol) were then added at once. The solution was stirred until it reached room temperature, and then it was poured onto 20 mL of a 2 M aqueous solution of Na_2_CO_3_. Then, the mixture was extracted with Et_2_O (2 × 50 mL). The organic layers were combined and dried with Na_2_SO_4_ and filtered. After removing the solvent, the pale-yellow oil was purified through a flash column chromatography (silica gel, hexane) to isolate 0.073 mg of **15** (0.13 mmol; 29% yield). ^1^H NMR (400 MHz, CDCl_3_): δ 7.40 (d, *J* = 8.8 Hz, 2H), 7.11 (s, 1H), 6.92 (s, 1H), 6.85 (d, *J* = 8.8 Hz, 2H), 1.95 (s, 3H), 1.88 (s, 3H), 0.99 (s, 9H), 0.22 (s, 6H). ^19^F NMR (235 MHz, CDCl_3_): δ −110.60 (m, 4F), −132.34 (p, *J* = 5.3 Hz, 2F). ^13^C{^1^H} NMR (101 MHz, CDCl_3_): δ 156.0, 142.6, 140.6, 140.5, 127.8, 127.0, 126.8, 125.8, 125.5, 124.6, 121.2, 120.8, 25.8, 18.4, 14.6, 14.5. IR (ATR): 2931, 2860, 2051, 1607, 1552, 1512, 1473, 1435, 1362, 1331, 1308, 1264, 1192, 1176, 1105, 1058, 1021, 1001, 979, 953, 911, 823, 806, 778, 735, 720, 678, 666, 620 cm^–1^. HRMS (APCI): *m*/*z* calcd for C_27_H_26_ClF_6_OS_2_Si^–^: 607.0793 [M]^−^; found 607.0765.

#### 2,2,2-Trifluoro-1-(4-(3,3,4,4,5,5-hexafluoro-2-(5-(4-hydroxyphenyl)-2-methylthiophen-3-yl)cyclopent-1-en-1-yl)-5-methylthiophen-2-yl)ethan-1-one (**DAE8o**)

In a 100 mL Schlenk tube, a solution of 0.094 g of **15** (0.15 mmol) in 10 mL of anhydrous THF was cooled to −78 °C under inert atmosphere. Under vigorous stirring, 0.25 mL of a 1.7 M *tert*-butyllithium solution in pentane (0.43 mmol) were added dropwise during a minute. The solution was stirred for 30 min after the addition, when 0.3 mL of anhydrous ethyl trifluoroacetate were added at once (2.5 mmol). The solution was let to reach room temperature, and it was poured onto 20 mL of a 2 M aqueous solution of Na_2_CO_3_. Then, the mixture was extracted with Et_2_O (2 × 50 mL). The organic layers were washed with 20 mL of a 0.1 M TBAF aqueous solution, combined and dried with Na_2_SO_4_. After filtration and solvent removal under vacuum, it was purified through flash column chromatography (silica gel, hexane/EtOAc, 4:1), obtaining 0.012 g of pure **DAE8o** (0.022 mmol, 14% yield). ^1^H NMR (360 MHz, CDCl_3_): δ 7.90 (s, 1H, H-18), 7.40 (d, *J* = 8.5 Hz, 2H, H-3 and H-5), 7.09 (s, 1H, H-8), 6.86 (d, *J* = 8.5 Hz, 2H, H-2 and H-6), 2.13 (s, 3H, H-21), 1.95 (s, 3H, H-11). ^19^F NMR (235 MHz, CDCl_3_): δ −72.81 (s, 3F, CF_3_), −110.44 (m, 2H, F-14 or F-16), −111.00 (m, 2H, F-14 or F-16), −132.30 (m, 2H, F-15). ^13^C{^1^H} NMR (91 MHz, CDCl_3_): δ 173.1 (d, *J* = 37.1 Hz, CO), 155.9 (C-1), 154.9 (C-20), 143.2 (C-7), 140.5 (C-10), 136.6 (C-18), 134.0 (C-17), 127.7 (C-19), 127.4 (C-3 and C-5), 126.1 (C-4), 125.0 (C-9), 120.9 (C-8), 116.3 (q, *J* = 290.2 Hz, CF_3_), 116.1 (C-2 and C-6), 15.6 (C-21), 14.6 (C-11). IR (ATR): 2927, 2051, 1692, 1612, 1542, 1515, 1439, 1339, 1269, 1192, 1139, 1112, 1051, 986, 928, 901, 867, 823, 751, 732, 666, 631 cm^–1^. HRMS (ESI): *m*/*z* calcd for C_23_H_12_F_9_O_2_S_2_^–^: 555.0140 [M]^−^; found 555.0146.

#### 4-(2-(2-Methyl-5-nitrothiophen-3-yl)cyclopent-1-en-1-yl)phenol (**DAE9o**)

Under inert atmosphere, 0.113 g of **16** (0.39 mmol) was dissolved in 5 mL of anhydrous THF and it was cooled down to −78 °C before adding 0.28 mL (0.45 mmol) of a 1.6 M solution of *n*BuLi in hexane dropwise under vigorous stirring. After stirring at −78 °C for 15 min, 0.12 mL of B(OBu)_3_ (0.44 mmol) were added, and the mixture was stirred for additional 30 min until it reached room temperature. Then the reaction mixture was quenched with 5 mL of a 2 M Na_2_CO_3_ aqueous solution. The resulting solution was added to a degassed solution of 0.087 g of **9** (0.30 mmol) in 5 mL of THF, 0.028 mg of Pd(PPh_3_)_4_ (0.02 mmol) were added and the two-phase system was heated with a metallic heat-on block under reflux for 2 h. After the mixture cooled down, 20 mL of water and 20 mL of Et_2_O were added and the organic phase was extracted, washed with 10 mL of a 0.2 M NaHCO_3_ aqueous solution, and after drying with anhydrous sodium sulfate, filtration and solvent removal under vacuum, a dark orange oil was obtained. The resulting oil was redissolved in 10 mL of CHCl_3_, 0.6 mL of a 1 M solution of TBAF in THF (0.60 mmol) and 60 μL of AcOH (1.0 mmol) were added and the solution was stirred at room temperature. After one hour the solution was washed twice with 10 mL of water, dried with anhydrous sodium sulfate, filtered and the solvent removed under vacuum. The crude was further purified through flash column chromatography (silica gel, hexane/EtOAc, 9:1), to yield 0.080 mg of **DAE9o** (0.26 mmol; 76% yield) as a yellow oil. ^1^H NMR (300 MHz, CDCl_3_): δ 7.73 (s, 1H, H-13), 7.00 (d, *J* = 8.6 Hz, 2H, H-3 and H-5), 6.69 (d, *J* = 8.6 Hz, 2H, H-2 and H-6)), 4.89 (s, 1H, OH), 2.88 (t, *J* = 7.9 Hz, 2H, H-9 or H-11), 2.74 (t, *J* = 7.9 Hz, 2H, H-9 or H-11), 2.06 (p, *J* = 7.9 Hz, 2H, H-10), 1.98 (s, 3H, H-16). ^13^C{^1^H} NMR (75 MHz, CDCl_3_): δ (ppm) 154.6 (C-1), 148.0 (C-15), 144.1 (C-12), 140.7 (C-7), 136.9 (C-14), 130.2 (C-4), 129.9 (C-8), 129.3 (C-13), 128.5 (C-3 and C-5), 115.3 (C-2 and C-6), 39.5 (C-9 or C-11), 37.3 (C-9 or C-11), 22.3 (C-10), 15.0 (C-16). IR (ATR,): 3392, 2935, 2850, 1602, 1514, 1487, 1405, 1388, 1315, 1271, 1215, 1152, 1102, 827, 819, 737, 564, 544, 519, 515, 502 cm^–1^. HRMS (ESI): *m*/*z* calcd for C_16_H_15_NNaO_3_S^+^: 324.0665 [M]^−^; found 324.0674.

### Photochemical Characterization

Photoswitch isomerization was investigated using a VL-6.M UV lamp (λ_exc_ = 312 nm, 6 W), a Nd:YAG pulsed laser (Brilliant, Quantel, λ_exc_ = 355 or 532 nm), diode cw lasers at λ_exc_ = 405 (SciTech) and λ_exc_ = 650 nm (SciTech). Photoisomerization quantum yields were determined using a procedure previously reported (see the SI for a detailed description).^26^ Closed isomer UV–vis absorption spectra were estimated from the UV–vis absorption spectra of the open state and of a mixture of ring-open and ring-closed isomers of known composition. The compositions of PSS_o-c_ in acetonitrile were determined using two different methodologies. For **DAE1**, **DAE2**, **DAE4** and **DAE5**, they were directly obtained by ^1^H NMR or ^19^F NMR from a PSS_o-c_ mixture of the molecular switch generated upon irradiation in CD_3_CN. For DAEs with low solubility in acetonitrile or suffering high photodegradation in this solvent (**DAE3**, **DAE6**, **DAE7**, **DAE8** and **DAE9**), we used ^1^H NMR or ^19^F NMR to determine the composition of PSS_o-c_ (or of another mixture of the ring-open and ring-closed isomers) in a different solvent (toluene-*d*_8_, acetone-*d*_6_, THF-*d*_8_). Then, the UV–vis absorption spectrum of this mixture was measured in acetonitrile to obtain the pure closed isomer absorption spectrum in this solvent. Finally, from the UV–vis absorption spectra of PSS_o-c_ and the ring-open and ring-closed isomers in acetonitrile, the composition of the photostationary state was determined.

### p*K*_a_ Determination

p*K*_a,ACN_ values for **DAE1–9** were obtained by monitoring the variation of the UV–vis absorption of these compounds upon titration with an acetonitrile solution of TBAOH (*c* = 1.0 × 10^–2^ M) while simultaneously measuring . For the open states of **DAE1–9**, these experiments were conducted on pure solutions of their ring-open isomer. In the case of the closed states of **DAE1–9**, spectrophotometric titrations were performed using PSS_o-c_ samples obtained upon UV irradiation of the corresponding open isomer solutions. This was possible thanks to a) as the closed states of **DAE 1–9** have significantly lower p*K*a, they could be selectively deprotonated in our titration experiments on PSS_o-c_ mixtures where their open isomer counterparts were also present; b) both the protonated and deprotonated forms of the closed isomers of **DAE 1–9** present absorption bands in the visible region that can be independently registered. From these measurements, p*K*_a,ACN_ values were then obtained by graphical representation of the following equation:
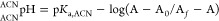
1where A is the absorbance of the sample, A_0_ is the absorbance before base addition, and A_f_ is the absorbance upon complete deprotonation. These absorbance values were taken at the wavelength of the maximum of the bathochromic absorption band measured for the deprotonated forms of the open and closed isomers of **DAE1–9** (Figure S2).

To estimate the p*K*_a,H2O_ values for the open and closed states of **DAE1–9**, eq 2 was used, which was obtained from the correlation between the p*K*_a_ values of several phenols in water and acetonitrile (Figure S6).^20^
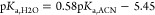
2

## Data Availability

The data underlying this study are available in the published article, its Supporting Information and the open-access repository CORA.RDR at https://doi.org/10.34810/data1876.

## Supporting Information Available

Additional data on experimental and synthetic procedures, photochemical studies, photoswitchable acidity measurements and NMR spectra (PDF)
FAIR data, including the primary NMR FID files, for compounds **1**, **2**, **3**, **5**, **6**, **7**, **9**, **11**, **12**, **14**, **15**, **16**, and **DAE1o–5o** (ZIP)
FAIR data, including the primary NMR FID files, for compounds **DAE6o–9o** (ZIP)
