# Supplementary material for: Amplified Light-Induced pKa Modulation with Diarylethene Photoswitches
Source: J Org Chem. 2024 Nov 30;89(24):17991–8002. doi: 10.1021/acs.joc.4c01606 (PMC11667731; doi:10.1021/acs.joc.4c01606)
Supplement: Supplementary file 1 — jo4c01606_si_001.pdf [file jo4c01606_si_001.pdf]

Supporting Information for:

Amplified Light-Induced pK_a Modulation with Diarylethene Photoswitches

Marc Villabona,[a] Arnau Marco,[a] Rosa Mª Sebastián,[a,b] Gonzalo Guirado,[a,*] and Jordi Hernando[a,*]

[a] *Departament de Química, Universitat Autònoma de Barcelona, Edifici C/n, Campus UAB, 08193 Cerdanyola del Vallès (Spain)*

[b] *Centro de Innovación en Química Avanzada (ORFEO−CINQA), Universitat Autònoma de Barcelona, 08193 Cerdanyola del Vallès (Spain)*

Table of Contents:

2. Design and Synthesis of DAE1-9 **S3**

3. Photochemical Characterization of DAE1-9 **S7**

4. pK_a Determination for DAE1-9 **S10**

5. NMR and IR Spectra of Selected Compounds **S18**

6. References **S58**

1. Design and Synthesis of DAE1-9

1.1. Characteristics of the EWGs introduced in DAE1-9

Table S1. Hammett parameters[a] of the EWGs in **DAE1-9**

Compound	EWG	σ_m	σ_p	Ref.
**DAE1**	**Cl**	0.37	0.23	1
**DAE2**	**CHO**	0.35	0.42	1
**DAE3/DAE8**	**$COCF_3$**	0.63	0.80	1
**DAE4**	**C_6F_5**	0.26	0.27	1
**DAE5**	**4-nitrobenzene**	0.25	0.26	1
**DAE6**	**4-[*N*-(3-sulfopropyl)]pyridinium**[b]	0.62	0.58	1
**DAE7/DAE9**	**NO_2**	0.71	0.78	1

[a] σ_m = Hammett σ-*meta* substituent constant; σ_p = Hammett σ-*para* substituent constant.
[b] σ_m and σ_p values for 2,4,6,-trimethylpyridinium.

1.2. Additional synthetic schemes

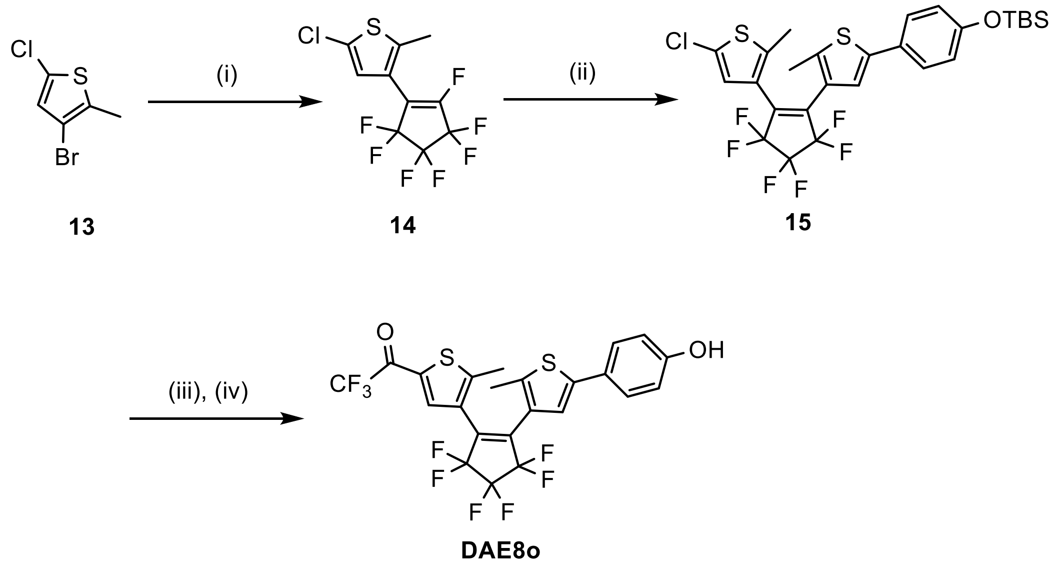

Scheme S1. Synthetic procedure used for the preparation of **DAE8o**. Reagents and conditions: (i) perfluorocylopentene, *n*BuLi, THF, -78ºC (42%); (ii) **12**, *n*BuLi, THF, -78ºC (29%); (iii) *t*BuLi, CF_3COOEt, THF, -78ºC; (iv) TBAF, AcOH, $CHCl_3$, rt (14% over 2 steps). Abreviatures: TBAF: tetrabutylammonium fluoride.

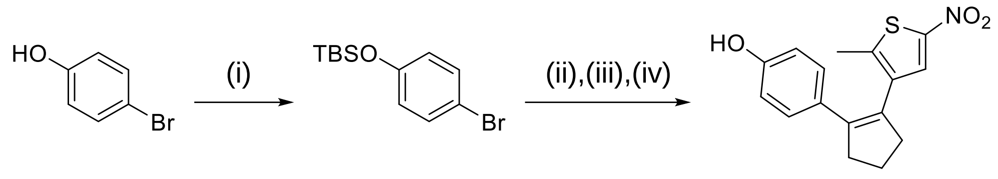

Br-PhOH **16** **DAE9o**

Scheme S2. Synthetic procedure used for the preparation of **DAE9o**. Reagents and conditions: (i) TBSCl, imidazole, CH_2Cl_2, rt (95%); (ii) *n*BuLi, $B(OBu)_3$, THF, -78ºC; (iii) **9**, $Pd(PPh_3)_4$, Na_2CO_3, THF:H_2O, reflux; (iv) TBAF, AcOH, $CHCl_3$, rt (76% over 3 steps). Abreviatures: TBSCl: *tert*-butyldimethylchlorosilane; TBAF: tetrabutylammonium fluoride.

1.3. Synthesis of already reported intermediates

1.3.1. Synthesis of 3,5-diiodo-2-methylthiophene, 5

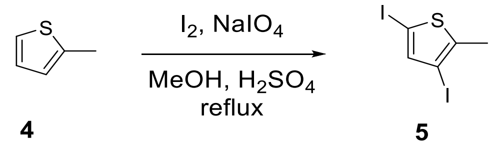

4 **5**

0.595 g of sodium periodate (2.78 mmol) were dissolved in 10 mL of methanol and 2.80 mL of a 2.4 M H_2SO_4 aqueous solution. Over this mixture a solution of 1.325 g of iodine (5.22 mmol) and 0.5 mL of commercially available **4** (5.17 mmol) in 10 mL of methanol was added. The resulting mixture was heated under reflux overnight. After the reaction mixture was cooled down to room temperature, it was mixed with 50 mL of a 2 M aqueous solution of sodium sulphite, and the product was extracted with diethyl ether. The resulting organic layer was washed with distilled water (3 x 20 mL), dried over Na_2SO_4, filtered and then the solvent was evaporated under reduced pressure obtaining 1.804 g of **5** (5.16 mmol, 99% yield). This product was purified by filtration through a short silica pad, and it was employed in the next step without further treatment. The ^{1}H NMR spectrum of this compound matched previously reported data.[2]

^{1}H NMR (360 MHz, $CDCl_3$) δ (ppm) 7.08 (s, 1H), 2.42 (s, 3H).

1.3.2. Synthesis of 3-iodo-2-methylthiophene, 6

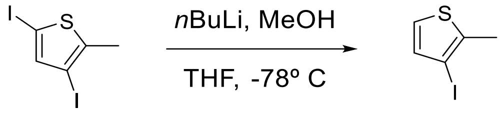

5 **6**

In a 50 mL round bottom flask, 1.804 g of **5** (5.23 mmol) were dissolved in 20 mL of anhydrous THF under inert atmosphere. The solution was cooled down to -78 ºC, and then 2.4 mL of a 2.5 M solution of *n*-butyllithium was added slowly (6.00 mmol). After 15 min stirring at this temperature, 0.5 mL of methanol were added (12.3 mmol) and the mixture was let to reach room temperature. Extractions were then performed with 50 mL of water and 50 mL of diethyl ether and the aqueous phase was washed once with 50 mL of diethyl ether. The combined organic phases were dried over anhydrous Na_2SO_4, filtered and the solvent was removed under vacuum. Finally, the brown liquid was diluted in hexane and filtered through a silica gel plug to obtain 0.945 g of a colorless liquid identified as **6** after solvent removal under vacuum (4.22 mmol, 81% yield). The ^{1}H NMR spectrum of this compound matched previously reported data.[3]

^{1}H NMR (360 MHz, $CDCl_3$): δ (ppm) 7.09 (d, *J* = 5.3 Hz, 1H), 6.97 (d, *J* = 5.2 Hz, 1H), 2.44 (s, 3H).

1.3.3. Synthesis of 1-(5-chloro-2-methylthien-3-yl)-2,3,3,4,4,5,5-heptafluorocyclopentene, 14

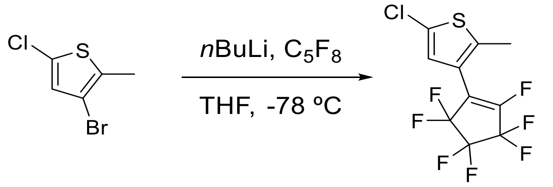

13 **14**

In a 100 mL Schlenk tube, a solution of 0.788 g of **13**[4] (3.73 mmol) in 40 mL of anhydrous THF was cooled down to -78 ºC under inert atmosphere. Under vigorous stirring, 1.6 mL of a 2.5 M *n*-butyllithium solution in pentane (4.00 mmol) were added dropwise during a minute. The solution was stirred during 30 min after the addition, and then 0.96 mL of perfluorocyclopentene (7.15 mmol) were added at once. The solution was let to reach room temperature, and afterwards it was poured onto 50 mL of a 2 M aqueous solution of Na_2CO_3. The reaction was extracted with Et_2O (2 x 50 mL). The organic layers were combined, dried with Na_2SO_4 and filtered. After removing the

solvent, the pale-yellow oil was purified through flash column chromatography (silica gel, hexane) to isolate 0.506 g of a slightly yellow oil **14** (1.56 mmol; 42% yield). The ^{1}H NMR and ^{19}F NMR spectra of this compound matched previously reported data.[2]

^{1}H NMR (360 MHz, $CDCl_3$): δ (ppm) 6.91 (s, 1H), 2.39 (s, 3H).

^{19}F NMR (235 MHz, $CDCl_3$): δ (ppm) -109.03 (d, *J* = 12.0 Hz, 2F), -118.62 (d, *J* = 15.7 Hz, 2F), -126.95 (m, 1F), -130.43 (s, 2F).

1.3.4. Synthesis of (4-bromophenoxy)(*tert*-butyl)dimethylsilane, 17

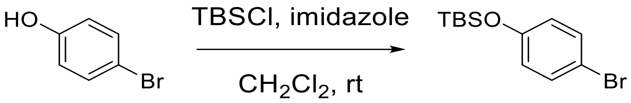

Br-PhOH **16**

A solution of 0.995 g of 4-bromophenol (**Br-PhOH**) (5.75 mmol), 1.182 g of TBSCl (7.84 mmol) and 0.513 g of imidazole (7.54 mmol) in 50 mL of anhydrous dichloromethane was stirred overnight. The mixture was washed with 50 mL of water twice, the organic phase was dried with anhydrous sodium sulfate and solvent was removed under vacuum. The crude further purified through flash column chromatography (silica gel, hexane), yielding 1.566 g of **16** (5.45 mmol; 95% yield) as a colorless oil. The ^{1}H NMR spectrum of this compound matched previously reported data.[5]

^{1}H NMR (300 MHz, $CDCl_3$): δ (ppm) 7.32 (d, *J* = 8.9 Hz, 2H), 6.71 (d, *J* = 8.9 Hz, 2H), 0.97 (s, 9H), 0.18 (s, 6H).

3. Photochemical Characterization of DAE1-9

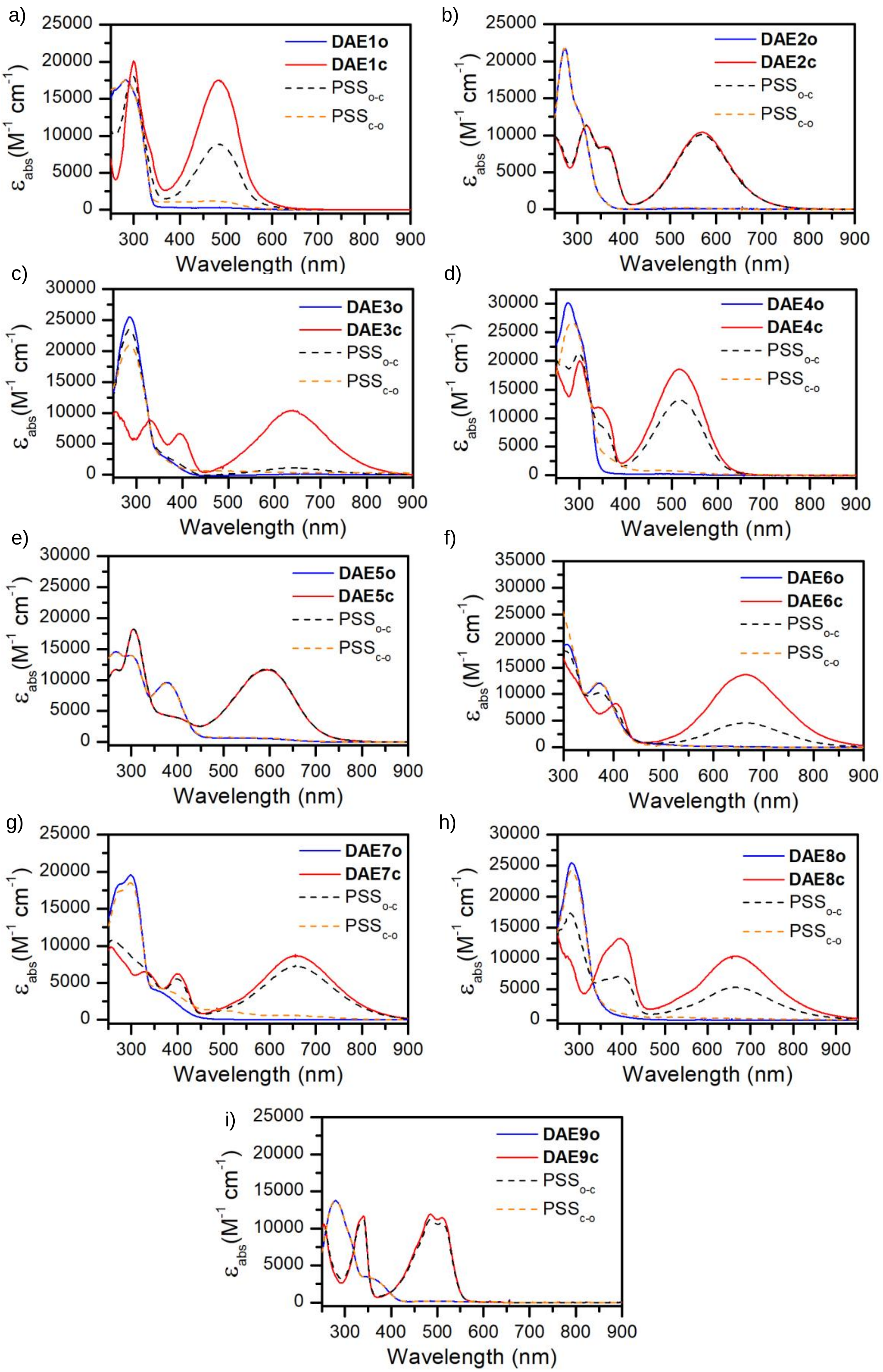

Figure S1. Absorption spectra of the open state (blue), closed state (red), $PSS_{o\text{-}c}$ (dashed black) and $PSS_{c\text{-}o}$ (dashed orange) of (a) **DAE1**, (b) **DAE2**, (c) **DAE3**, (d) **DAE4**, (e) **DAE5**, (f) **DAE6**, (g) **DAE7**, (h) **DAE8** and (i) **DAE9**. For **DAE1-8**, spectra are shown in acetronitrile. For **DAE9**, data is given in cyclohexane, and its $PSS_{o\text{-}c}$ spectrum was measured after letting the irradiated sample in the dark for 2 min to reach the equilibrium of the keto-enol tautomerization of the closed isomer. The irradiation wavelengths used to obtain the $PSS_{o\text{-}c}$ and $PSS_{c\text{-}o}$ states in each case are given in Table 1 in the main text.

Determination of photoisomerization quantum yields: To determine photoisomerization quantum yields, we followed a previously reported procedure.[6] This procedure first required monitoring the variation of the UV-vis absorption spectra of **DAE1-8** in acetonitrile and **DAE9** in cyclohexane upon irradiation with UV (for photocyclization, λ_{exc} = 312 (**DAE1-2**, **DAE4** and **DAE7**), 355 (**DAE5-6**, and **DAE8-9**) or 405 nm (**DAE3**)) or visible light (for photocycloreversion, λ_{exc} = 532 (**DAE1**, **DAE4** and **DAE9**) or 650 nm (**DAE2-3**, **DAE5-9**)). Typically, 10 spectra were taken for each photoisomerization process at different irradiation times. For the analysis of the photocyclization reaction, these times were chosen to be further apart from the irradiation time needed to reach the photostationary state. For each of the spectra registered, two main data points were used in the subsequent mathematical treatment: (a) the absorbance at the excitation wavelength (A_{exc}); and (b) the absorbance at the spectral maximum of the ring-closed isomer of each compound (A_{det}). Then, the concentration of the closed (c_c) and open (c_o) isomers at each irradiation time were determined from A_{det}, the molar absorptivity of the closed isomer at the detection wavelength (ε_o^{det}), and the total DAE concentration which remains essentially constant during irradiation (c_T) (Equation S1). Then, the variation of the concentration of the irradiated isomer (c_R, with R = o or c) was fitted to a simple kinetic model that depends on the photoexcitation intensity (I_0), the optical path (l, with l = 1 cm), the molar absorptivity of that isomer (ε_R^{exc}, with R = o or c) at the excitation wavelength, the total absorbance at the excitation wavelength (A_{exc}), and the quantum yield of the photoreaction (Φ_{ph}) (Equation S2).

$$c_c = A_{det}/(\varepsilon_{\boldsymbol{c}}^{det}\, l) \quad \text{and} \quad c_o = c_T - c_c \qquad \text{(S1)}$$

$$\ln(c_R/c_T) = -\boldsymbol{\phi_{ph}}\, I_0 \varepsilon_R^{exc}\, l \int_{t_0}^{t_i} [(1 - 10^{-A_{exc}})/A_{exc}]\, dt \qquad \text{(S2)}$$

To obtain Φ_{ph} from this mathematical treatment, I_0 was previously determined by monitoring the photocyclization and photocycloreversion processes of DAE reference compounds with known photocyclization and photocycloreversion quantum yields: 1,2-bis(5-chloro-2-methyl-3-thienyl)perfluorocyclopentene ($\Phi_{o\text{-}c}$ = 0.47 and $\Phi_{c\text{-}o}$ = 0.13 in hexane),[7] 1,2-bis(5-formyl-2-methyl-3-thienyl)perfluorocyclopentene ($\Phi_{o\text{-}c}$ = 0.44 and $\Phi_{c\text{-}o}$ = 0.095 in hexane)[8] and 1,2-bis(5-phenyl-2-methyl-3-thienyl)perfluorocyclopentene ($\Phi_{o\text{-}c}$ = 0.59 and $\Phi_{c\text{-}o}$ = 0.013 in hexane).[9]

4. pK_a Determination for DAE1-9

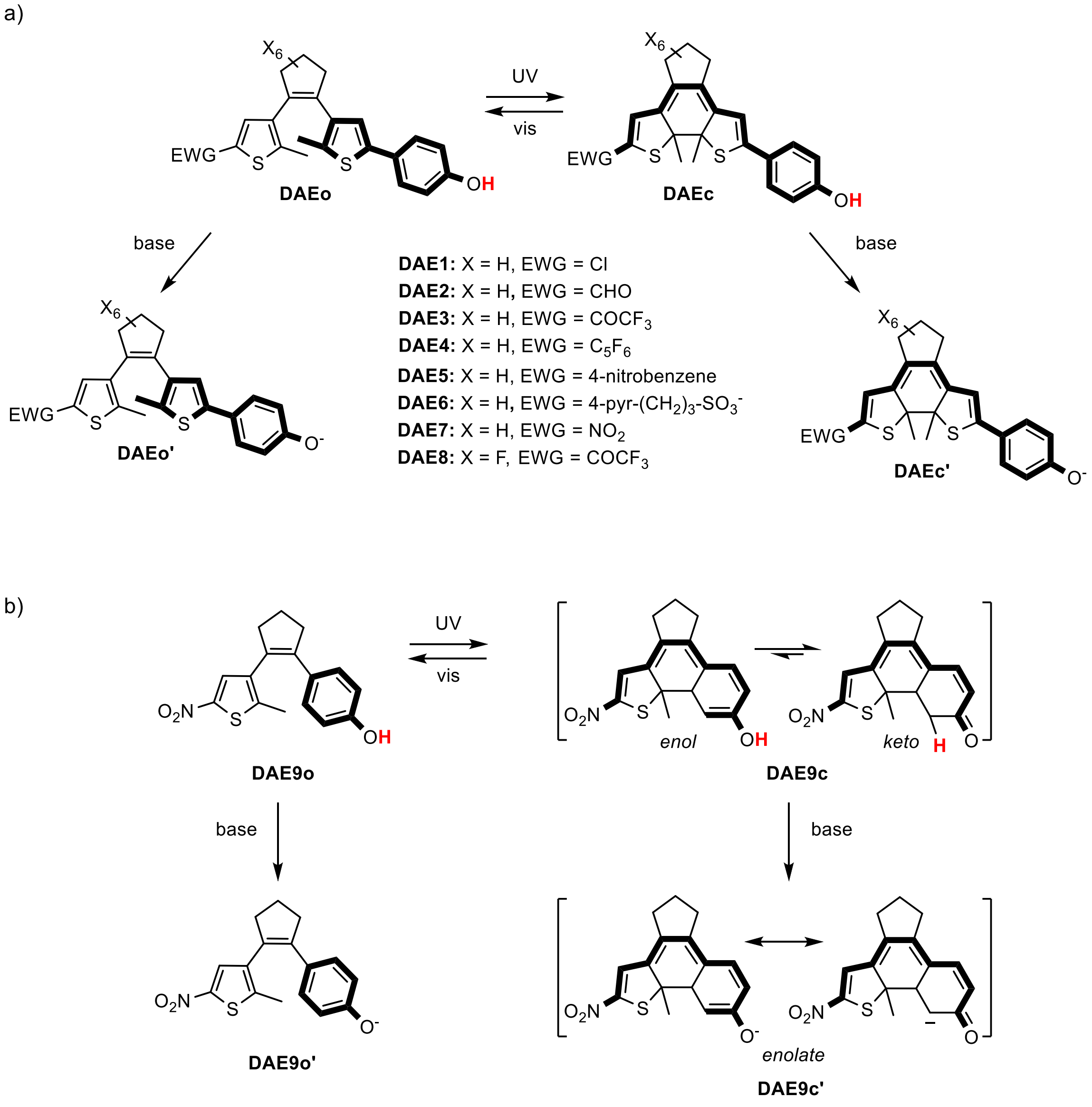

Scheme S3. Deprotonation processes of (a) **DAE1-8** and (b) **DAE9**. In all cases, the negative charge of the phenolate (or enolate) generated upon deprotonation of the closed isomer becomes conjugated with the EWG lying on the other side of the molecules, thus stabilizing this species and increasing the acidity of the starting phenol.

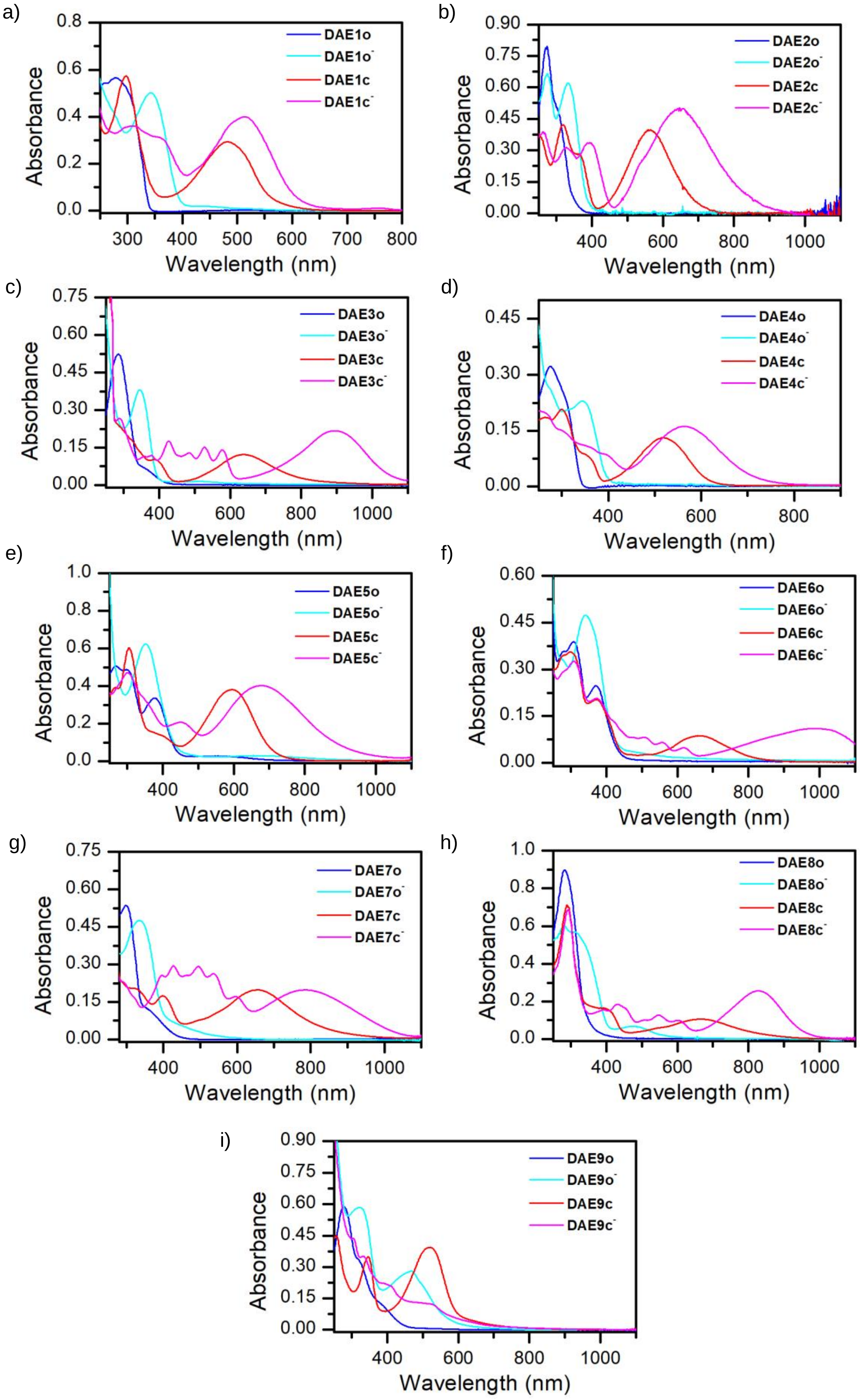
a)
b)
c)
d)
e)
f)
g)
h)
i)
Absorbance
Wavelength (nm)
DAE1o
DAE1o^{-}
DAE1c
DAE1c^{-}
DAE2o
DAE2o^{-}
DAE2c
DAE2c^{-}
DAE3o
DAE3o^{-}
DAE3c
DAE3c^{-}
DAE4o
DAE4o^{-}
DAE4c
DAE4c^{-}
DAE5o
DAE5o^{-}
DAE5c
DAE5c^{-}
DAE6o
DAE6o^{-}
DAE6c
DAE6c^{-}
DAE7o
DAE7o^{-}
DAE7c
DAE7c^{-}
DAE8o
DAE8o^{-}
DAE8c
DAE8c^{-}
DAE9o
DAE9o^{-}
DAE9c
DAE9c^{-}

Figure S2. Absorption spectra in acetonitrile of the open state (DAEo, blue), closed state (DAEc, red), deprotonated open state (DAEo', cyan blue) and deprotonated closed state (DAEc', pink) of (a) **DAE1**, (b) **DAE2**, (c) **DAE3**, (d) **DAE4**, (e) **DAE5**, (f) **DAE6**, (g) **DAE7**, (h) **DAE8** and (i) **DAE9**. In the case of the DAEc and DAEc' states, the spectra provided correspond to the PSS_{o-c} obtained under UV irradiation (DAEc) and its titration with TBAOH until fill deprotonation.

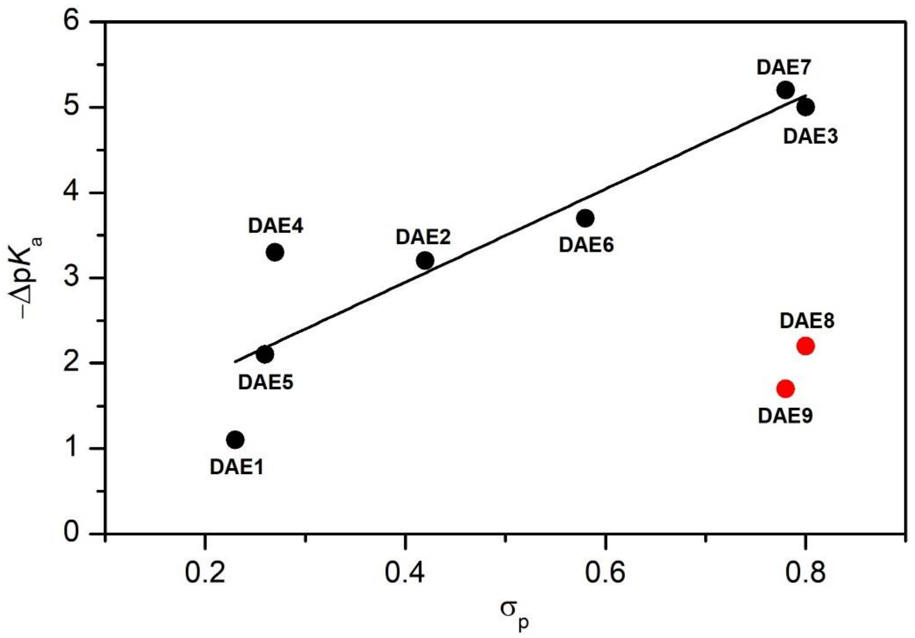

Figure S3. Correlation between ΔpK_a measured in acetonitrile for **DAE1-9** and the Hammett σ-*para* substituent constants reported for the EWGs of these compounds.[1] While reasonable correlation was found for **DAE1-7**, much lower ΔpK_a values than expected were measured for **DAE8-9**, which can be attributed to their particular structural features: (a) the introduction of a perfluorinated cyclopentene in ring in **DAE8**, an inductive EWG that affects the phenol moiety in both the open and closed isomers of this compound and, therefore, dampens the acidity modulation between them; (b) the integration of the phenol unit within the photoswitchable core of **DAE9**, which causes the tautomerization of its closed isomer into a keto form with decreased acidity.

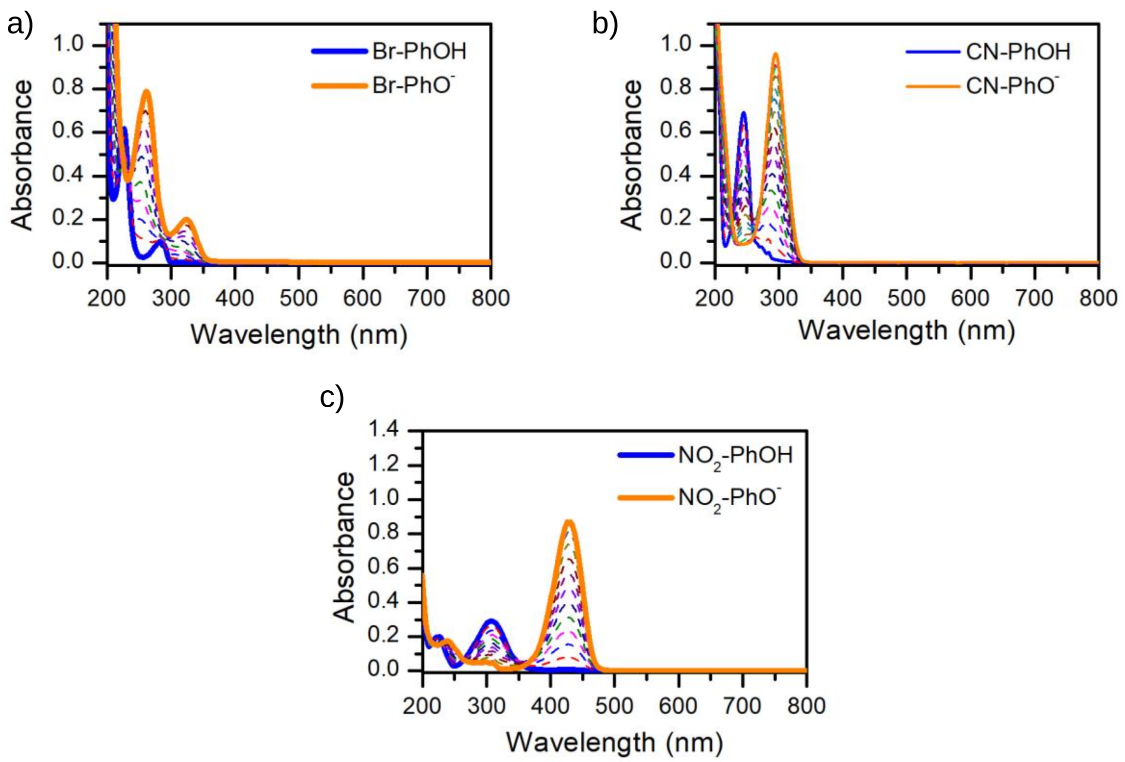

Figure S4. Spectrophotometric titration with TBAOH in acetonitrile of (a) 4-bromophenol (**Br-PhOH**), (b) 4-cyanophenol (**CN-PhOH**) and (c) 4-nitrophenol (**NO_2-PhOH**). Initial absorption spectra are shown in blue (**BrPhOH**, **CN-PhOH** and **NO_2-PhOH**), while the spectra after complete deprotonation are shown in orange (**$BrPhO^-$**, **CN-PhO^-** and **NO_2-PhO^-**).

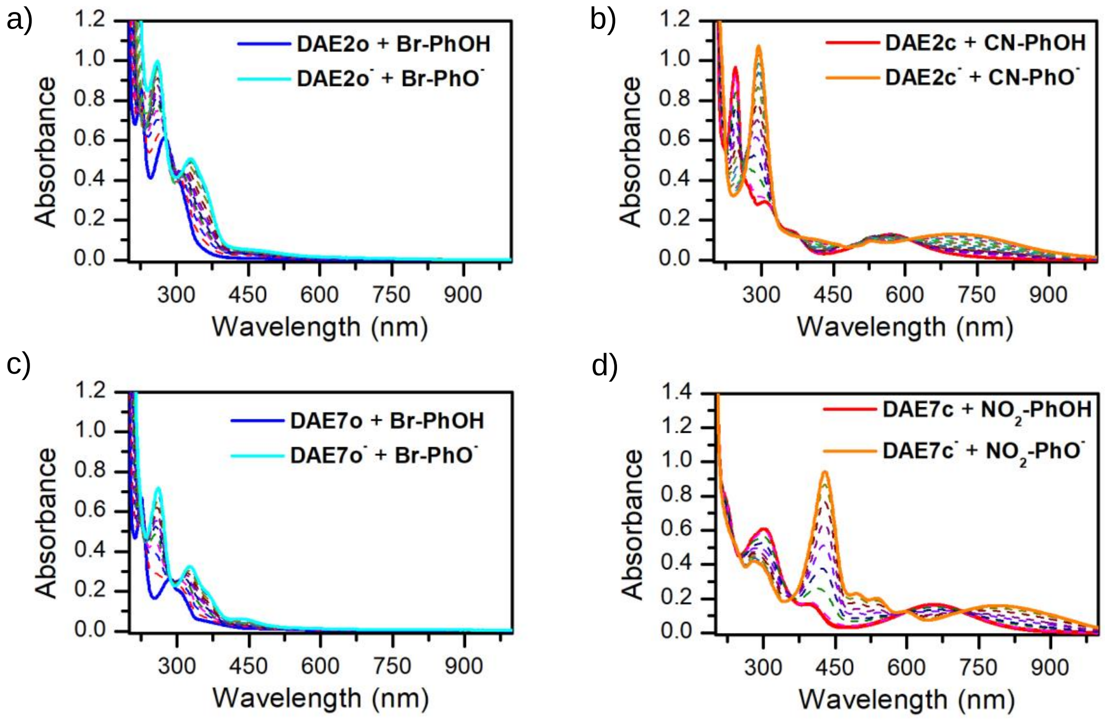

Figure S5. Spectrophotometric titration with TBAOH in acetonitrile of: (a) a mixture of **DAE2o** + **Br-PhOH** (c_{TBAOH} = 0 – 7.5 10^{-4} M with 5.0 10^{-5} M additions); (b) a mixture of **DAE2c** + **CN-PhOH** (c_{TBAOH} = 0 – 7.4 10^{-5} M with 4.64 10^{-6} M additions); (c) a mixture of **DAE7o** + **NO2-PhOH** (c_{TBAOH} = 0 – 5.5 10^{-4} M with 5.5 10^{-5} M additions); and (d) a mixture of **DAE7c** + **NO2-PhOH** (c_{TBAOH} = 0 – 4.8 10^{-5} M with 4.0 10^{-6} M additions). Initial absorption spectra are shown in blue (**DAE2o** + **BrPhOH** and **DAE7o** + **BrPhOH**) and red (**DAE2c** + **CN-PhOH** and **DAE2c** + **NO2-PhOH**), while the spectra after complete deprotonation are shown in cyan (**DAE2o**$^-$ + **BrPhO**$^-$ and **DAE7o**$^-$ + **BrPhO**$^-$) and orange (**DAE2c**$^-$ + **CN-PhO**$^-$ and **DAE2c**$^-$ + **NO2-PhO**$^-$). In all the cases, c_{DAE} =1.5·10^{-5} M and c_{PhOH} = 3.0·10^{-5} M.

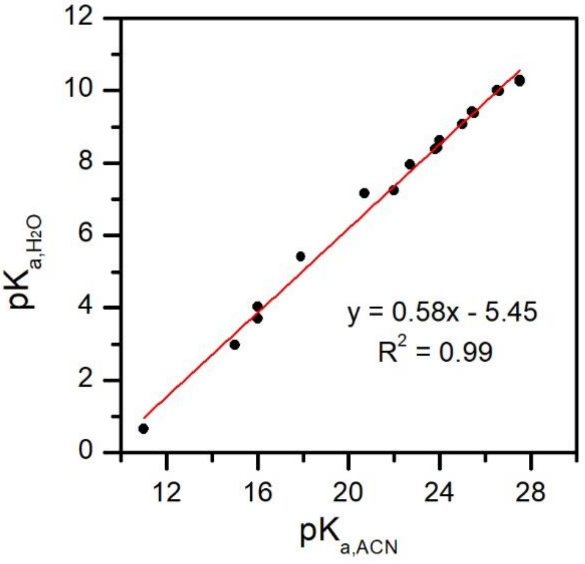

Figure S6. Correlation between p$K_{a,ACN}$ and p$K_{a,H2O}$ for several substituted phenols.[10]

Table S2. Comparison of ΔpK_a values for DAE-based photoswitchable acids.

EWG	**Δp$K_{a,ACN}$** [a]	**Δp$K_{a,H2O}$** [b]	**Ref.**
**DAE1**	-1.1	-0.6	This work
**DAE2**	-3.2	-1.9	This work
**DAE3**	-5.0	-2.9	This work
**DAE4**	-3.3	-1.9	This work
**DAE5**	-2.1	-1.2	This work
**DAE6**	-3.7	-2.1	This work
**DAE7**	-5.2	-3.0	This work
**DAE8**	-2.2	-1.5	This work
**DAE9**	-1.7	-1.0	This work
**DAE^F**	-	-1.2 [c]	11
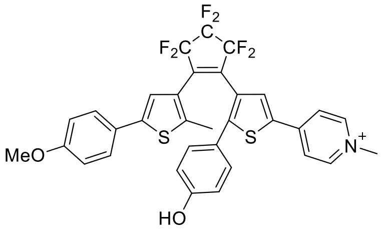	-	-0.4 [c]	12

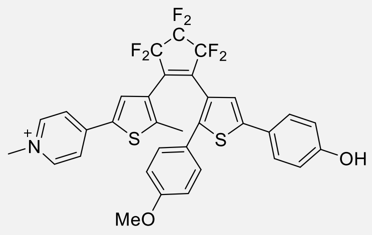	-	-1.2 [c]	12
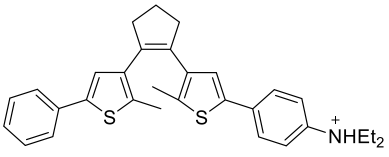	0	-	13
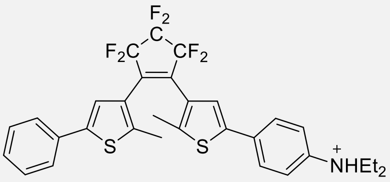	-1.7	-	13
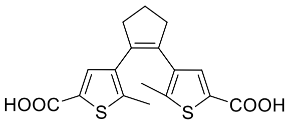	1.4	-	14
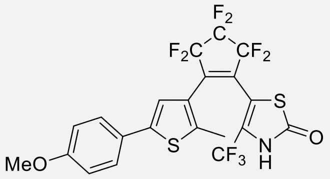	-	2.8 [d]	15
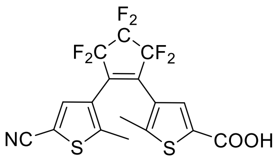	1.2	-	16
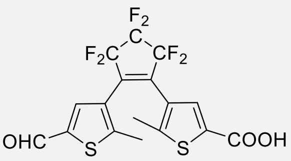	0.8	-	16
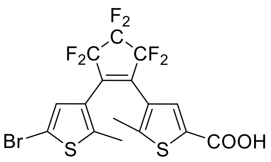	0.7	-	16
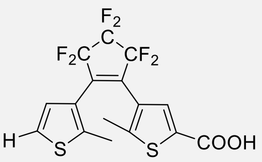	0.4	-	16

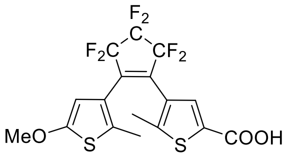	0.4	-	16
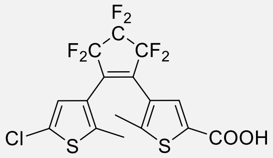	0.3	-	16
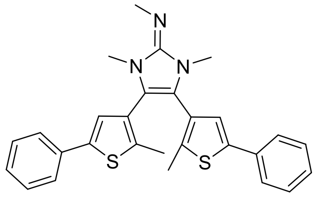	-6.1 [e]	-	17
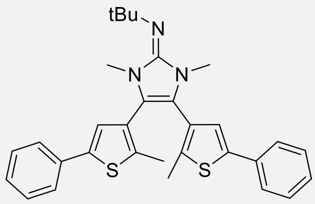	-8.7 [e]	-	17
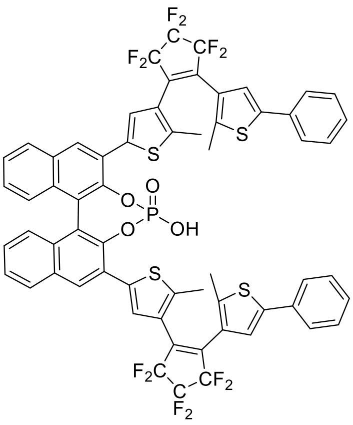	-1.1 [f]	-	18

[a] pK_a^c - pK_a^o in acetonitrile. [b] pK_a^c - pK_a^o in water or water:organic solvent mixtures. For **DAE1-9**, $\Delta pK_{a,H2O}$ in pure water were estimated from $\Delta pK_{a,ACN}$ using equation S2. [c] In methanol:water 5:2. [d] In acetonitrile:water 3:7. [e] Computed at the DFT level in acetonitrile solvent. [f] Computed at the DFT level in DMSO solvent.

5. NMR and IR Spectra of Selected Compounds

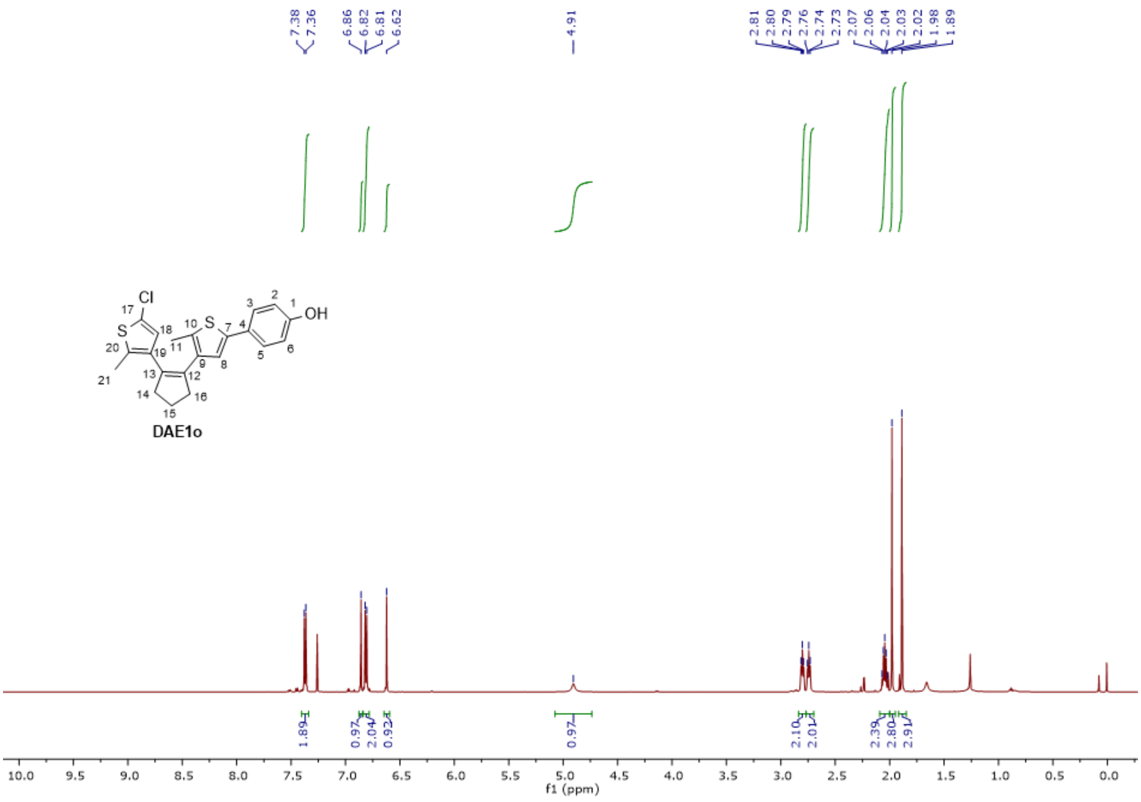

^{1}H NMR (600 MHz, $CDCl_3$)

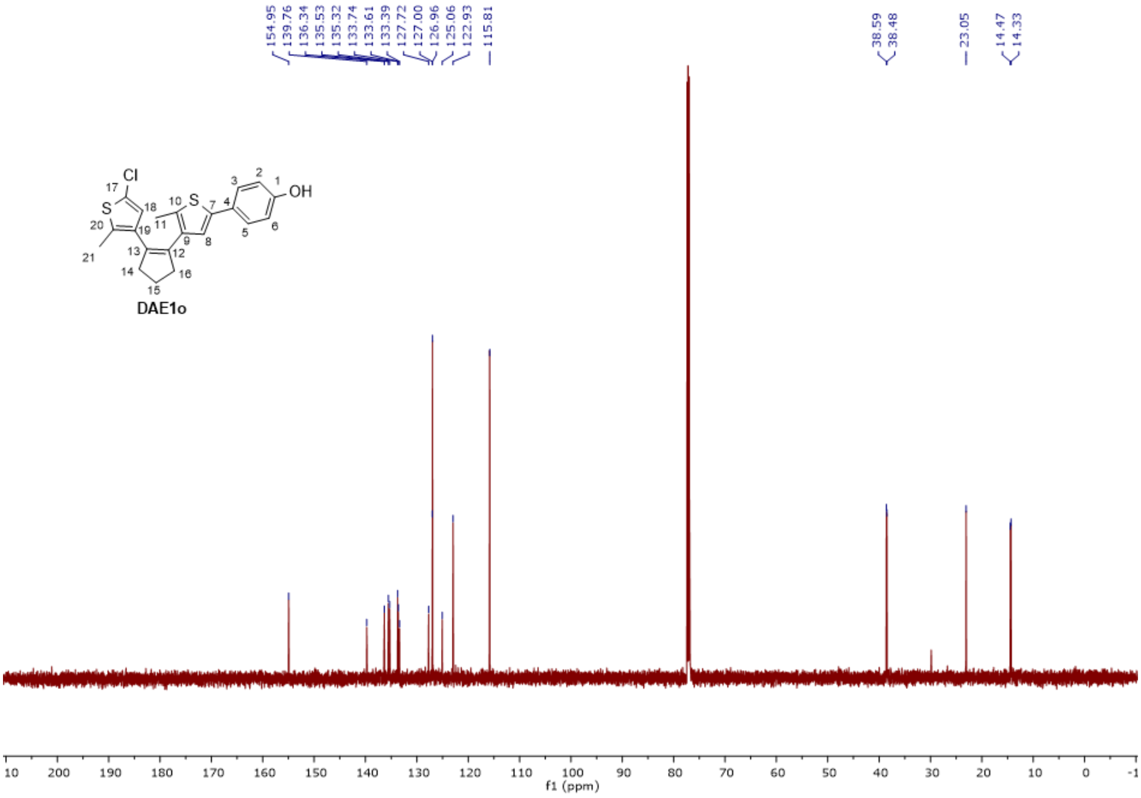

$^{13}C\{^{1}H\}$ NMR (151 MHz, $CDCl_3$)

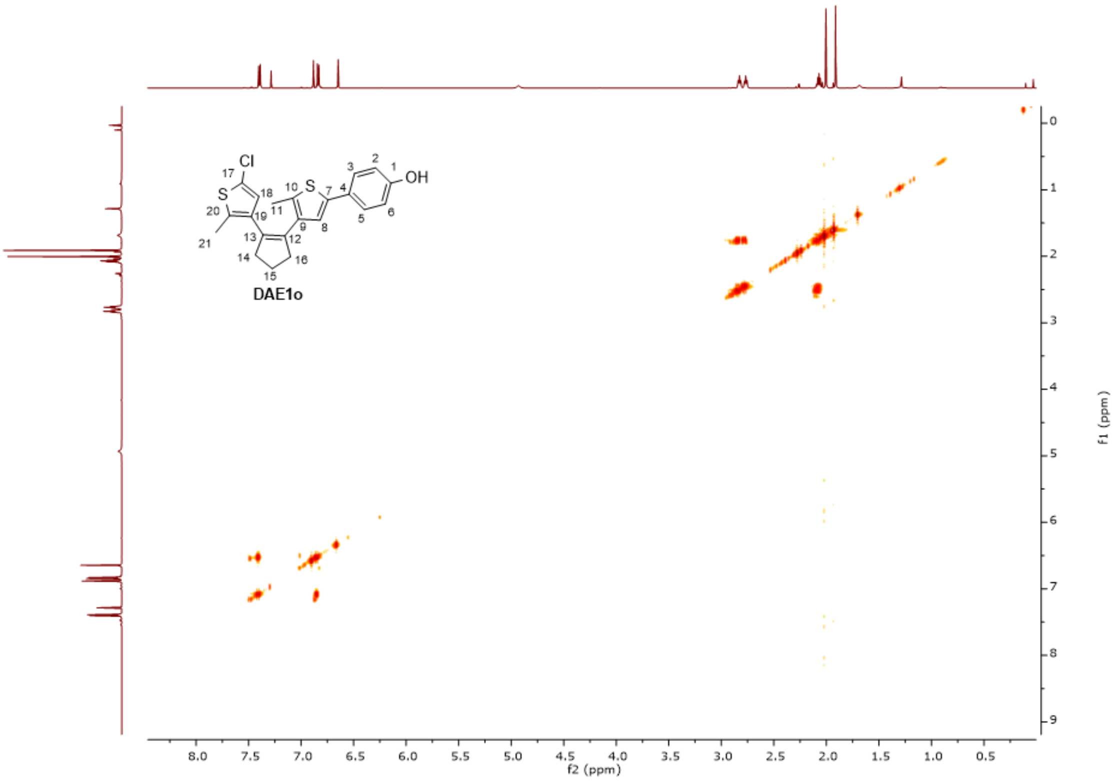

^{1}H-^{1}H COSY NMR (600 MHz, $CDCl_3$)

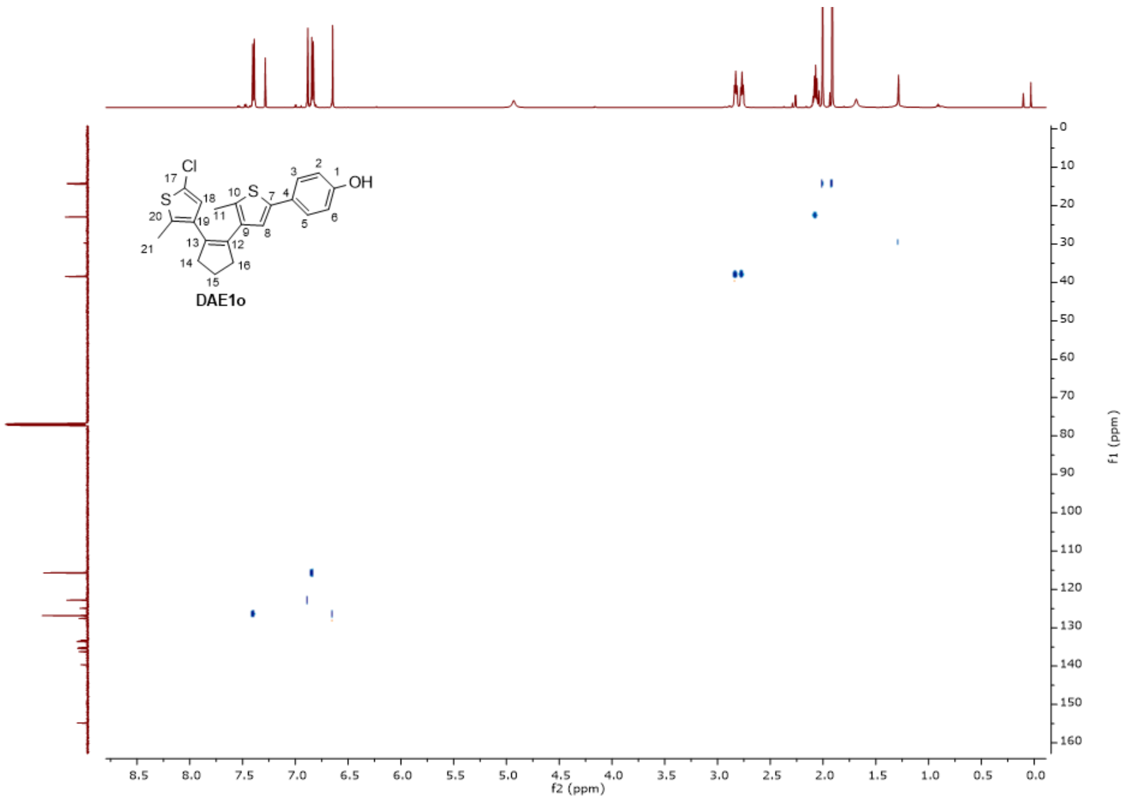

^{1}H-^{13}C HSQC NMR (600 MHz, $CDCl_3$)

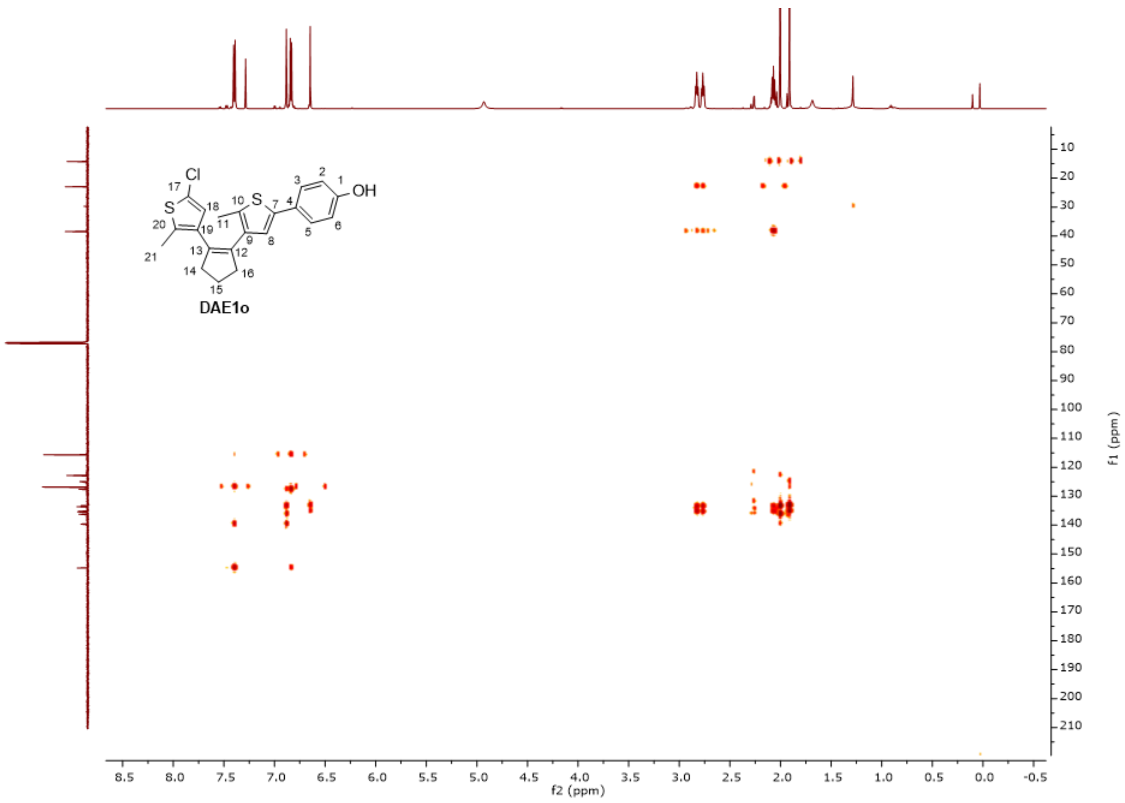

^{1}H-^{13}C HMBC NMR (600 MHz, CDCl$_3$)

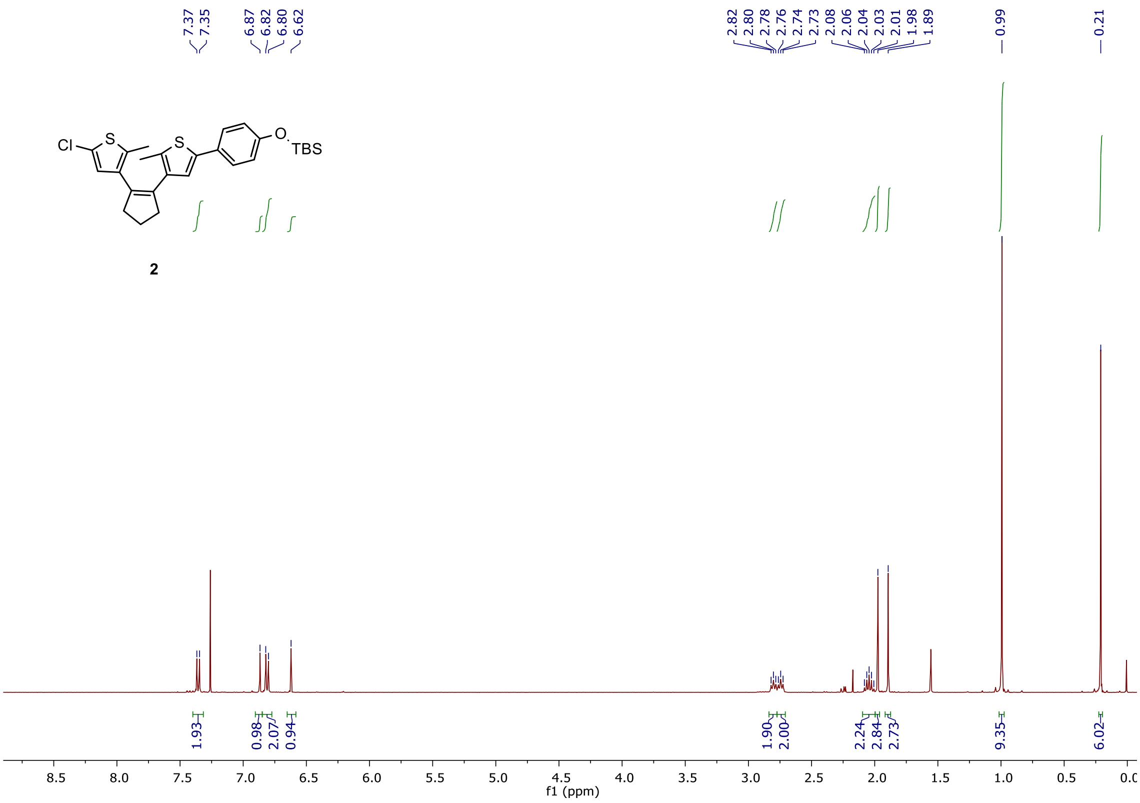

^{1}H NMR (400 MHz, CDCl$_3$)

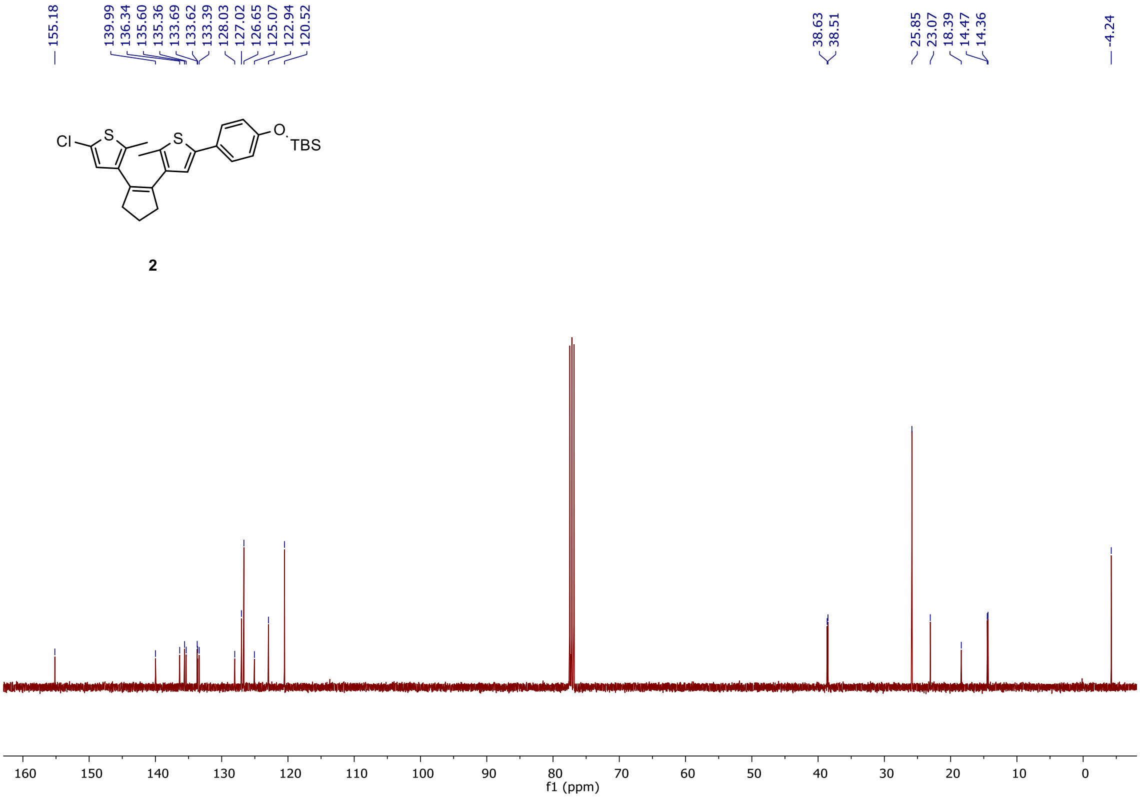

$^{13}C\{^{1}H\}$ NMR (101 MHz, CDCl$_3$)

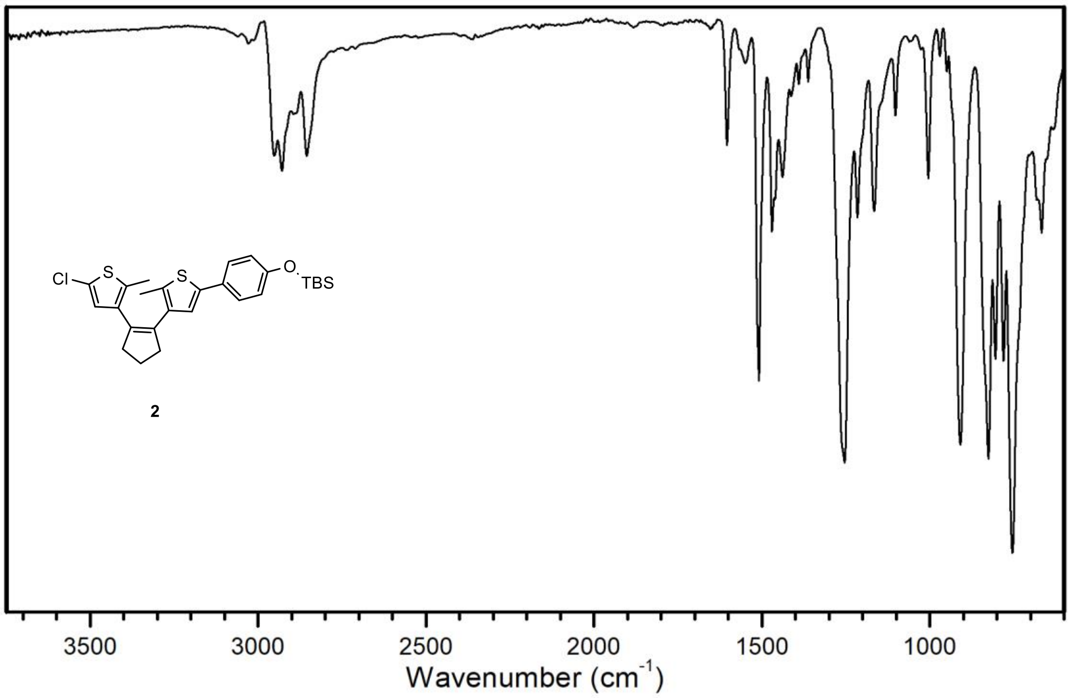

IR (ATR cm^{-1})

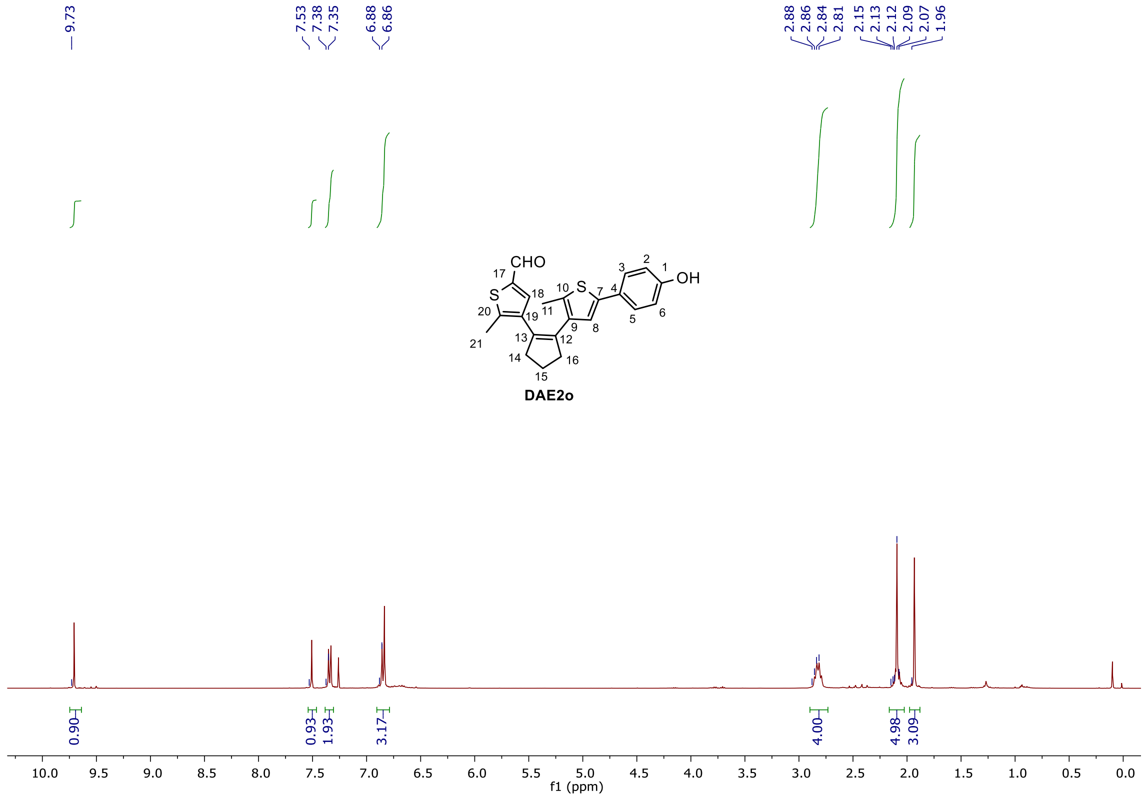

^{1}H NMR (400 MHz, $CDCl_3$)

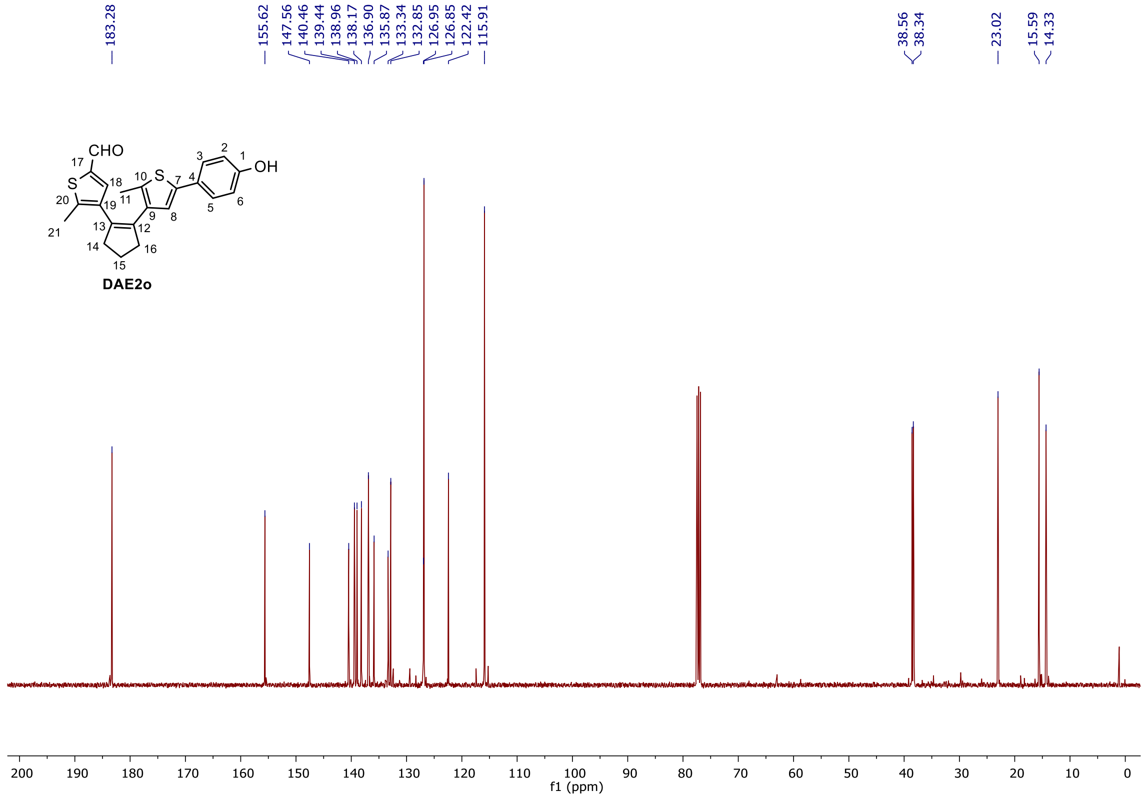

^{13}C{^{1}H} NMR (101 MHz, $CDCl_3$)

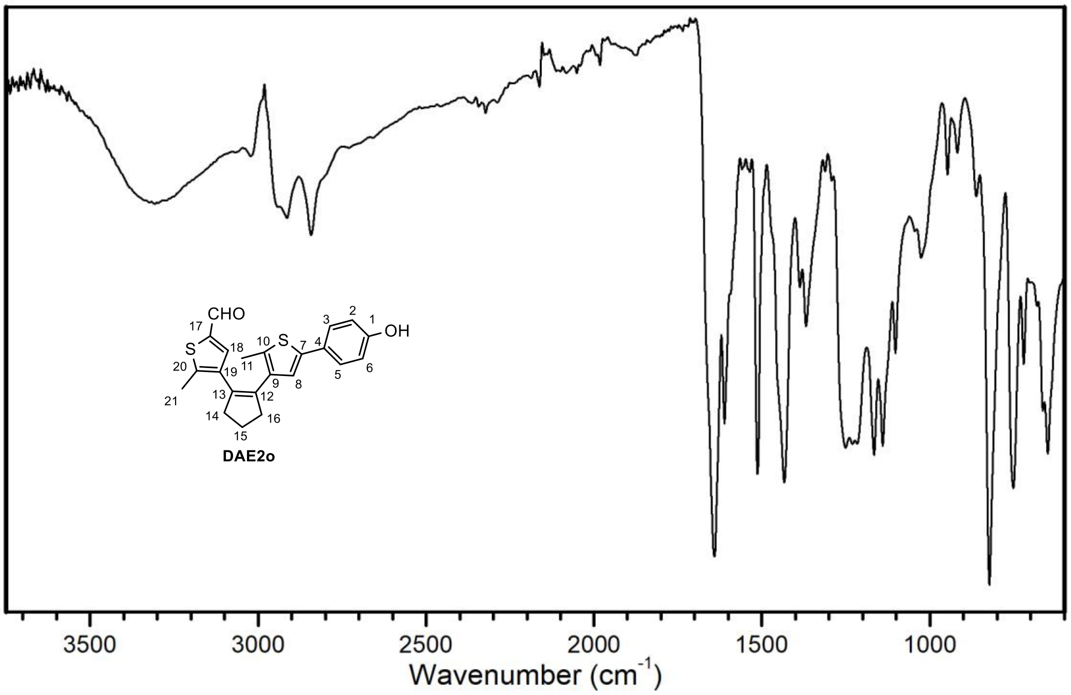

IR (ATR cm^{-1})

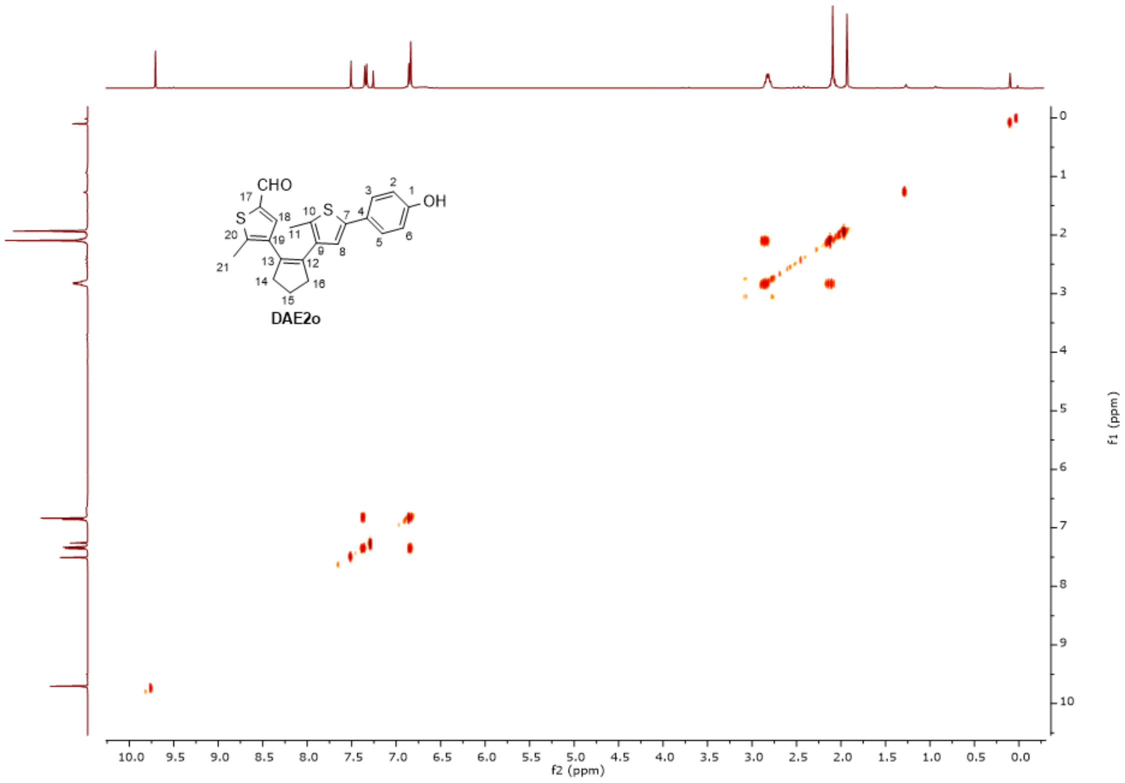

^1H-^1H COSY NMR (400 MHz, $CDCl_3$)

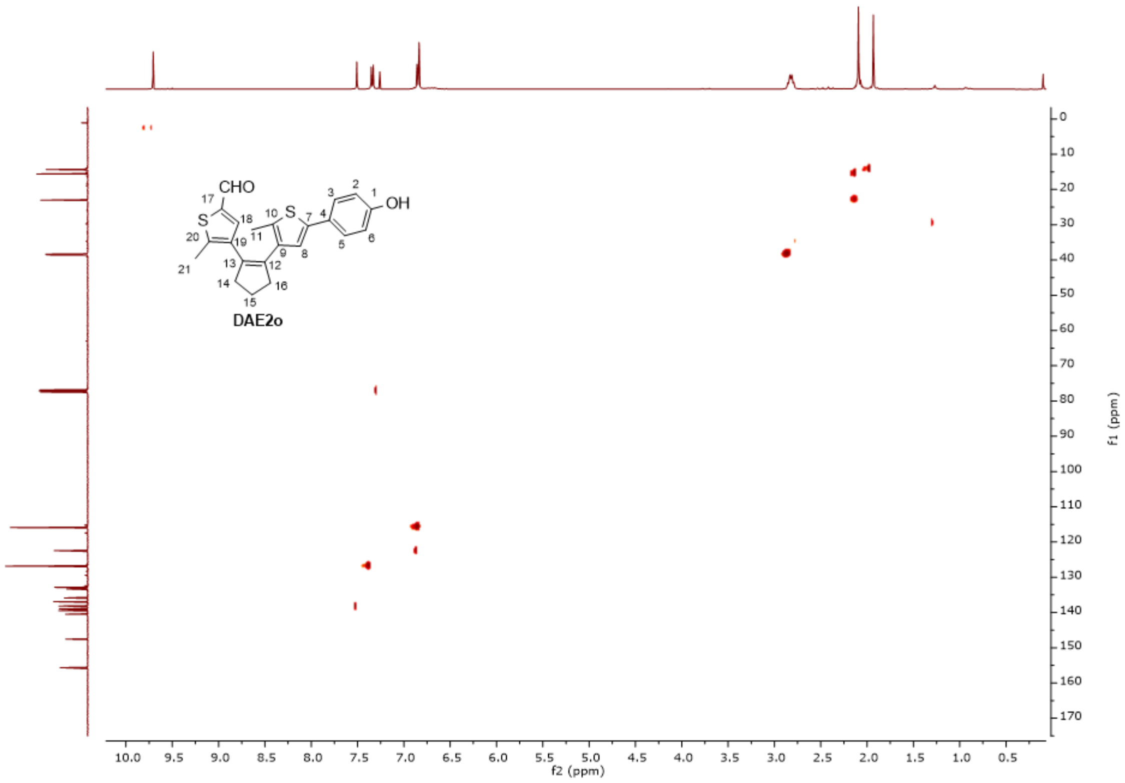

^{1}H-^{13}C HSQC NMR (400 MHz, $CDCl_3$)

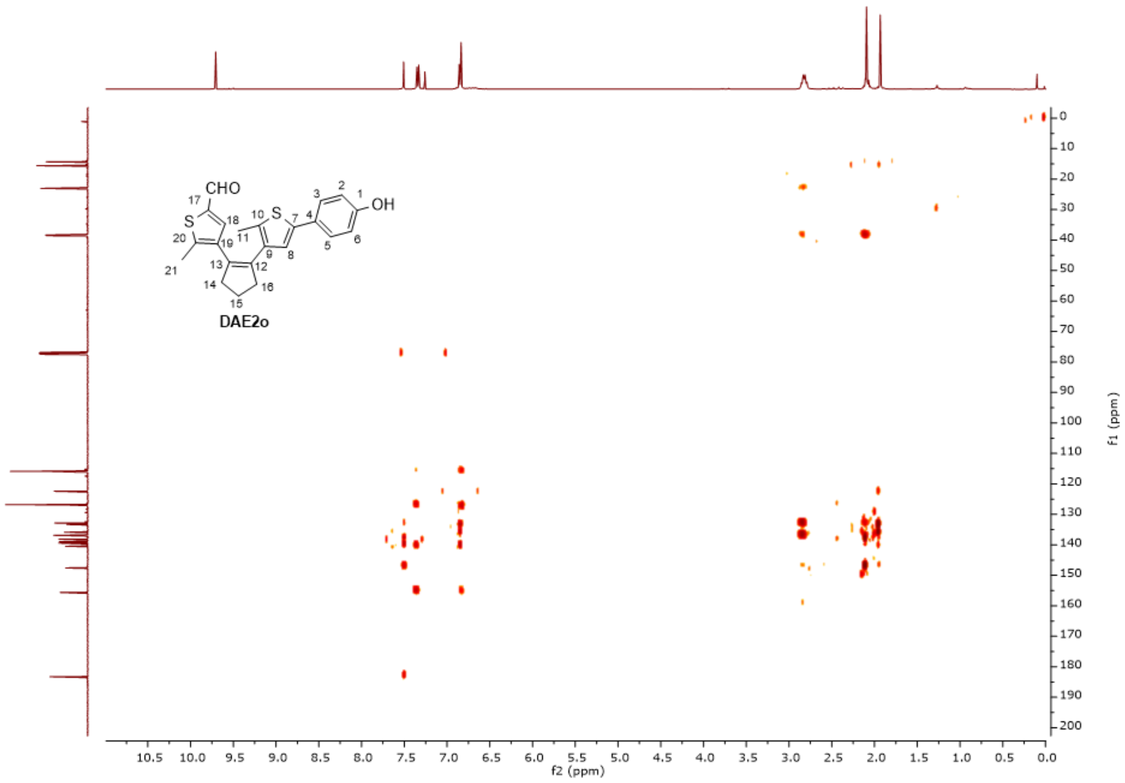

^{1}H-^{13}C HMBC NMR (400 MHz, $CDCl_3$)

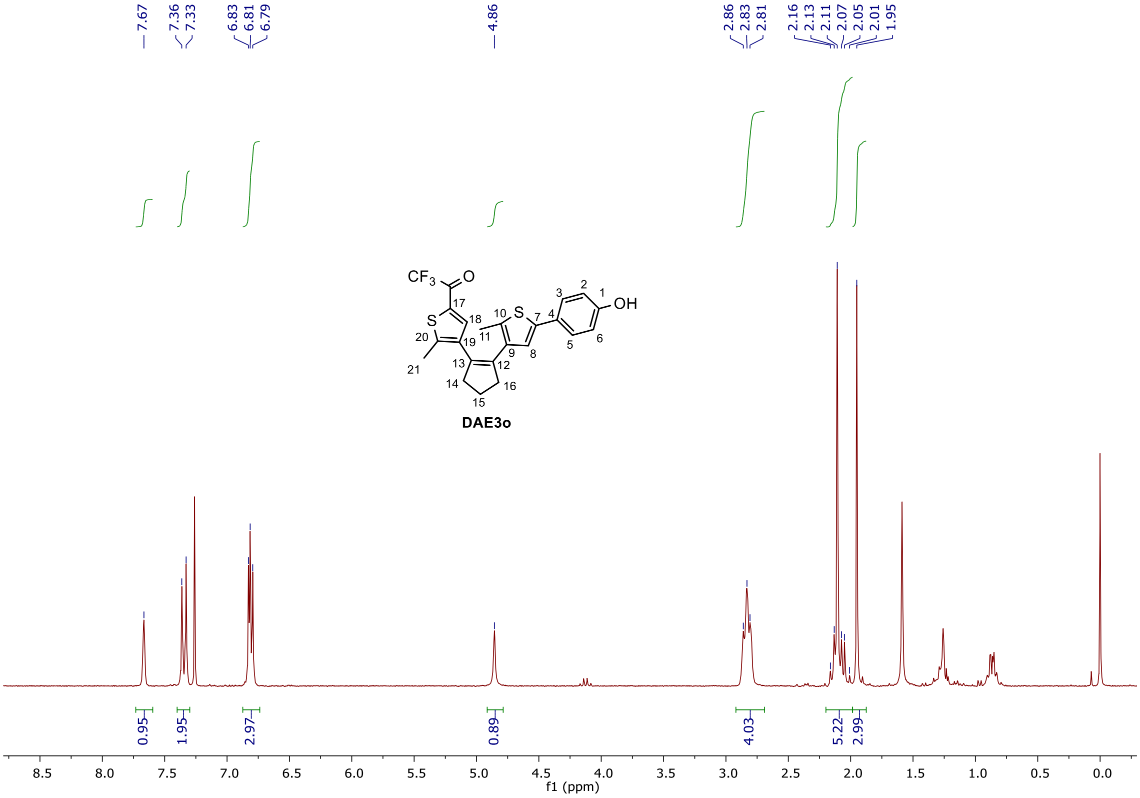

^{1}H NMR (250 MHz, $CDCl_3$)

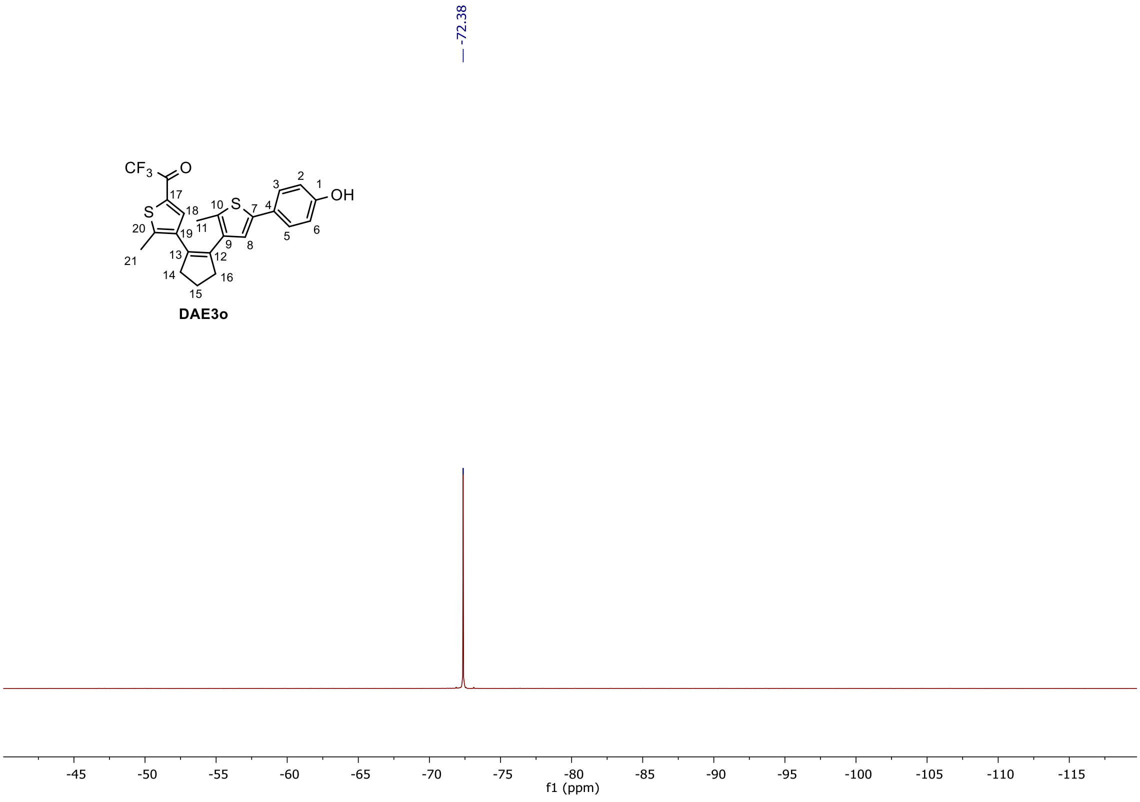

^{19}F NMR (235 MHz, $CDCl_3$)

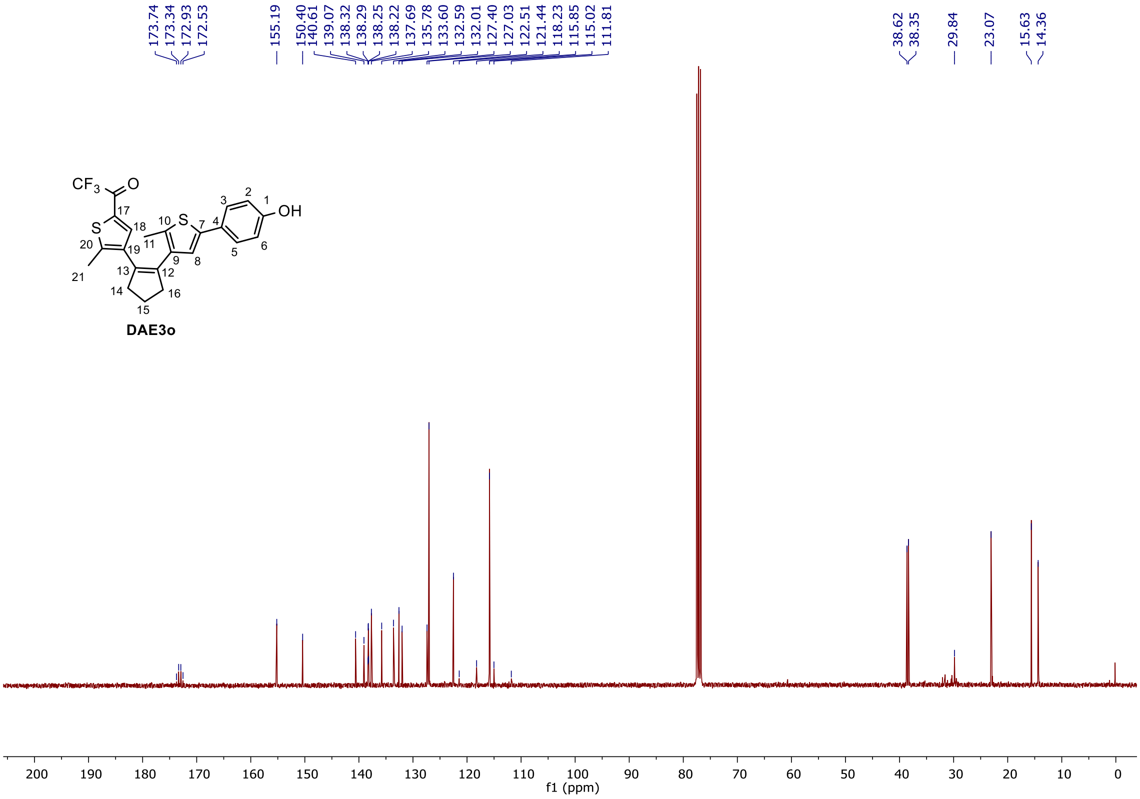

$^{13}C\{^1H\}$ NMR (91 MHz, $CDCl_3$)

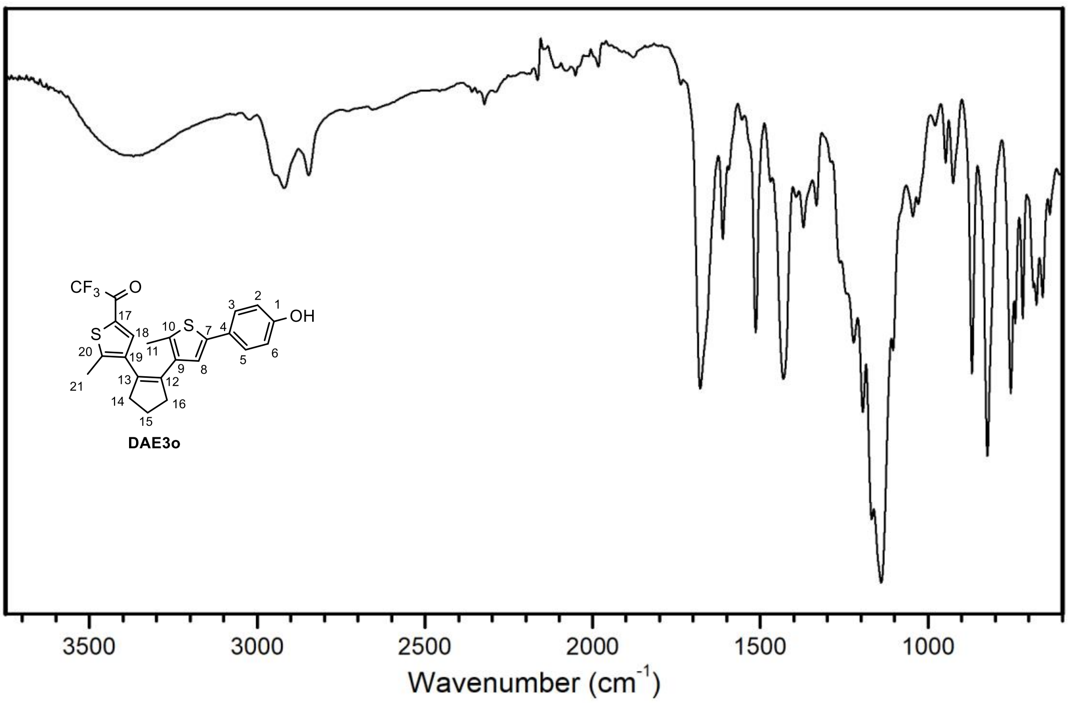

IR (ATR cm^{-1})

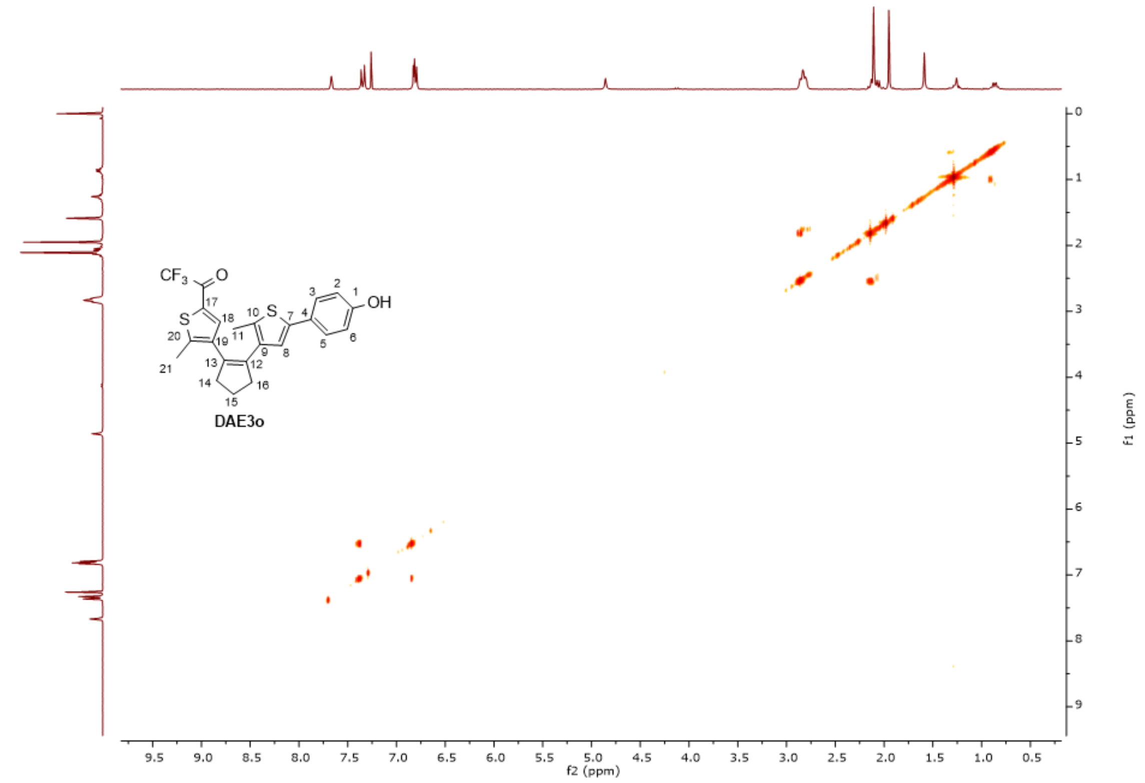

^{1}H-^{1}H COSY NMR (600 MHz, $CDCl_3$)

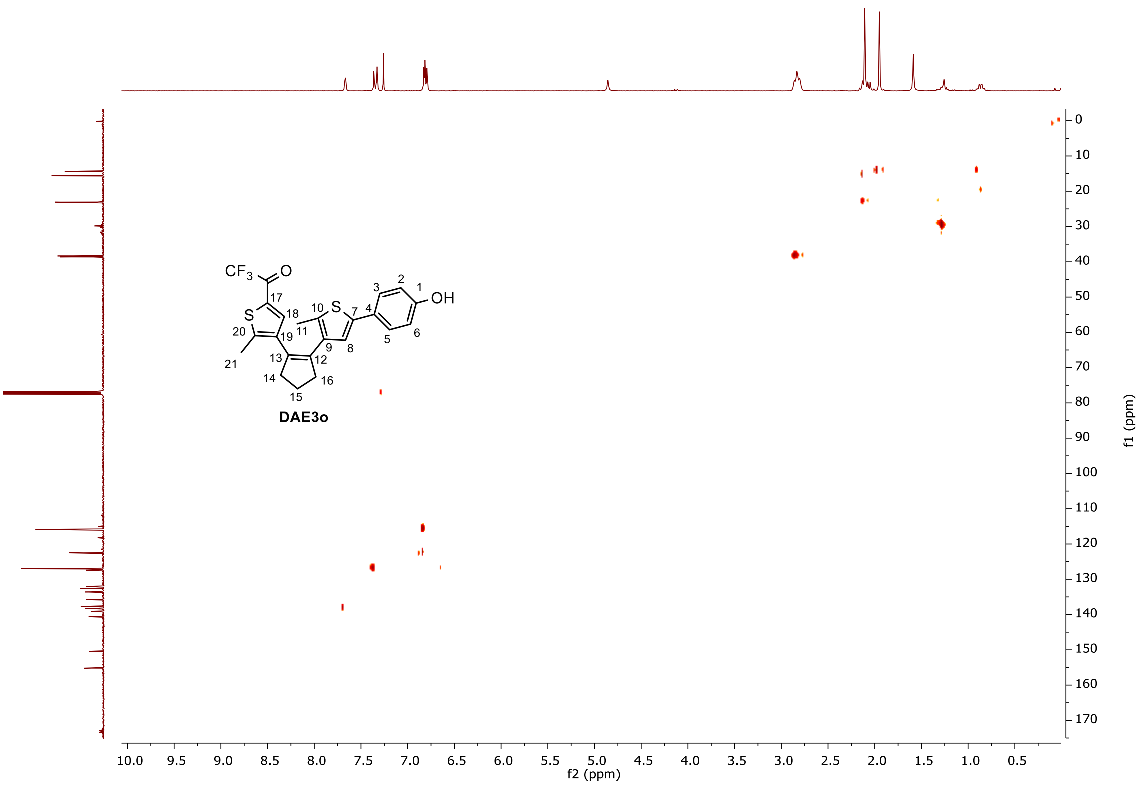

^{1}H-^{13}C HSQC NMR (600 MHz, $CDCl_3$)

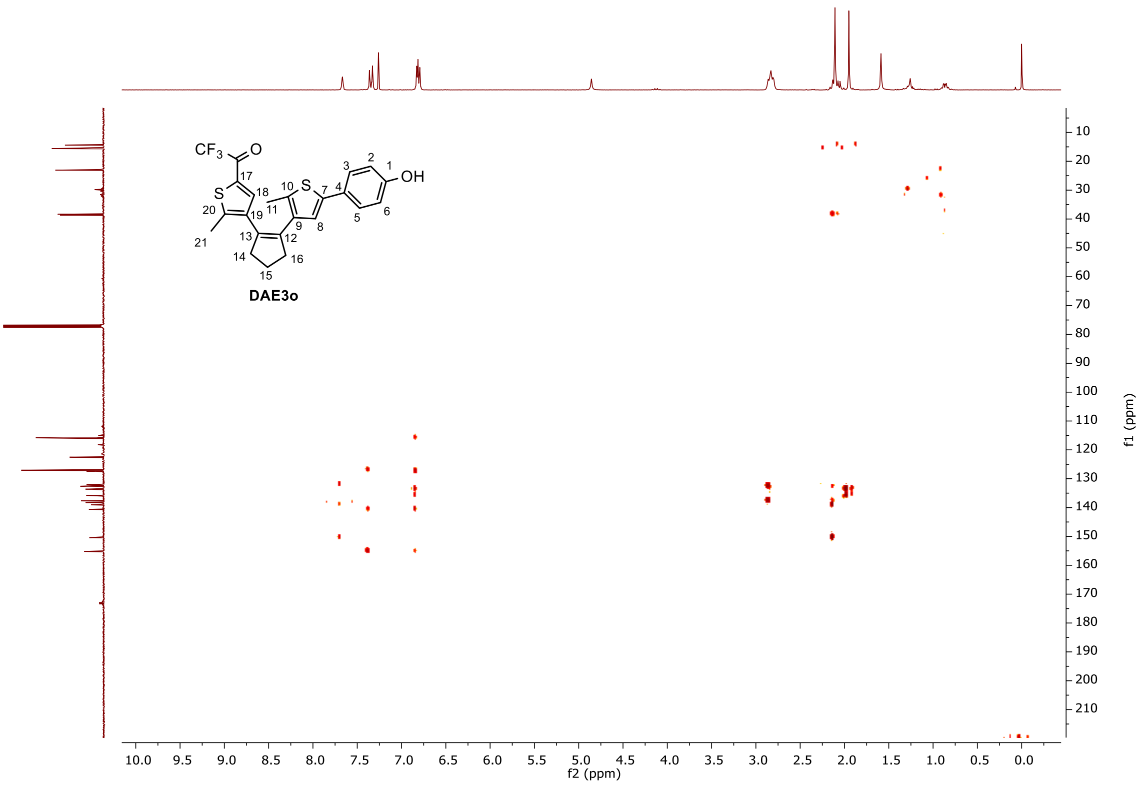

^{1}H-^{13}C HMBC NMR (600 MHz, CDCl$_3$)

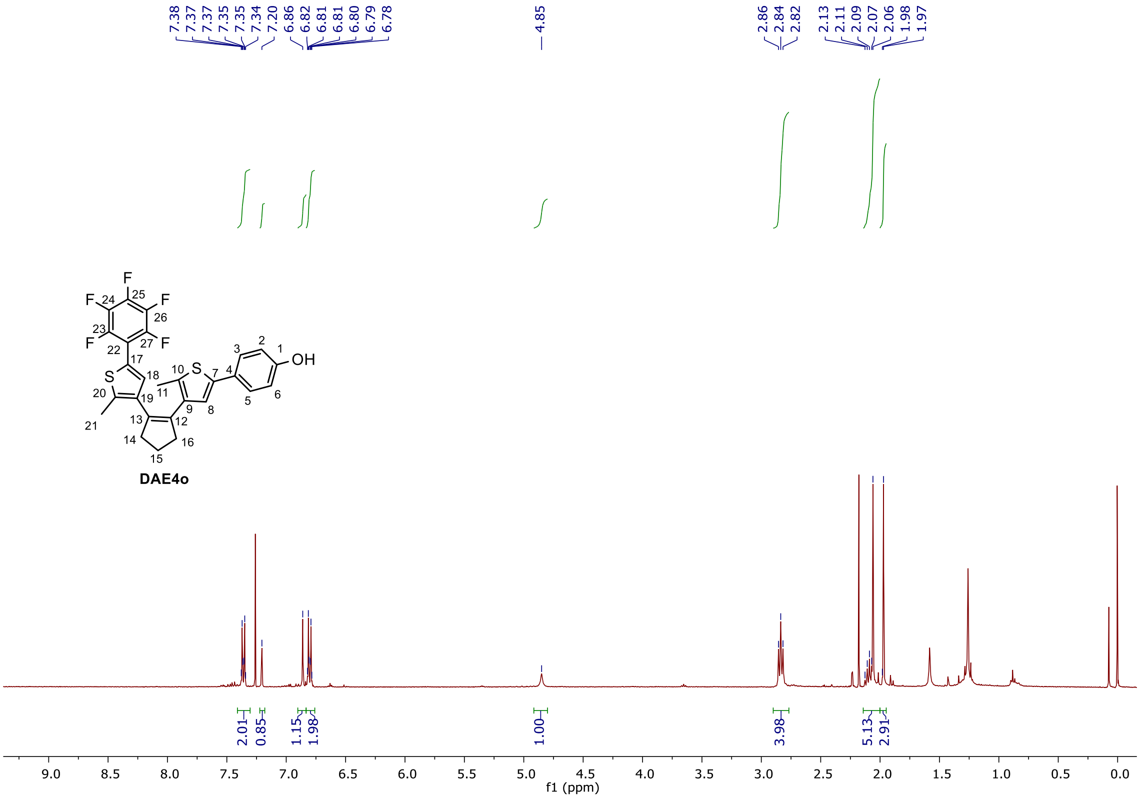

^{1}H NMR (600 MHz, CDCl$_3$)

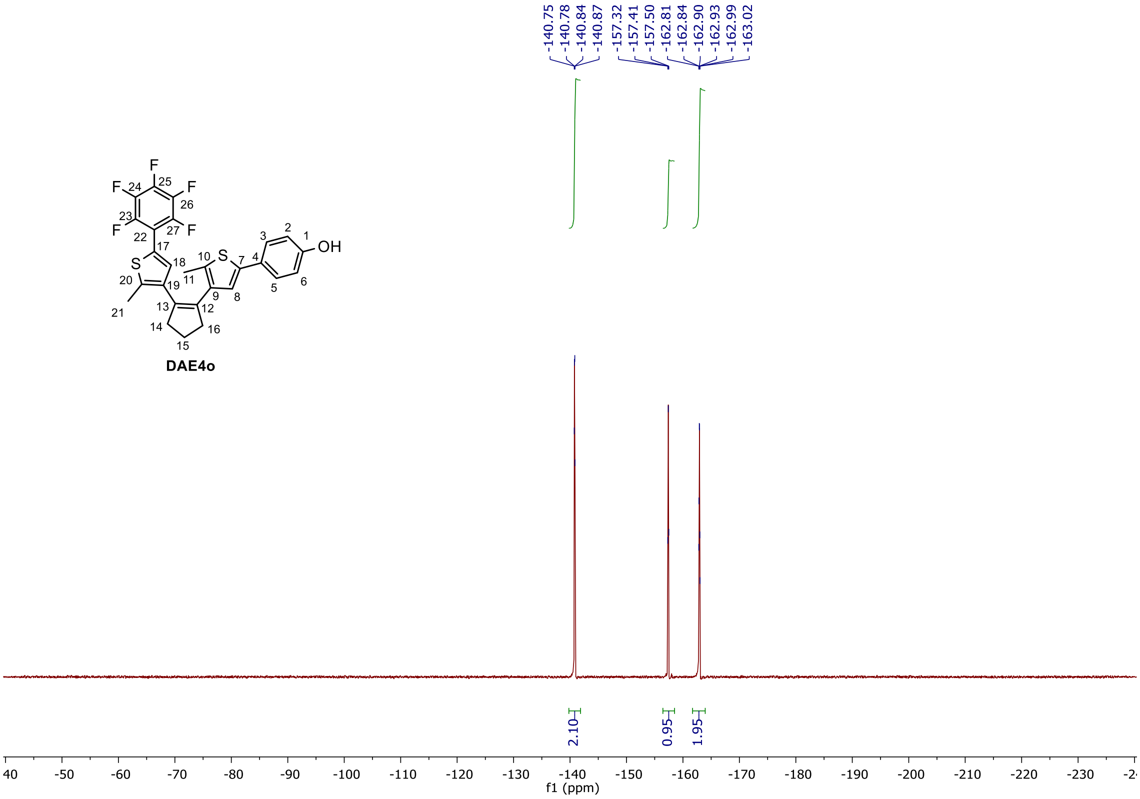

^{19}F NMR (235 MHz, $CDCl_3$)

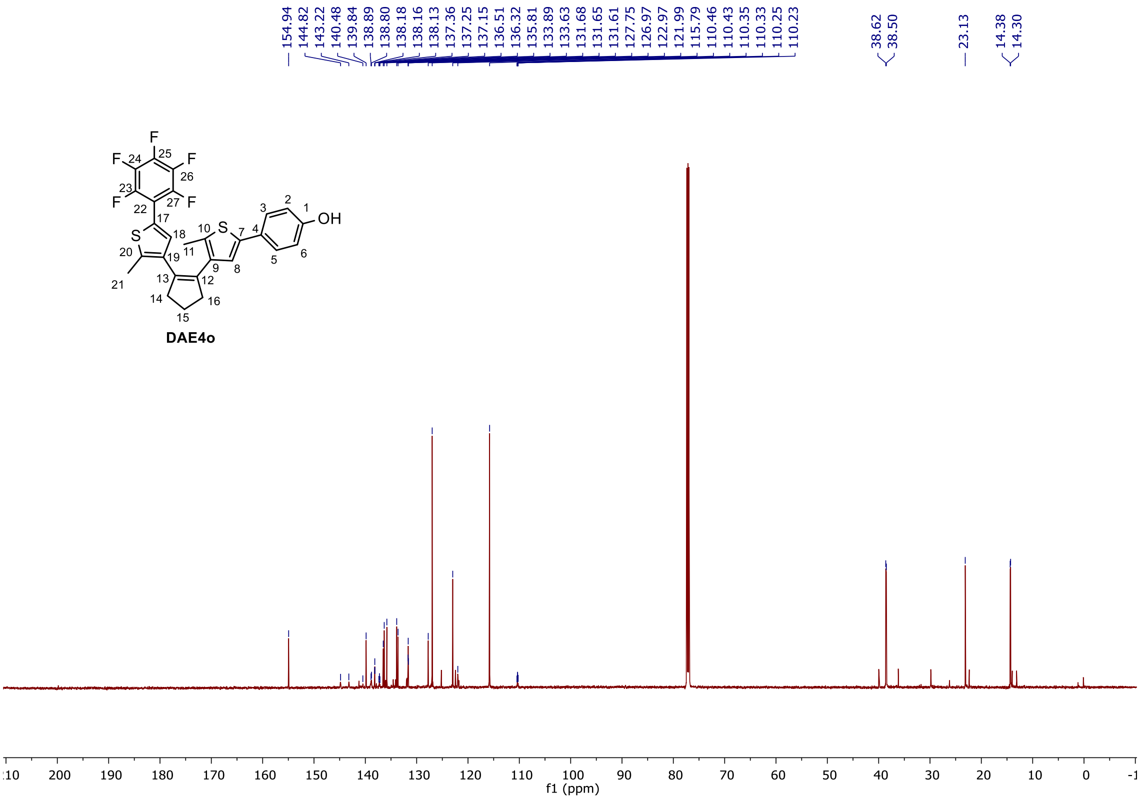

^{13}C{^{1}H} NMR (151 MHz, $CDCl_3$)

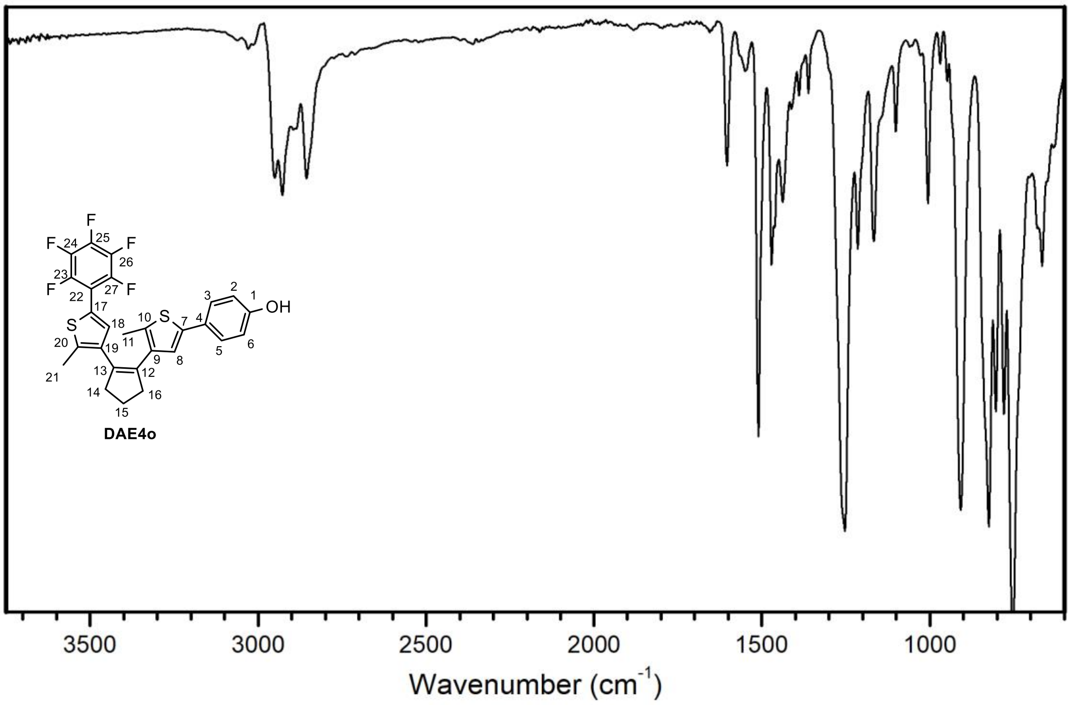

IR (ATR cm-1)

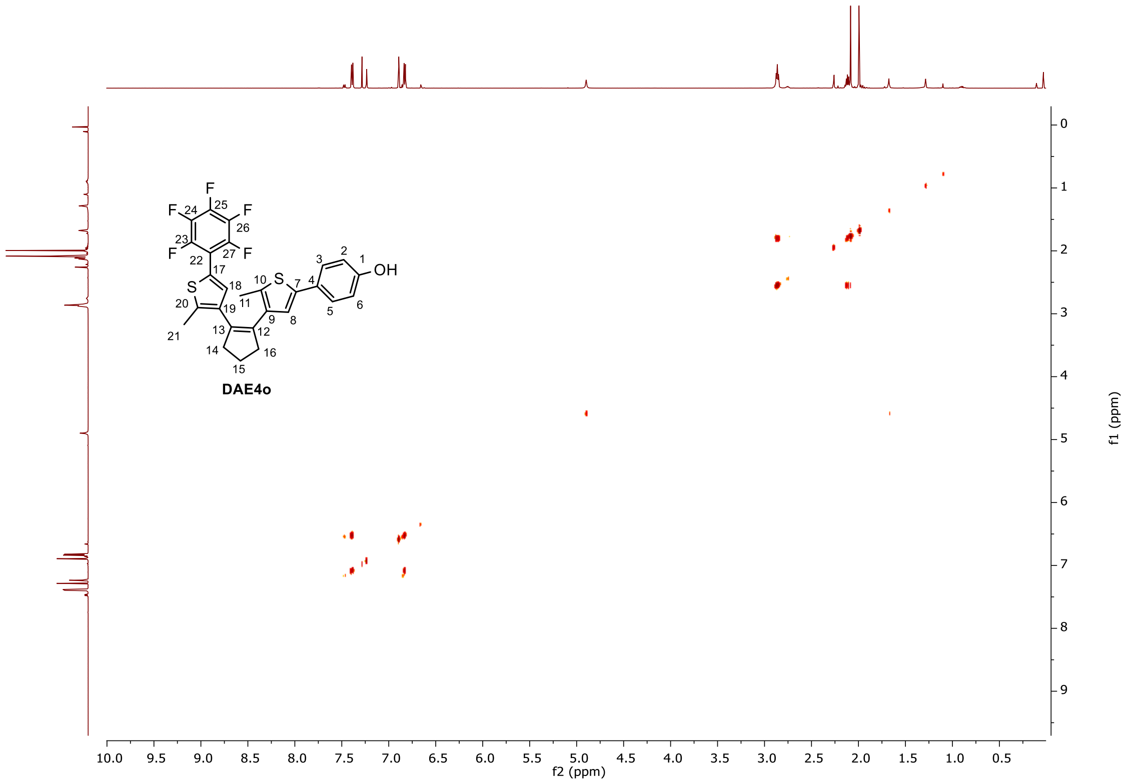

^{1}H-^{1}H COSY NMR (600 MHz, CDCl$_3$)

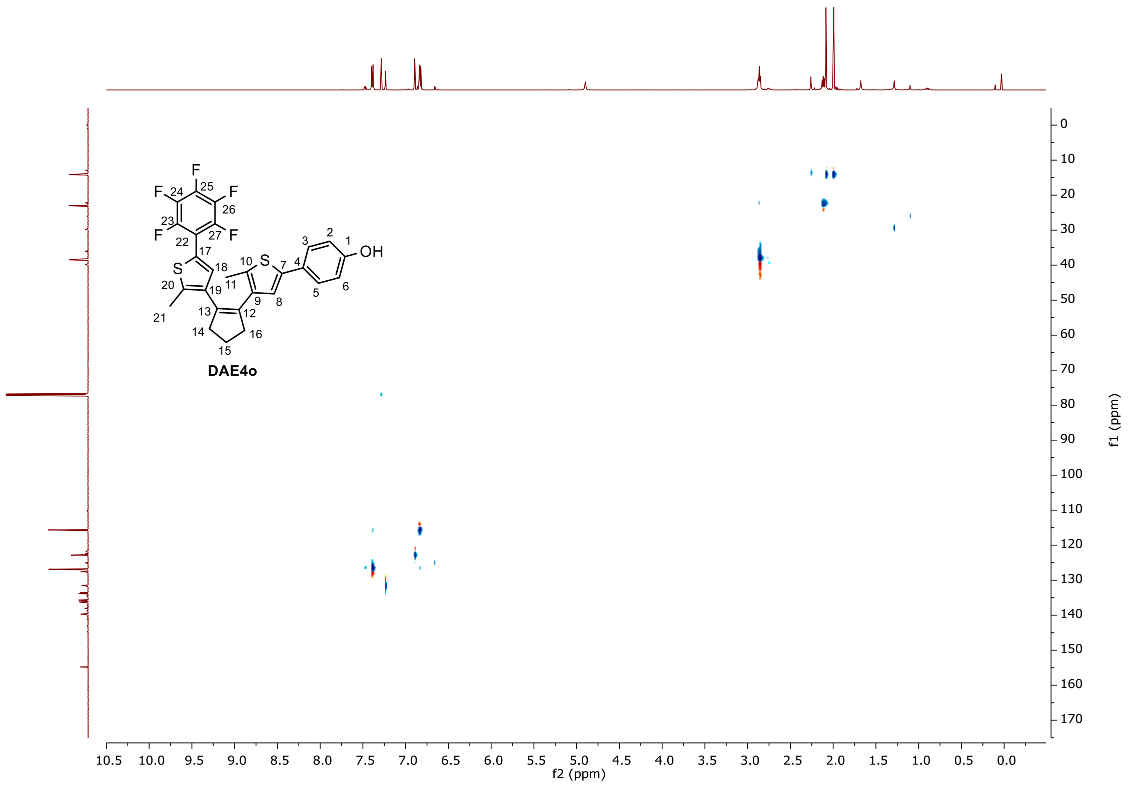

^{1}H-^{13}C HSQC NMR (600 MHz, $CDCl_3$)

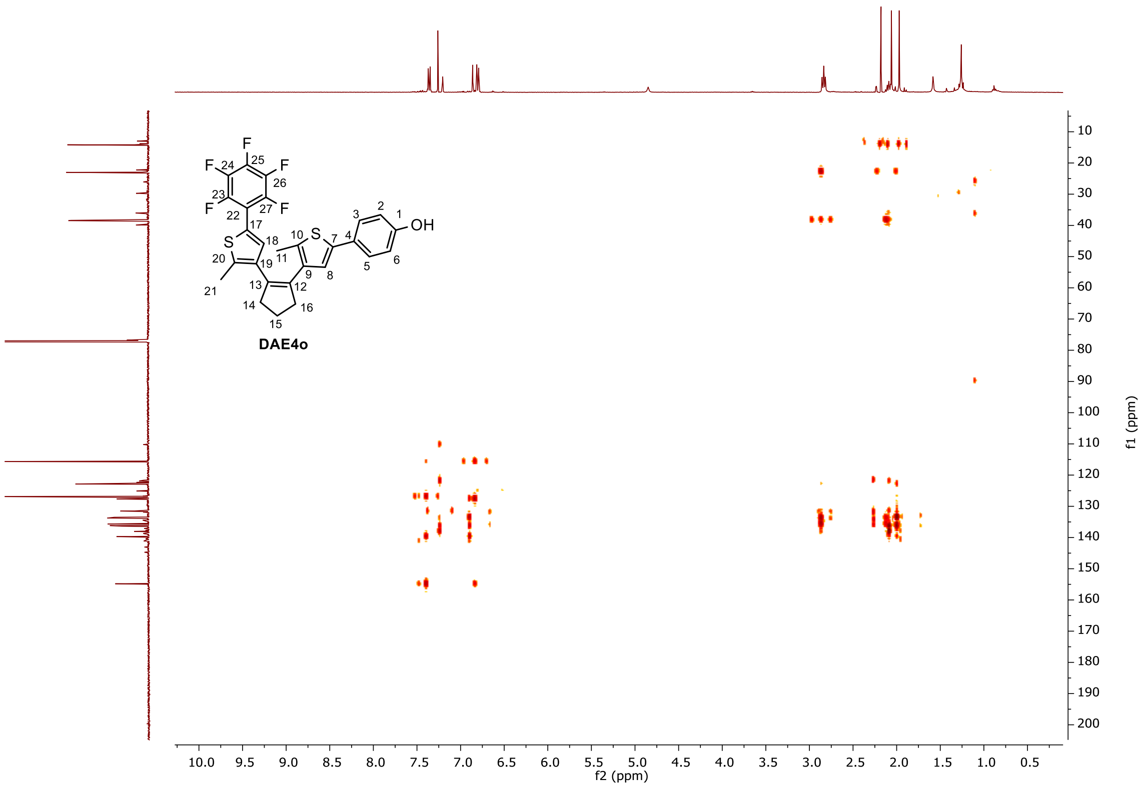

^{1}H-^{13}C HMBC NMR (600 MHz, $CDCl_3$)

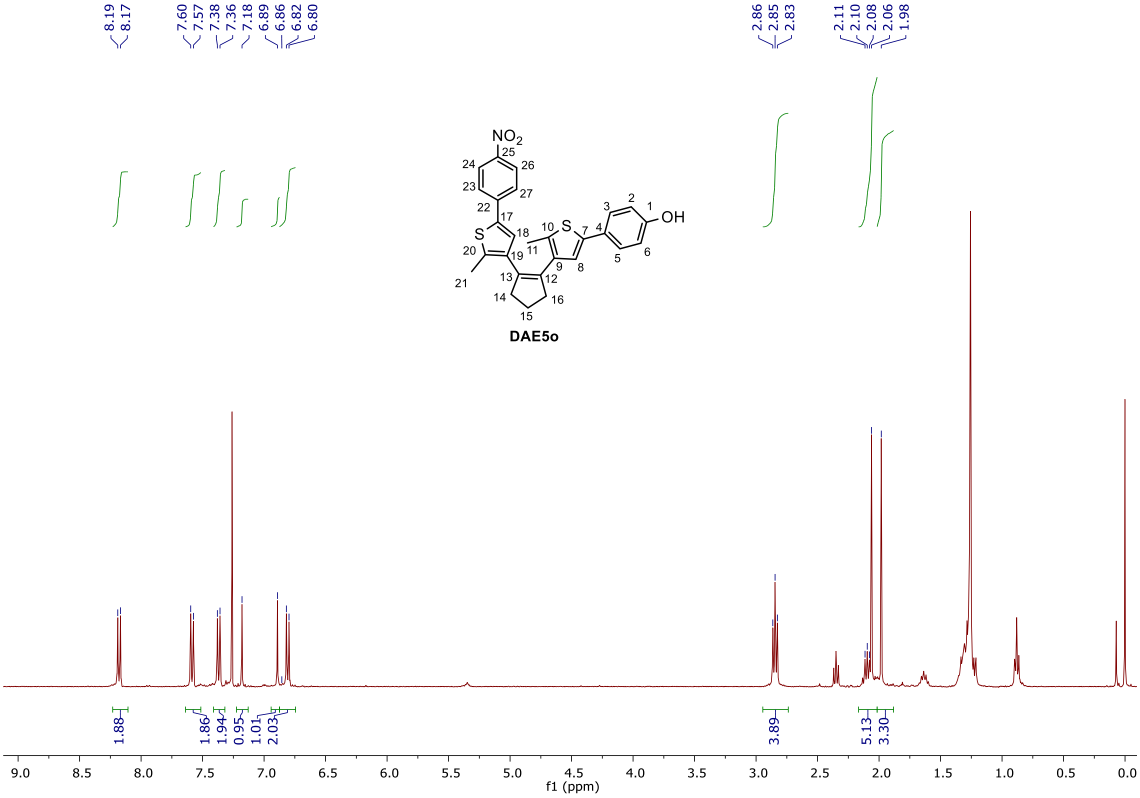

^{1}H NMR (400 MHz, $CDCl_3$)

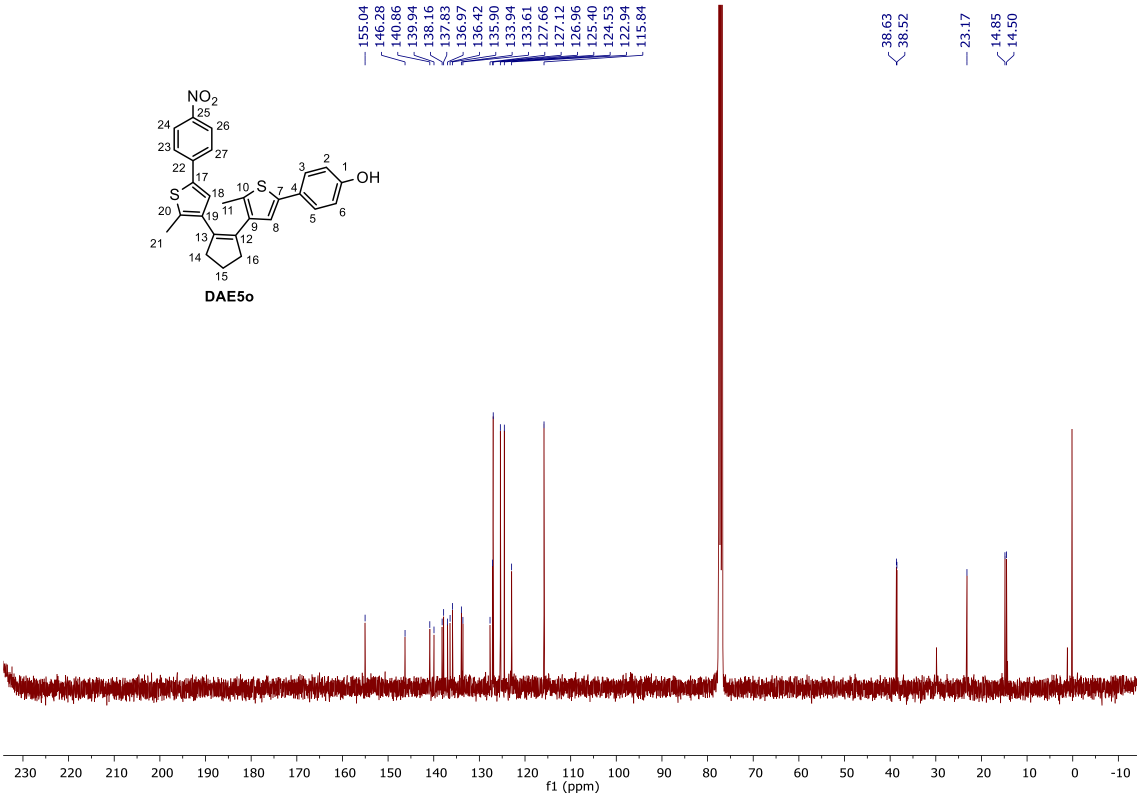

$^{13}C\{^{1}H\}$ NMR (151 MHz, $CDCl_3$)

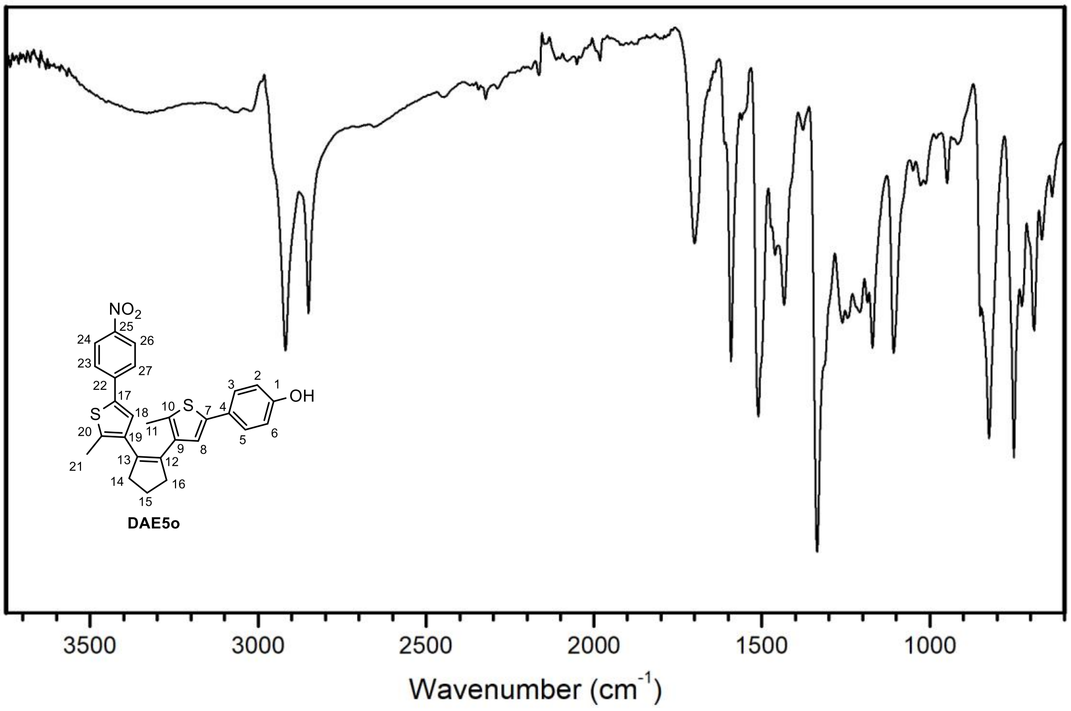

IR (ATR cm-1)

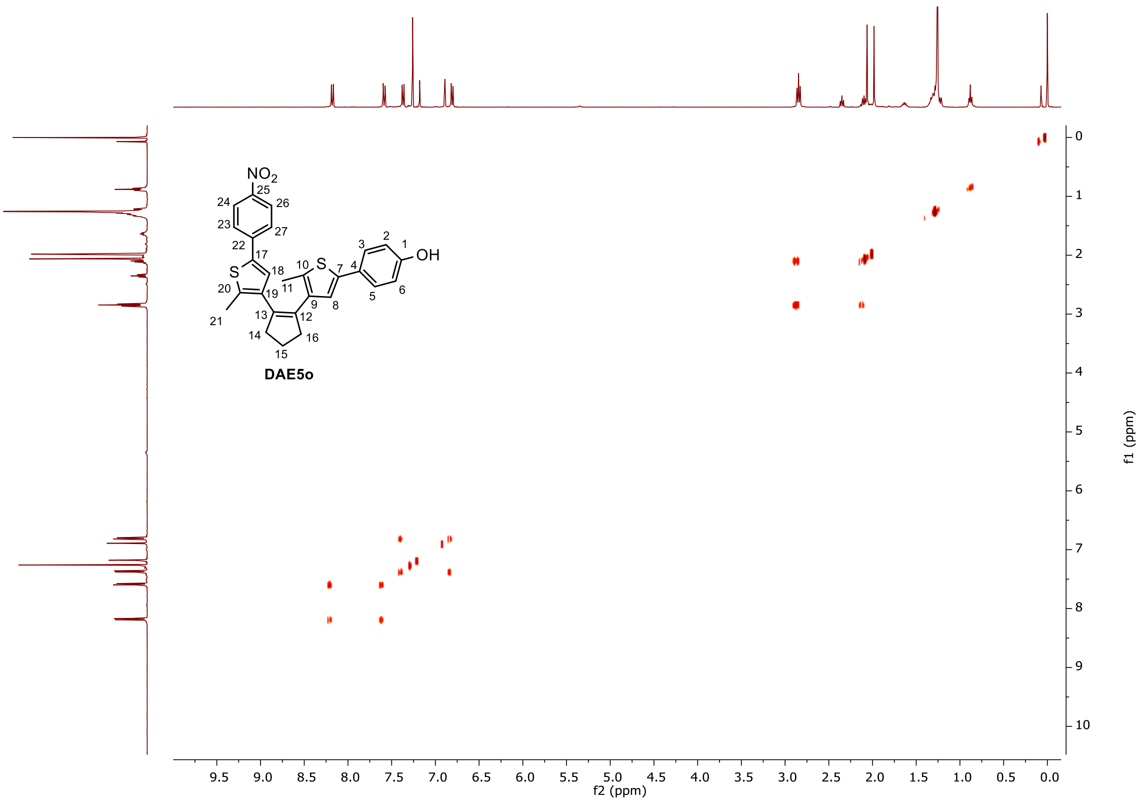

^{1}H-^{1}H COSY NMR (400 MHz, $CDCl_3$)

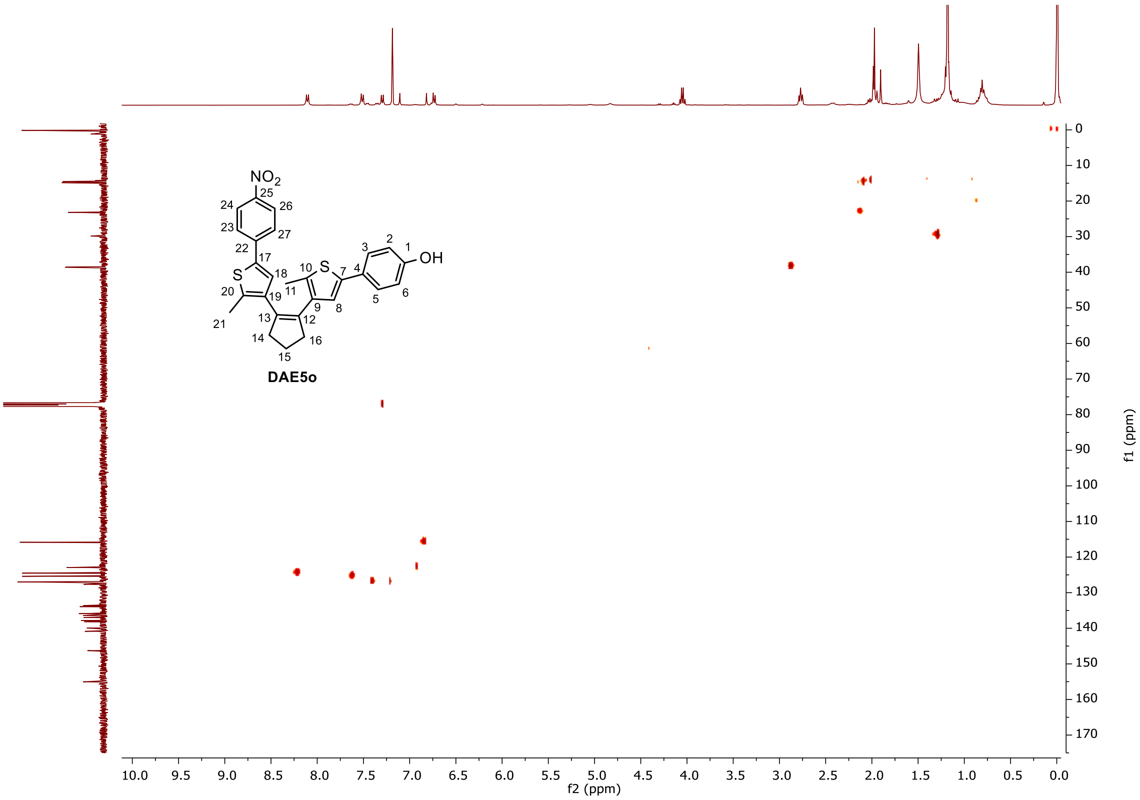

^{1}H-^{13}C HSQC NMR (400 MHz, CDCl$_3$)

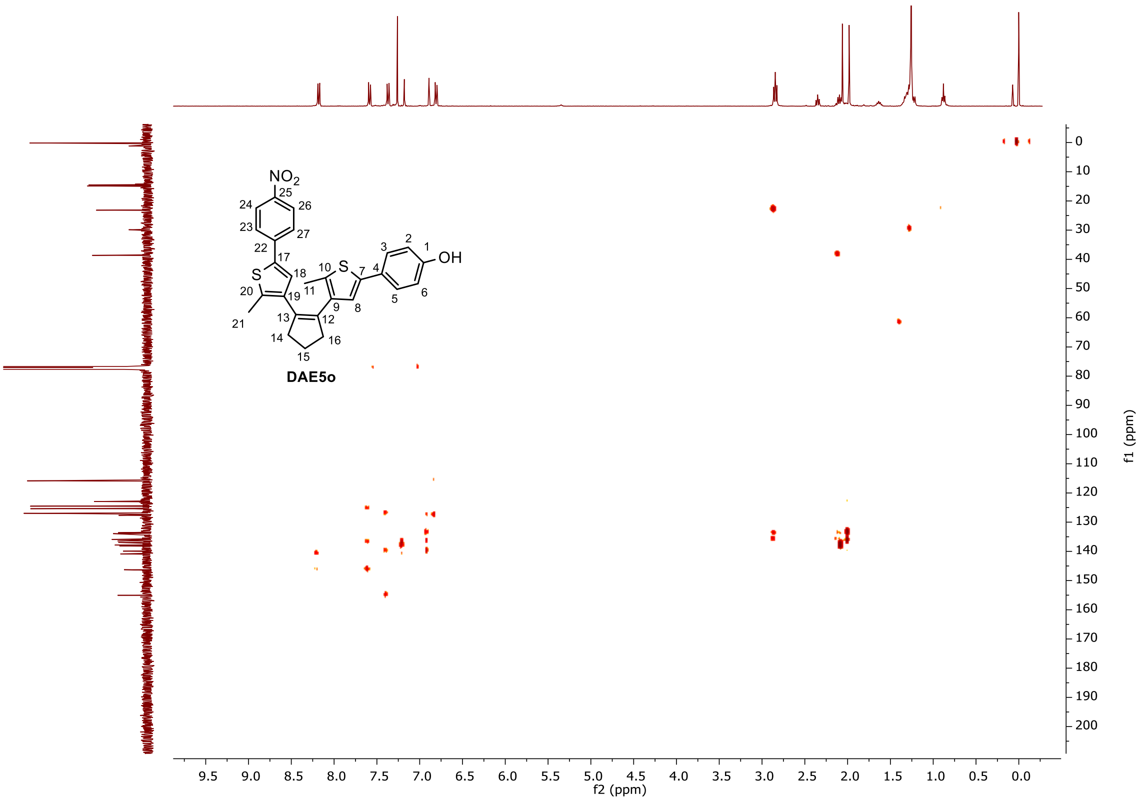

^{1}H-^{13}C HMBC NMR (400 MHz, CDCl$_3$)

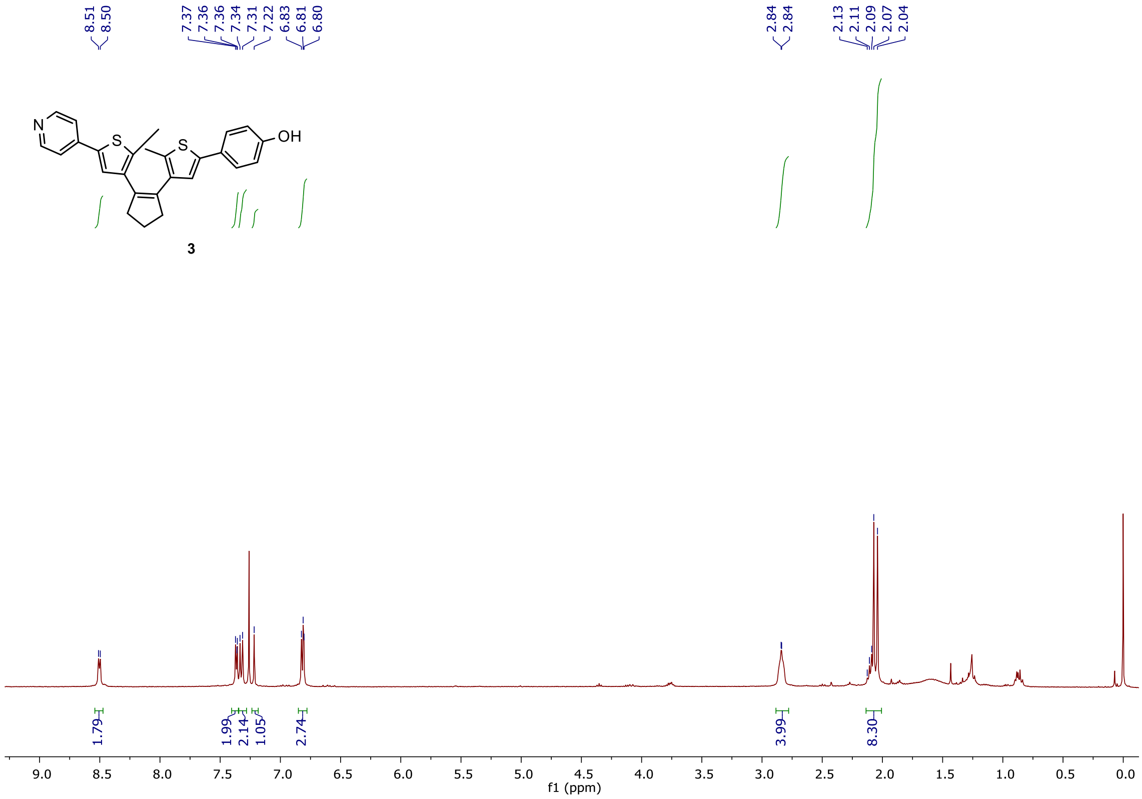

^{1}H NMR (400 MHz, $CDCl_3$)

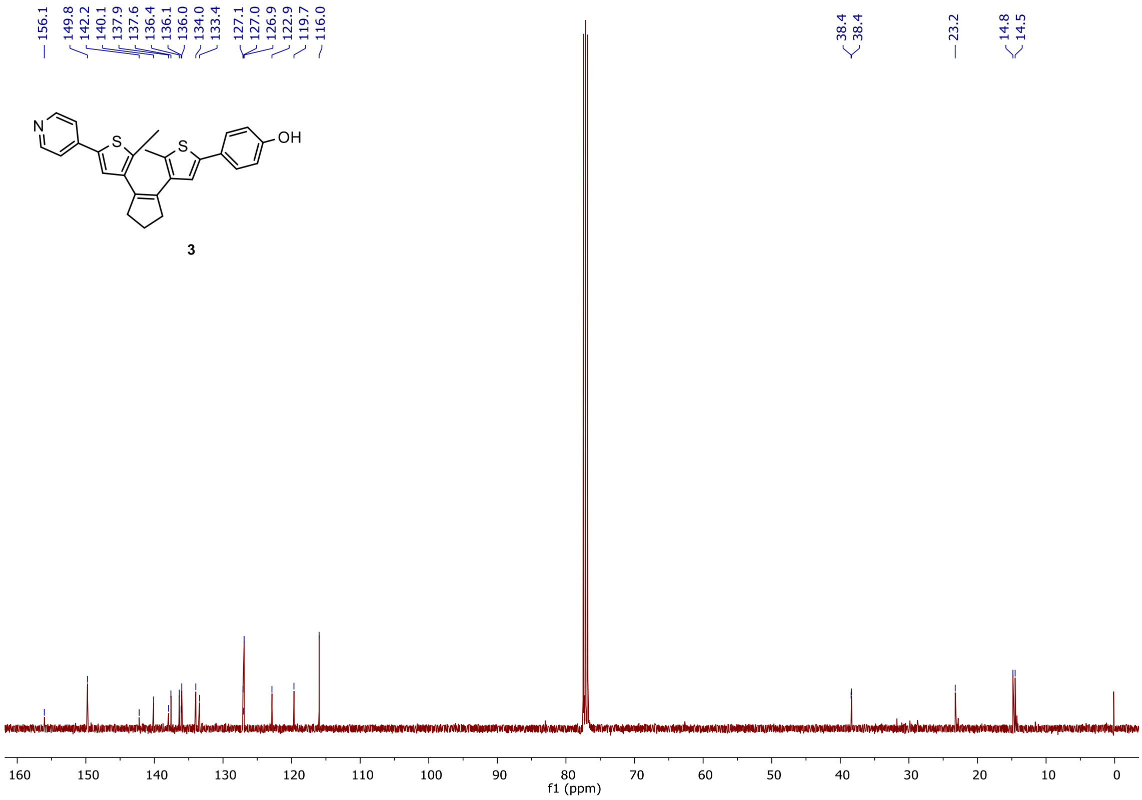

$^{13}C\{^{1}H\}$ NMR (101 MHz, $CDCl_3$)

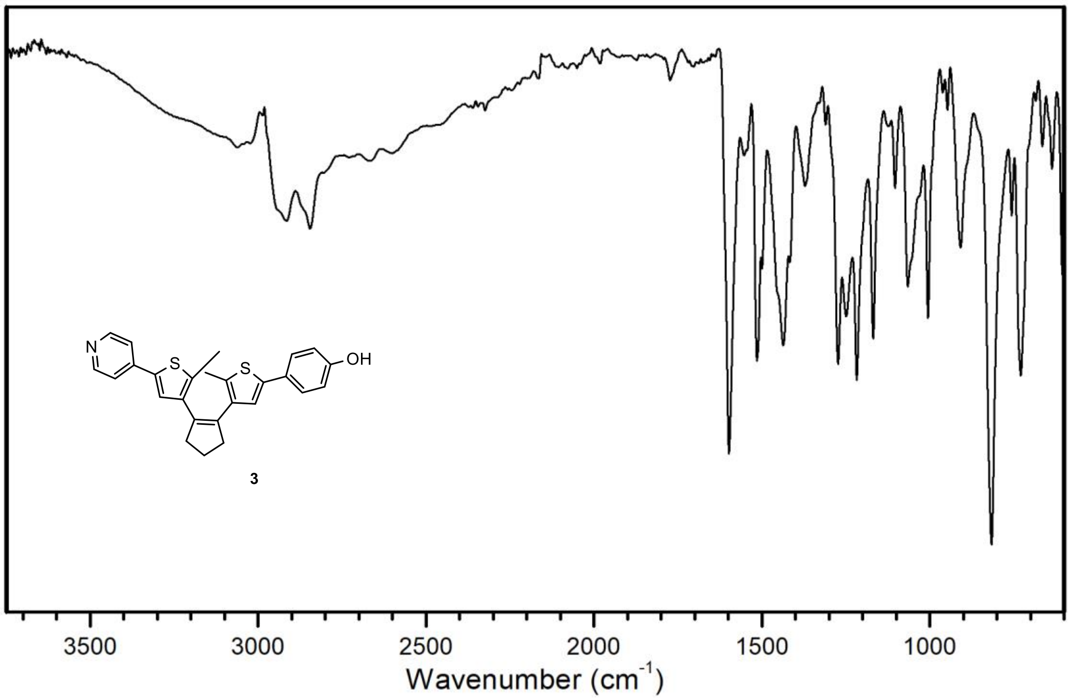

IR (ATR cm-1)

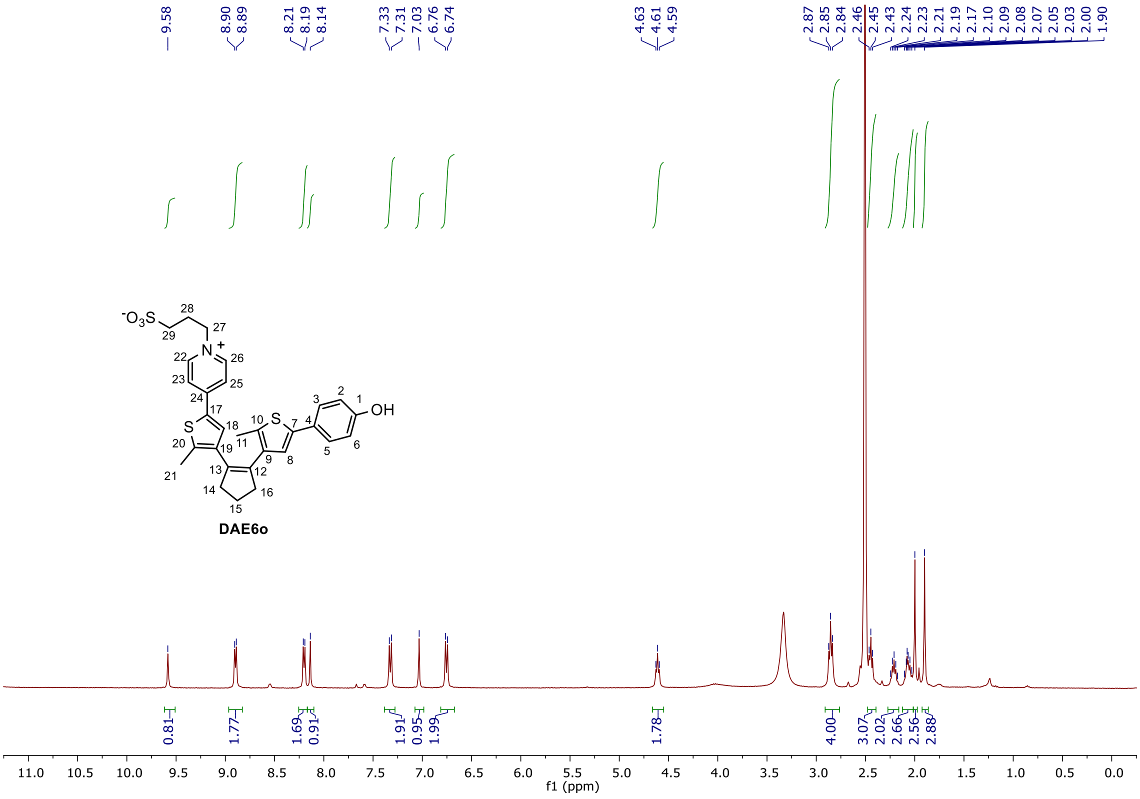

^{1}H NMR (400 MHz, DMSO)

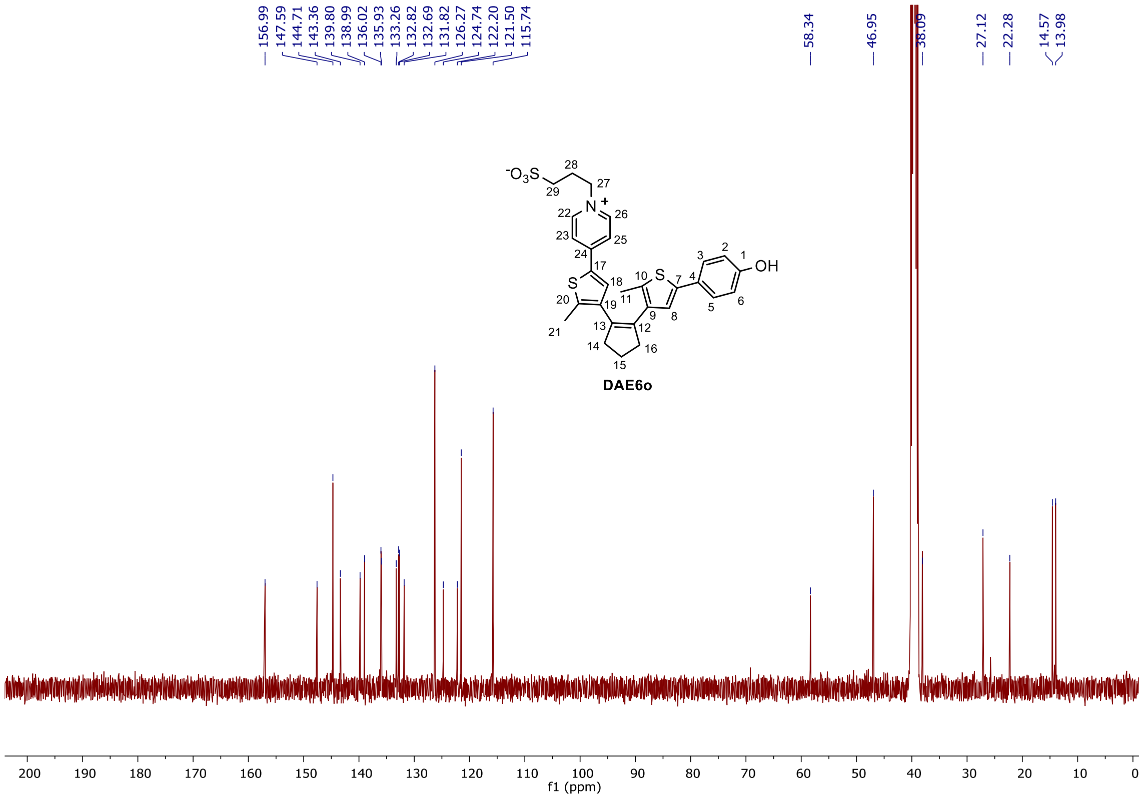

$^{13}C\{^{1}H\}$ NMR (101 MHz, DMSO)

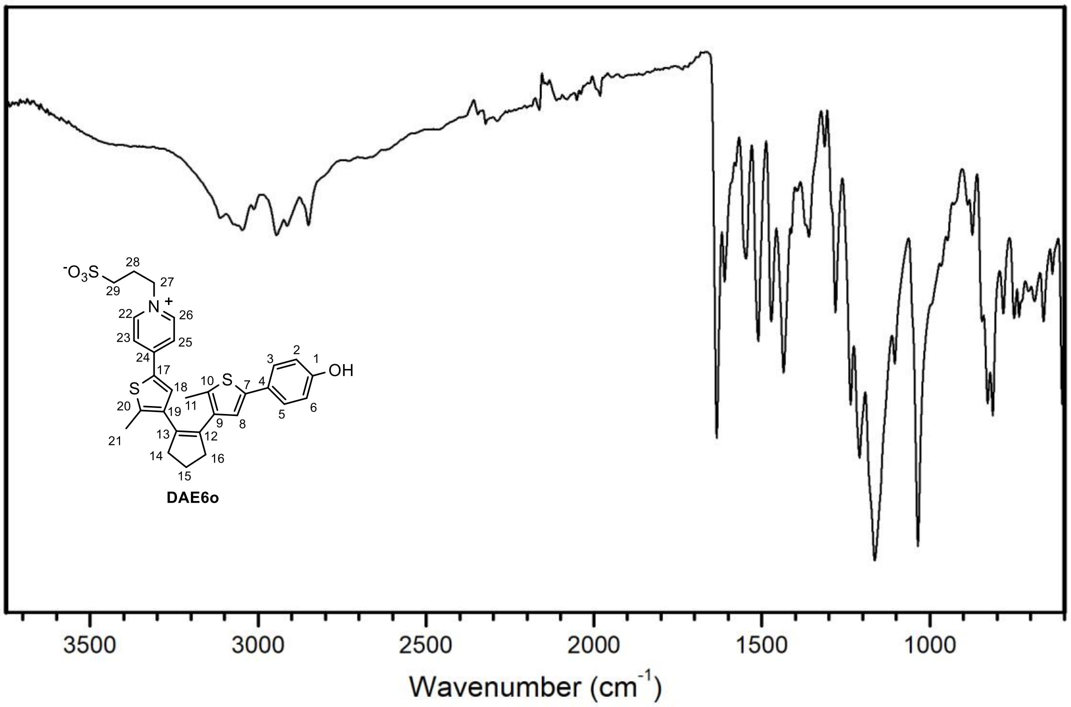

IR (ATR cm^{-1})

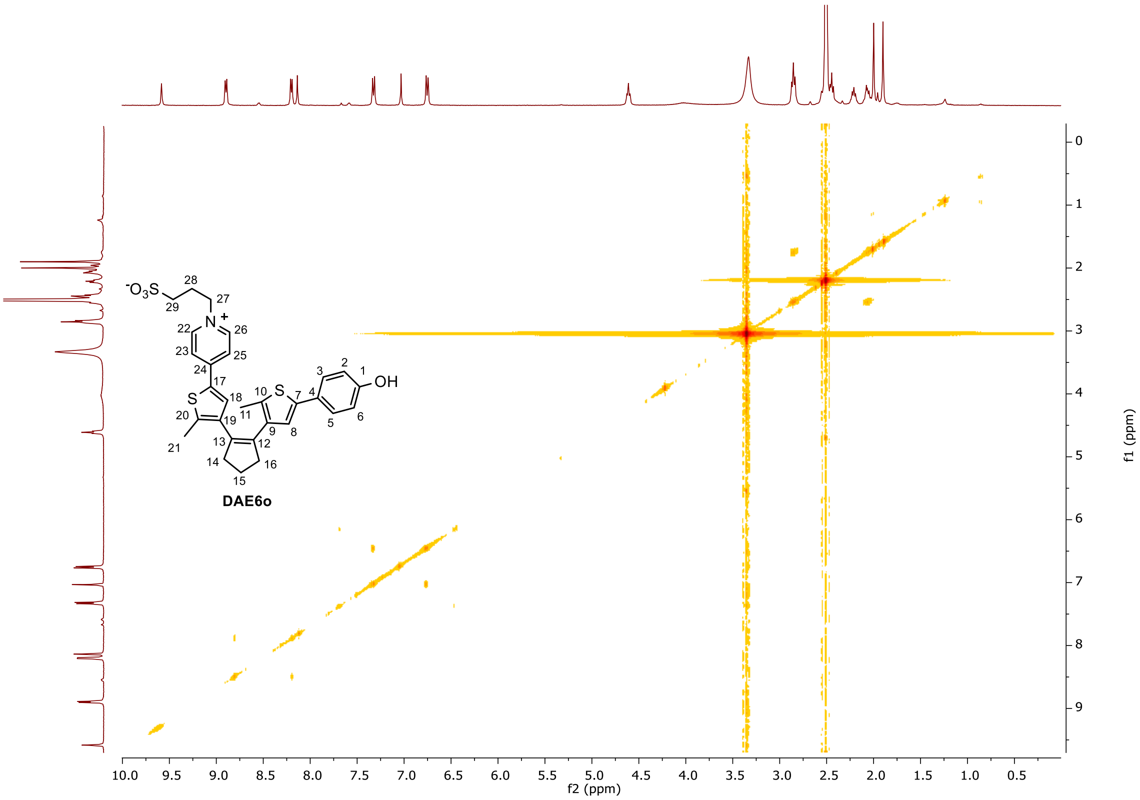

^{1}H-^{1}H COSY NMR (600 MHz, DMSO)

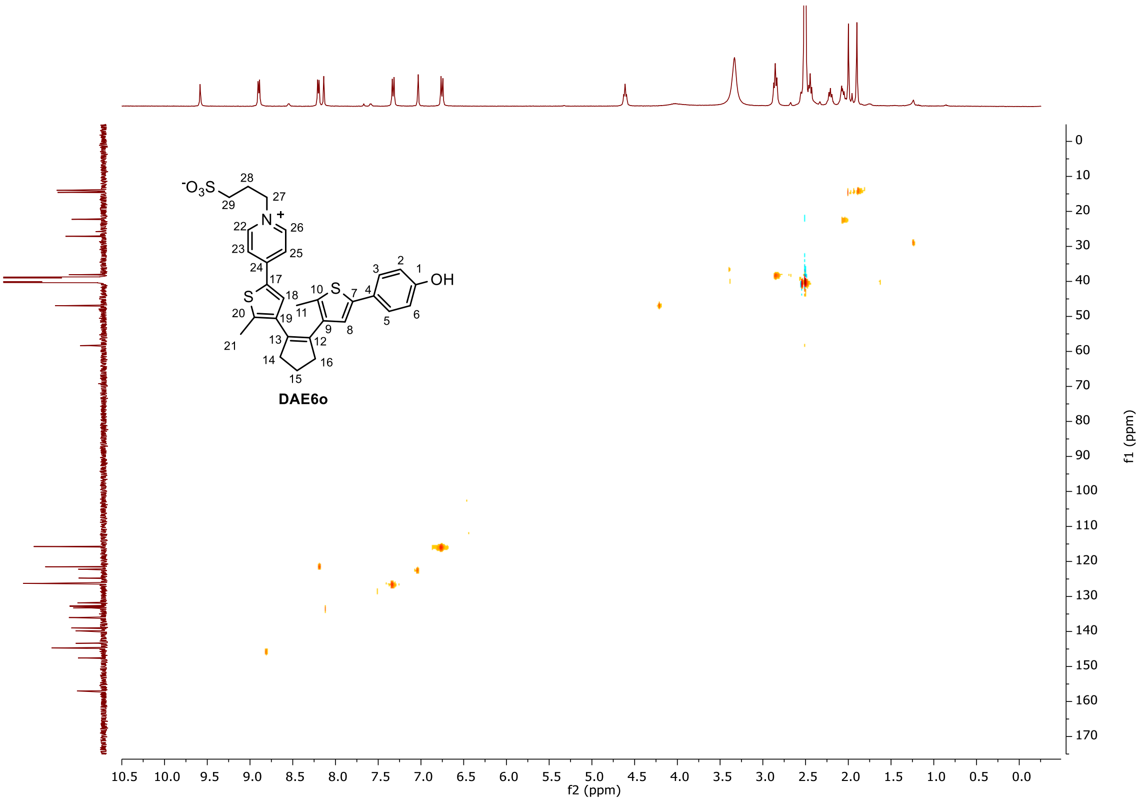

^{1}H-^{13}C HSQC NMR (600 MHz, DMSO)

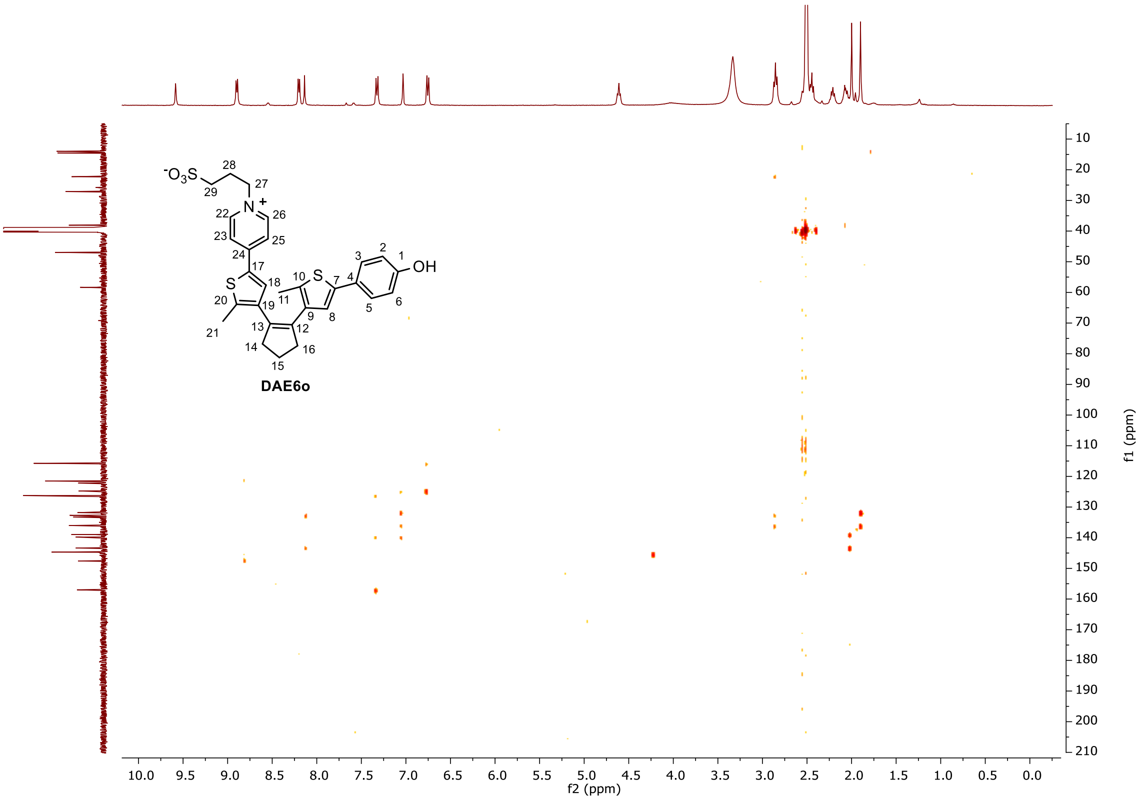

^1H-^{13}C HMBC NMR (600 MHz, DMSO)

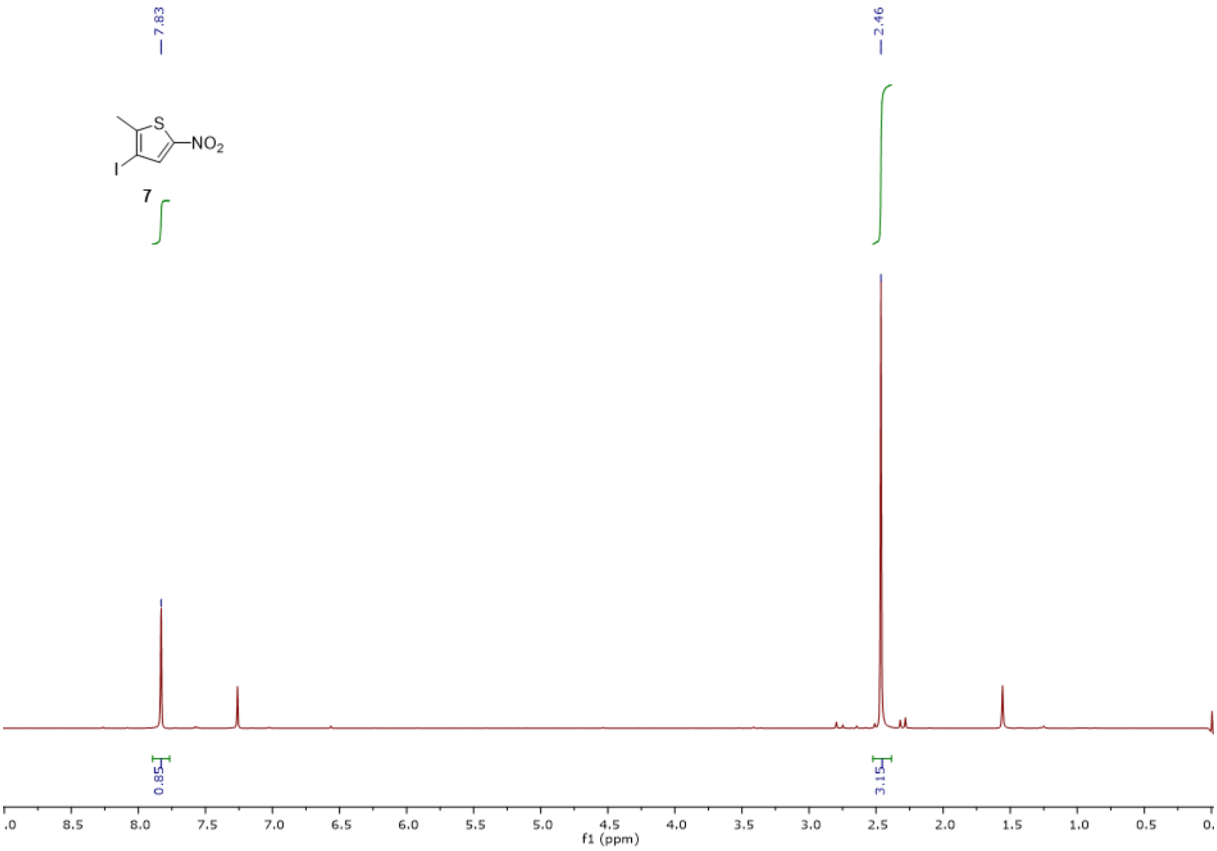

^1H NMR (360 MHz, $CDCl_3$)

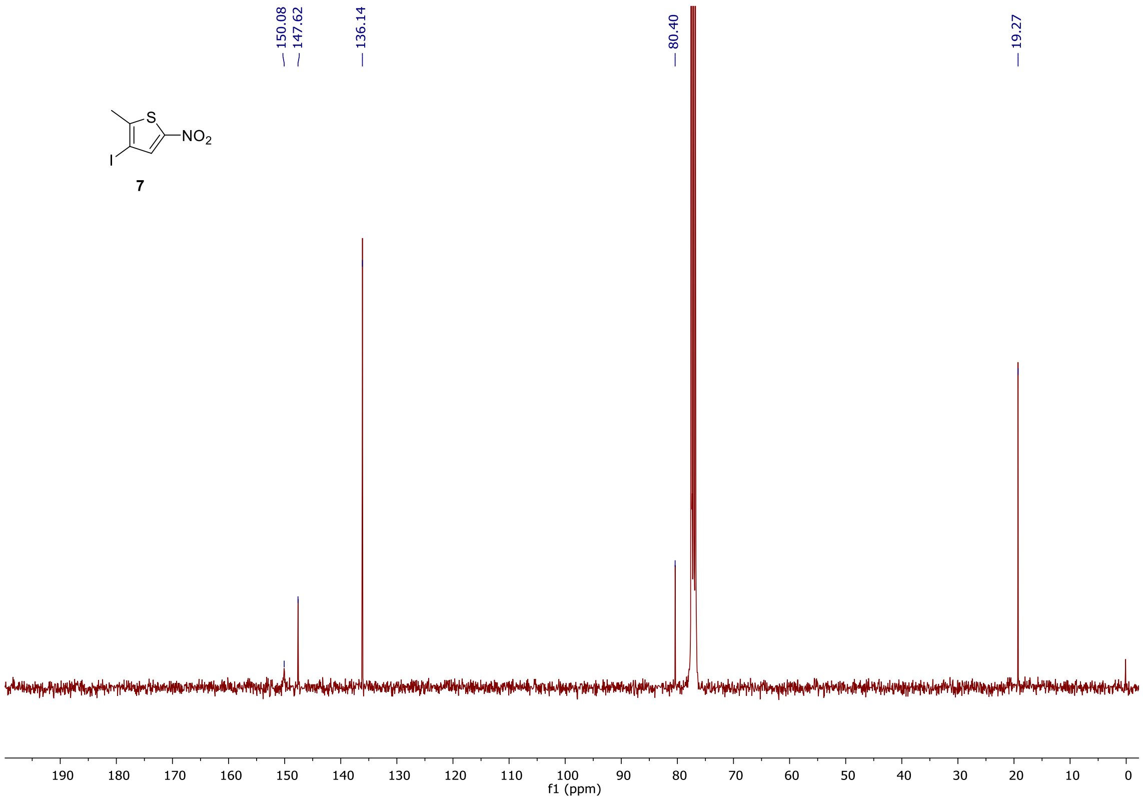

$^{13}C\{^1H\}$ NMR (91 MHz, $CDCl_3$)

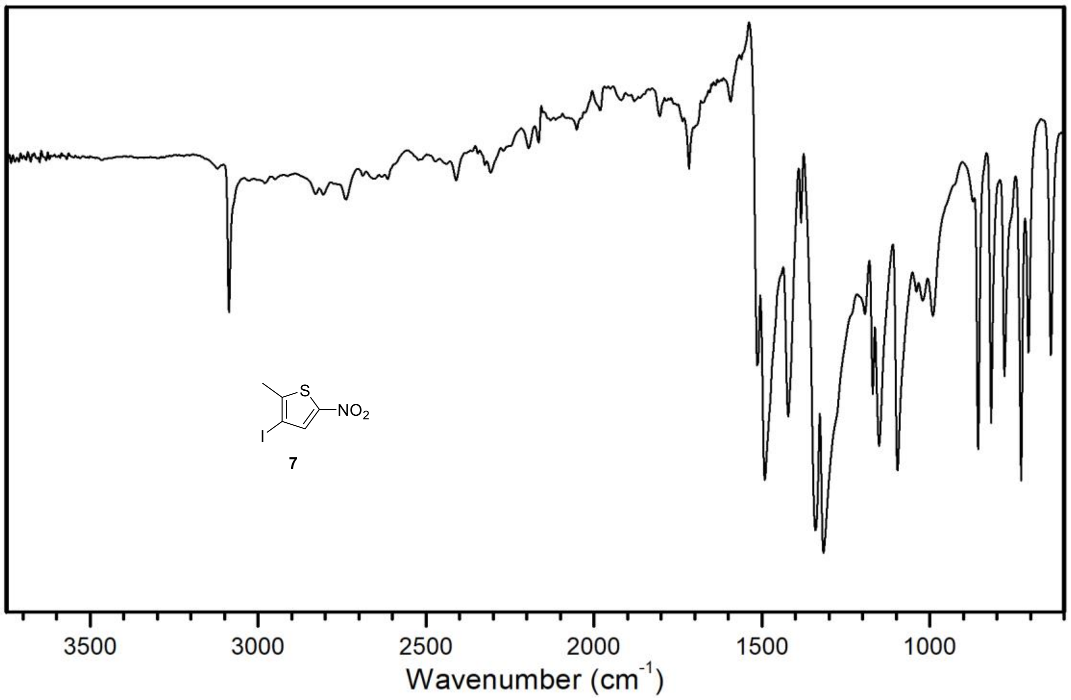

IR (ATR cm^{-1})

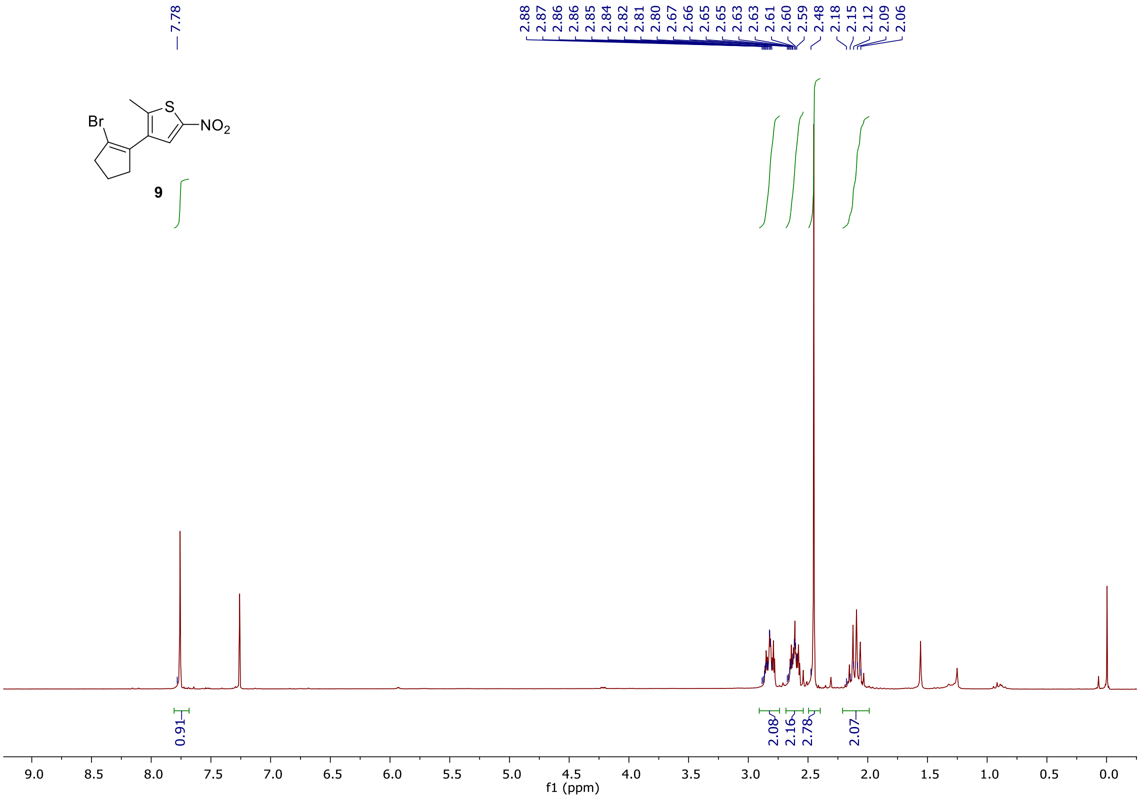

^{1}H NMR (250 MHz, $CDCl_3$)

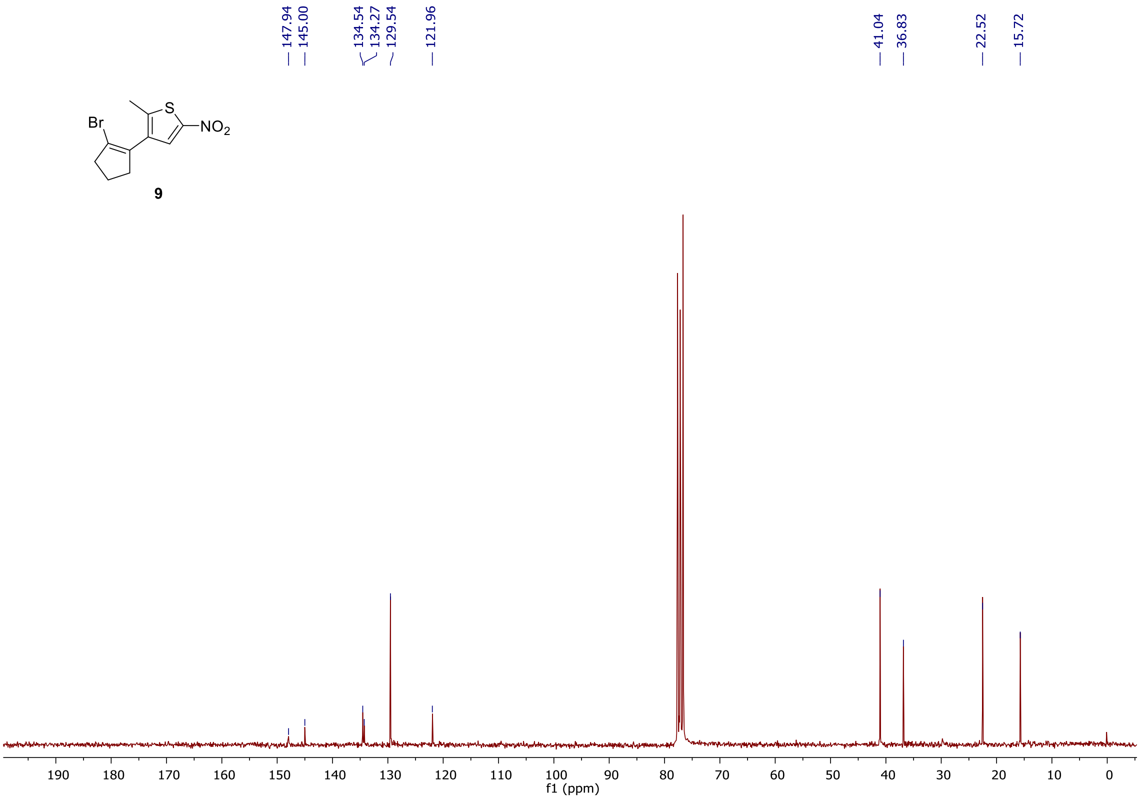

$^{13}C\{^{1}H\}$ NMR (63 MHz, $CDCl_3$)

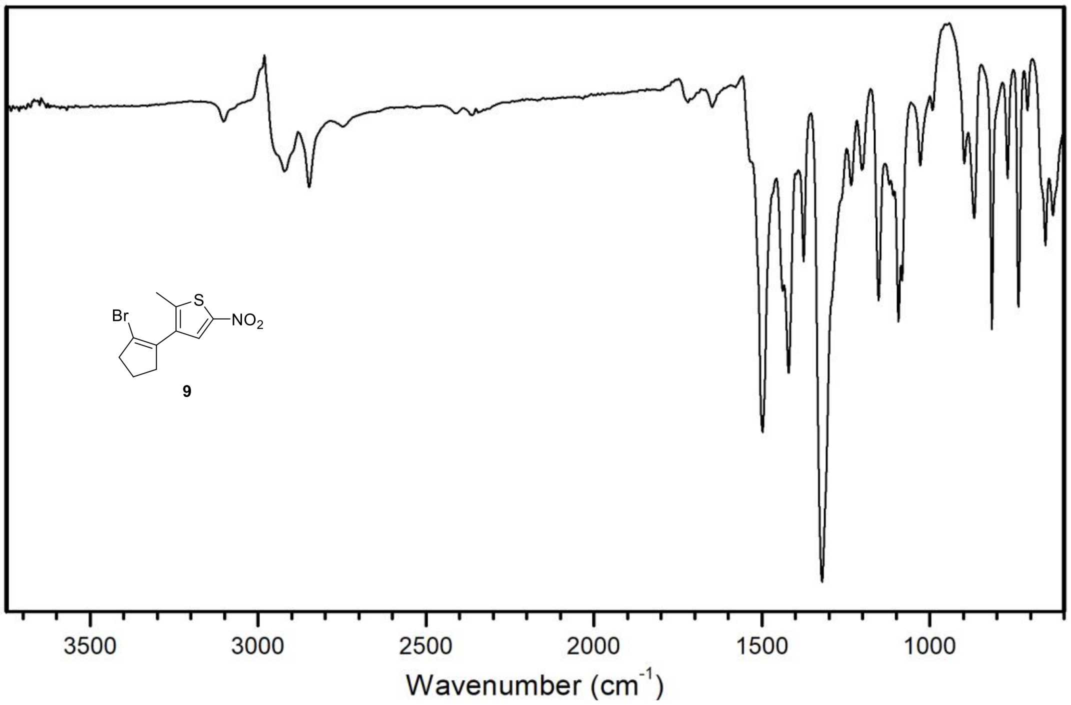

IR (ATR cm^{-1})

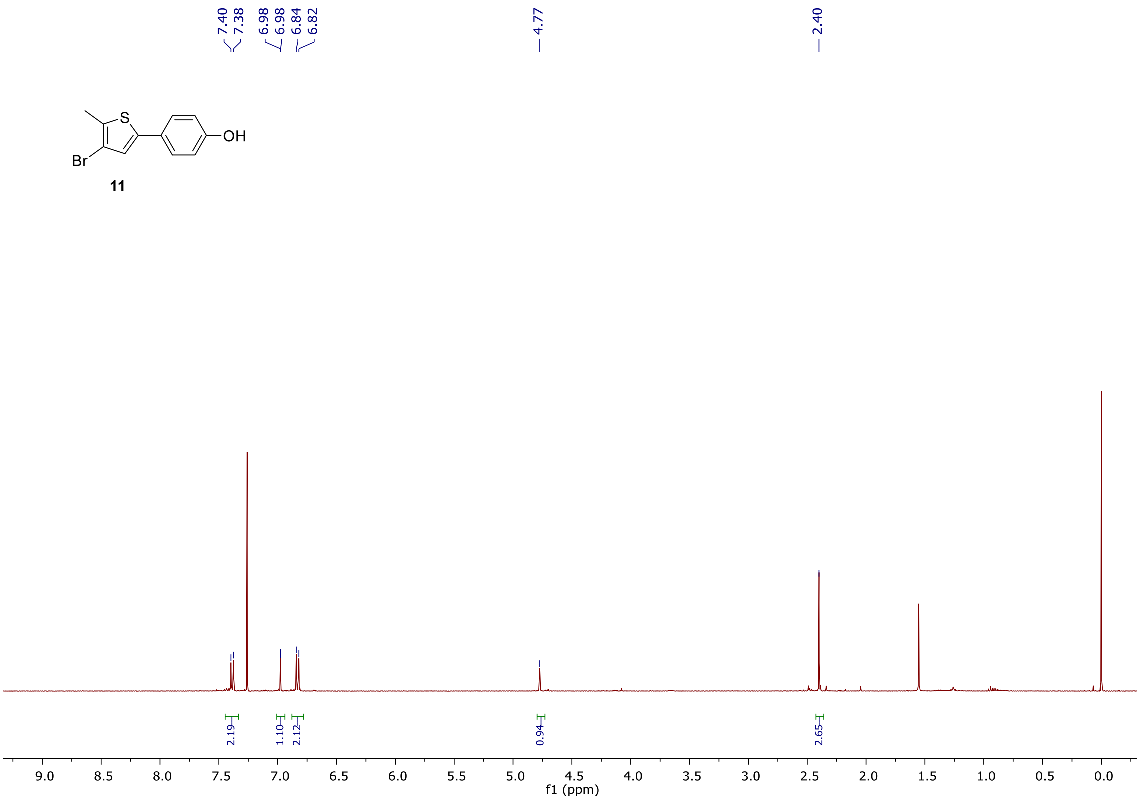

^{1}H NMR (250 MHz, $CDCl_3$)

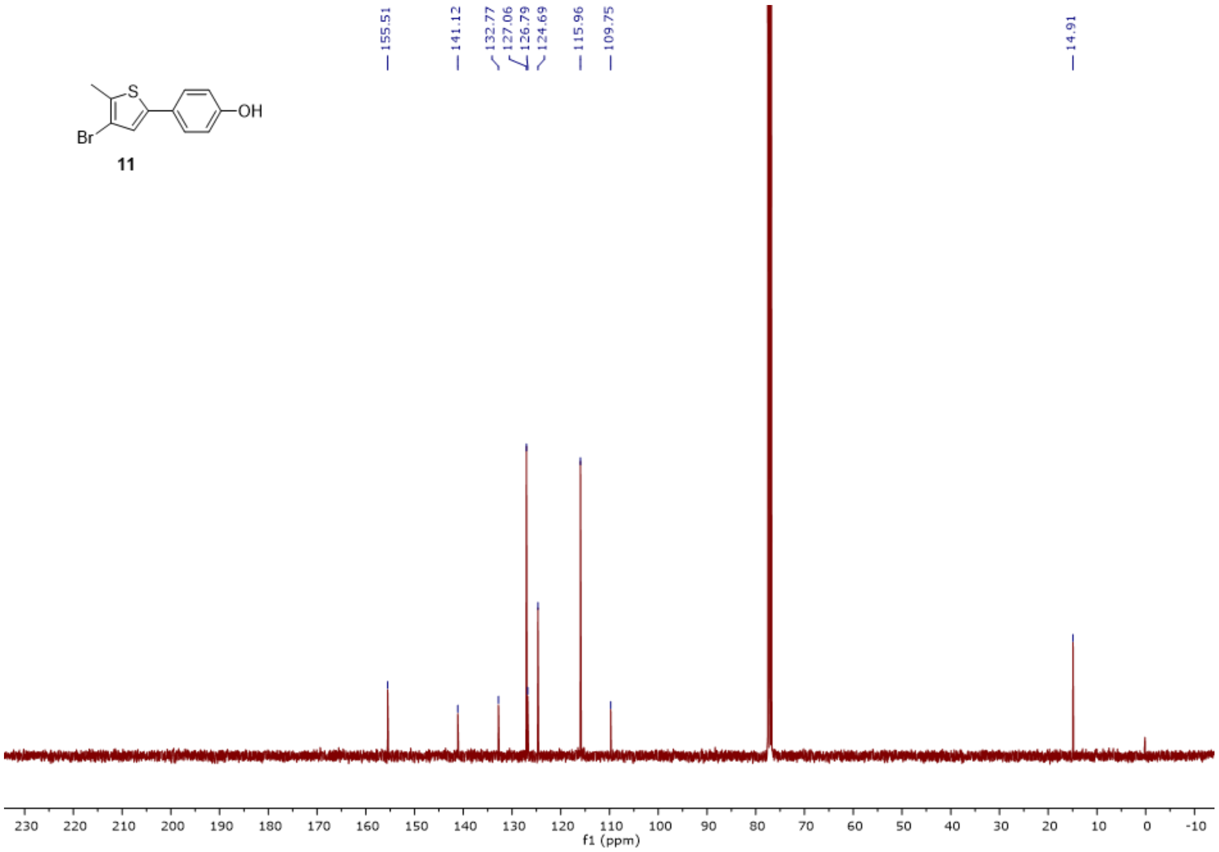

$^{13}C\{^{1}H\}$ **NMR (101 MHz, $CDCl_3$)**

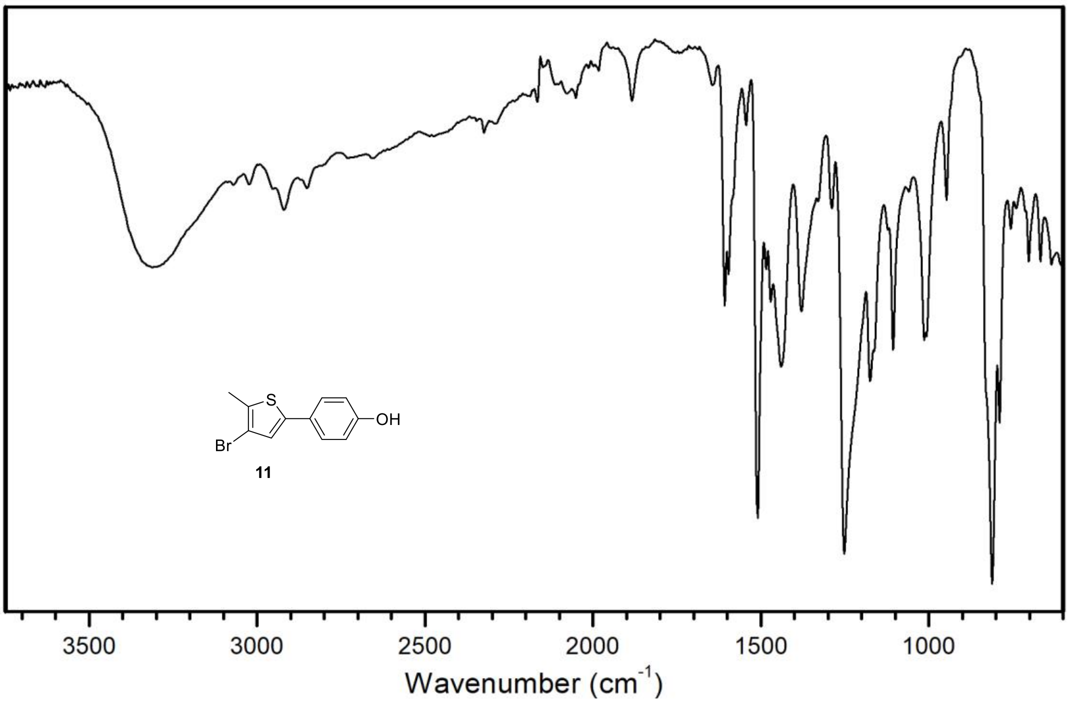

IR (ATR cm^{-1})

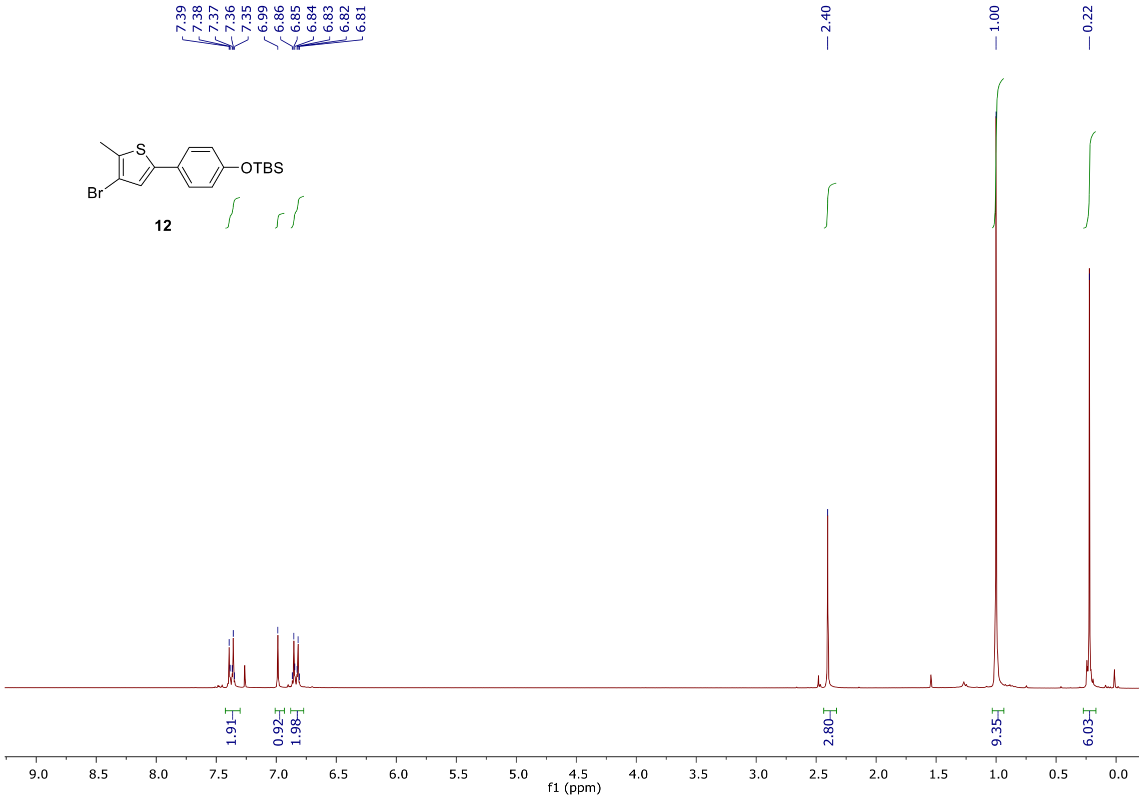

^{1}H NMR (250 MHz, $CDCl_3$)

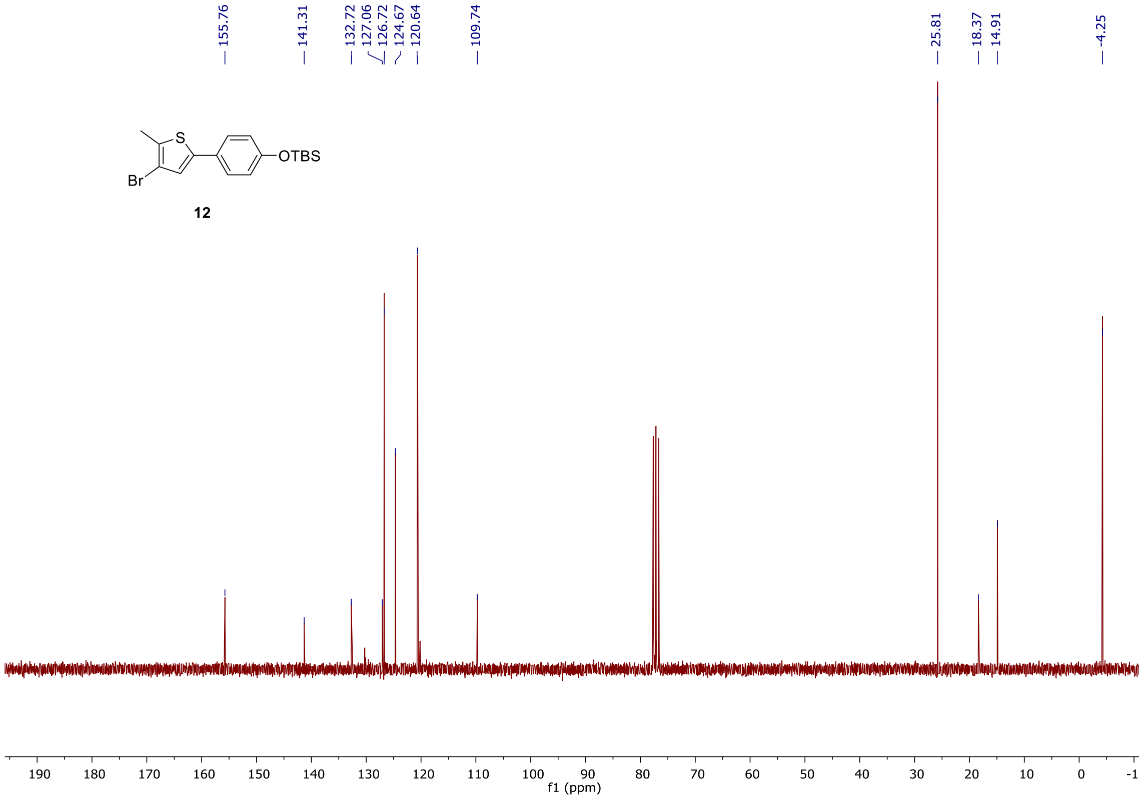

$^{13}C\{^{1}H\}$ NMR (63 MHz, $CDCl_3$)

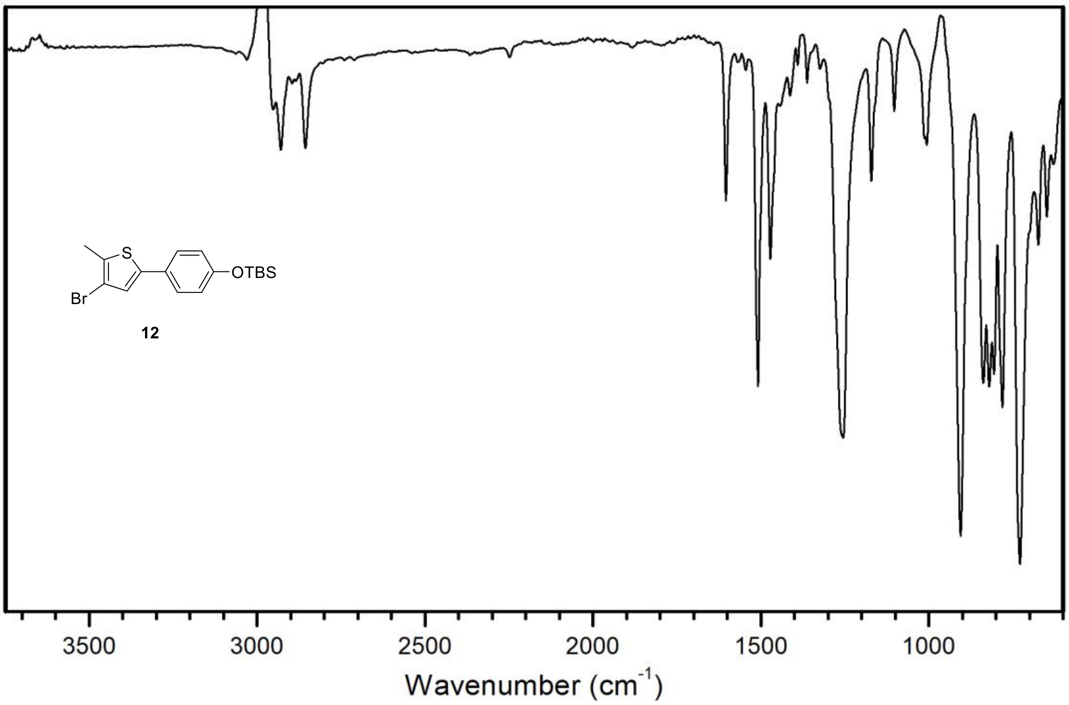

IR (ATR cm-1)

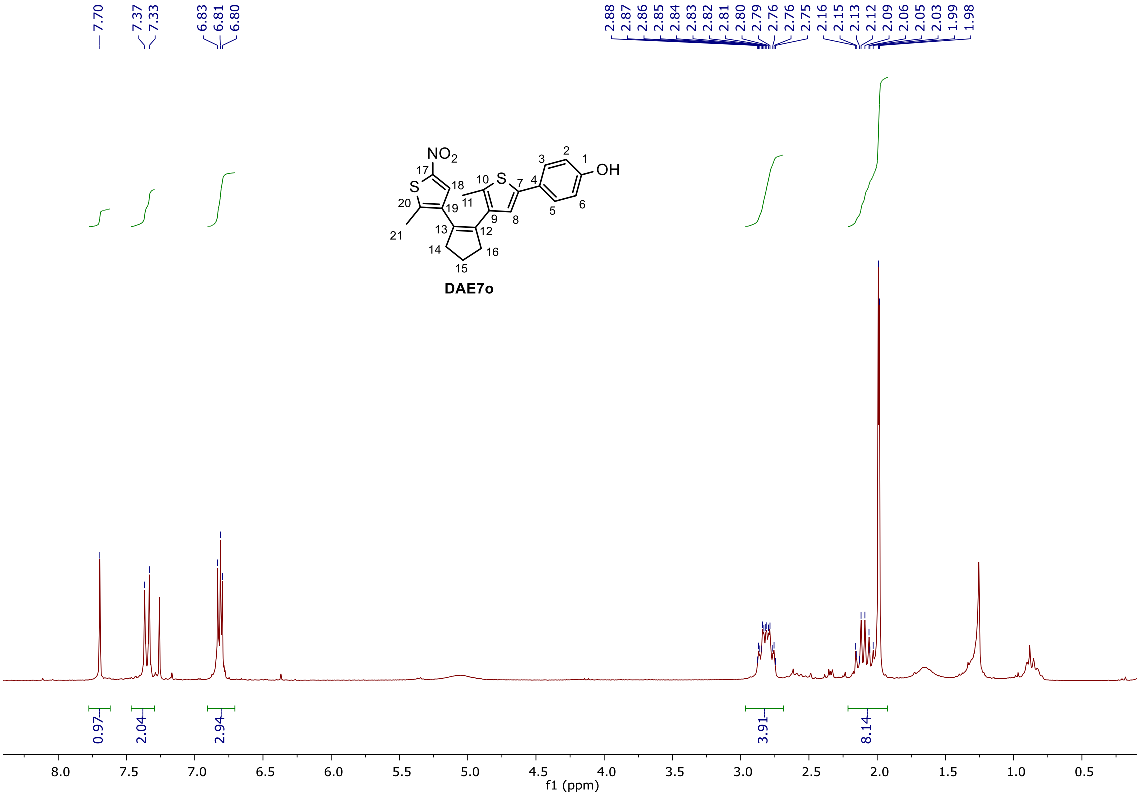

^{1}H NMR (400 MHz, $CDCl_3$)

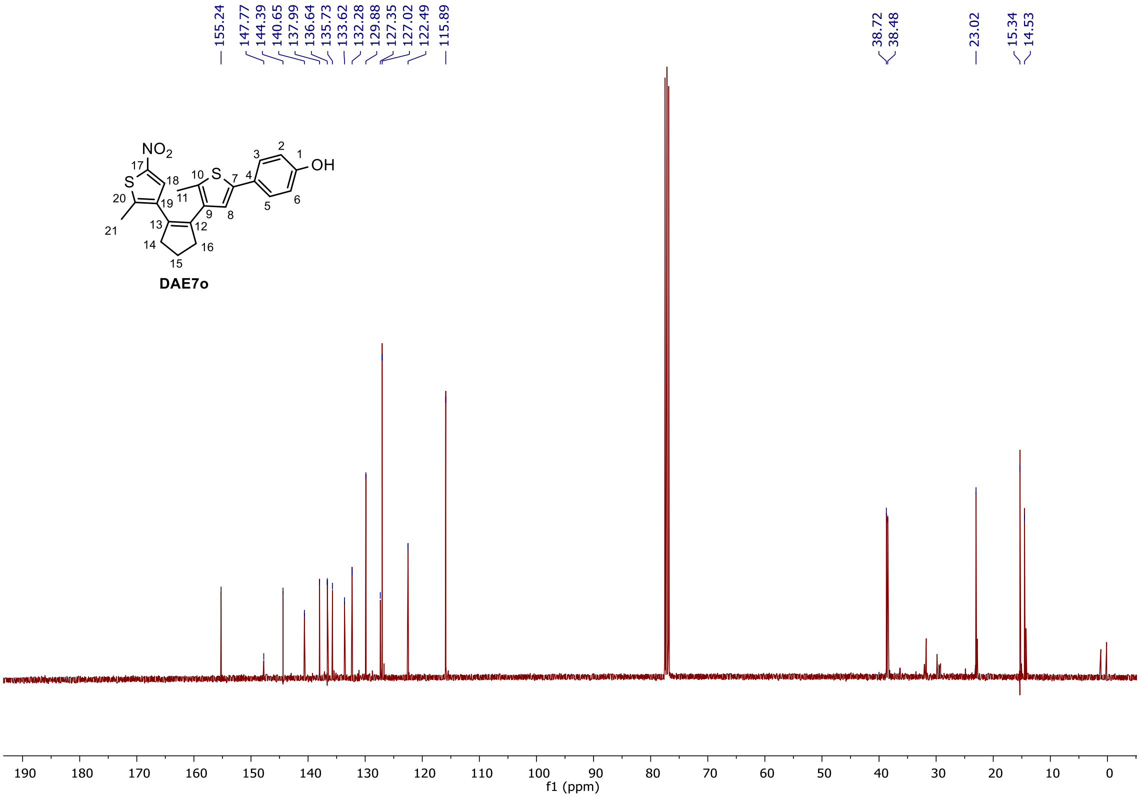

$^{13}C\{^{1}H\}$ NMR (101 MHz, $CDCl_3$)

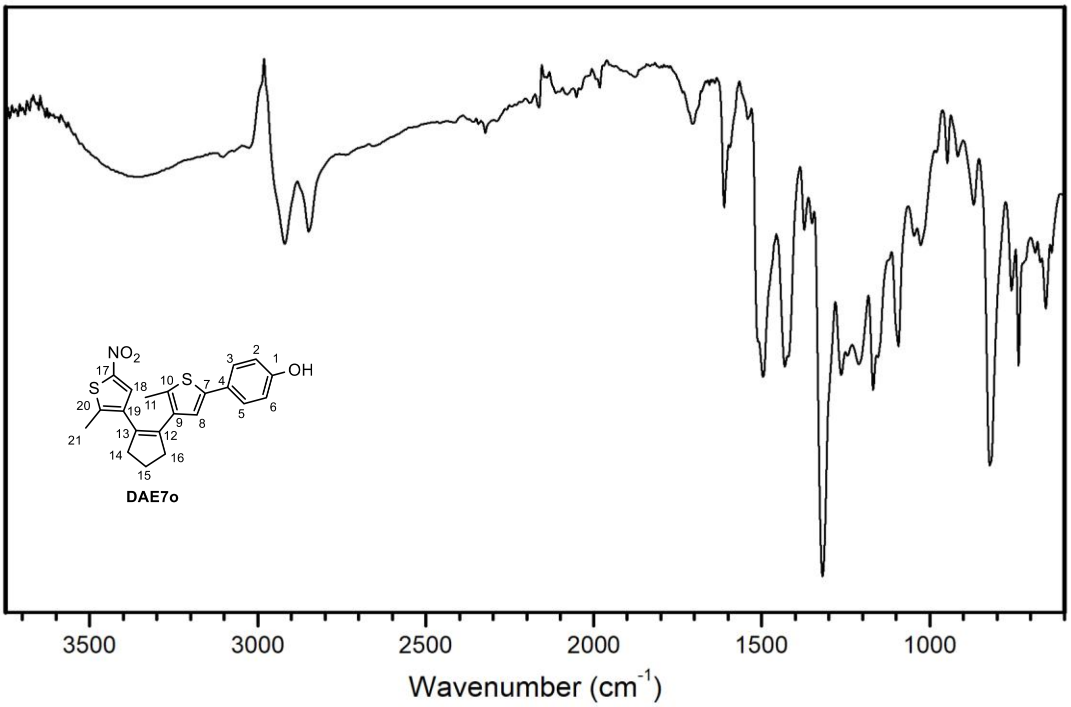

IR (ATR cm^{-1})

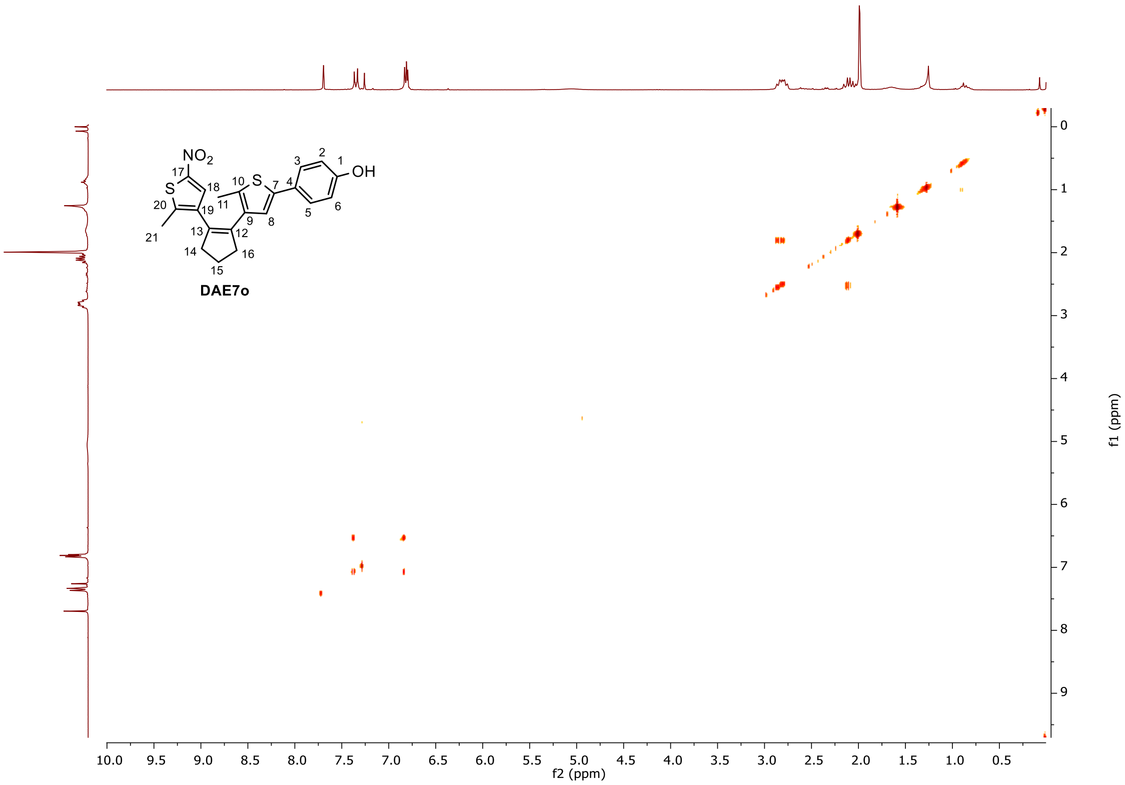

^{1}H-^{1}H COSY NMR (600 MHz, CDCl$_3$)

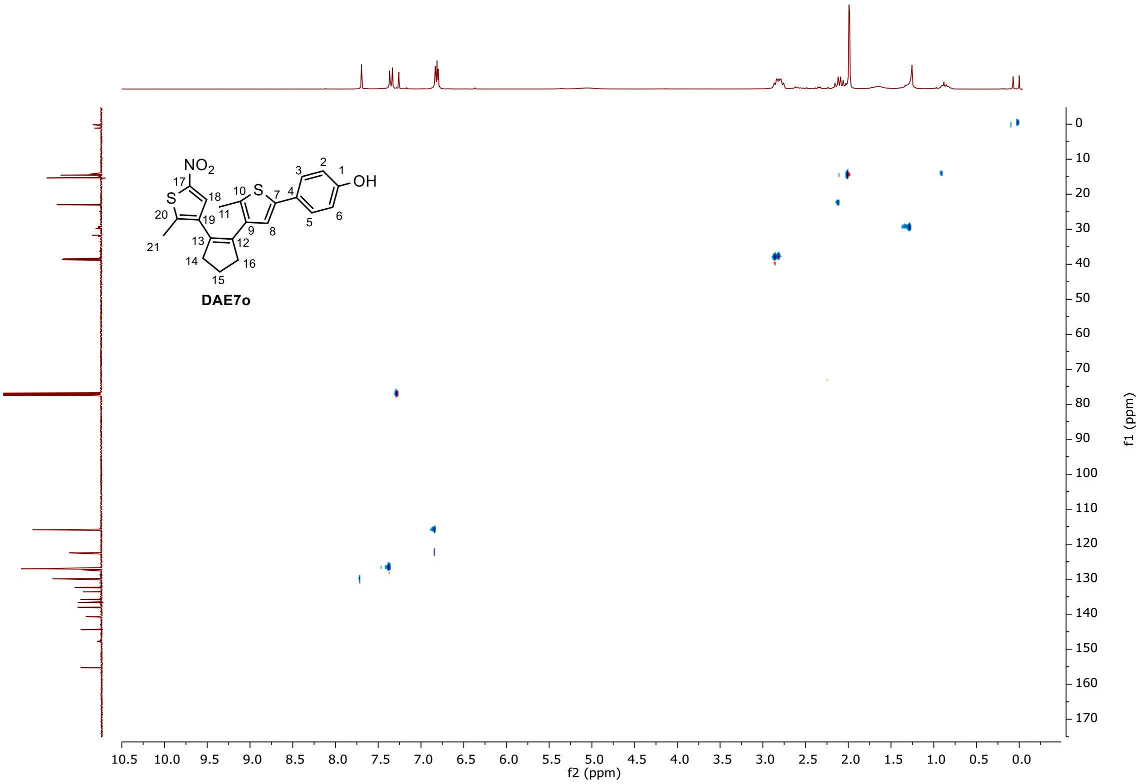

^{1}H-^{13}C HSQC NMR (600 MHz, CDCl$_3$)

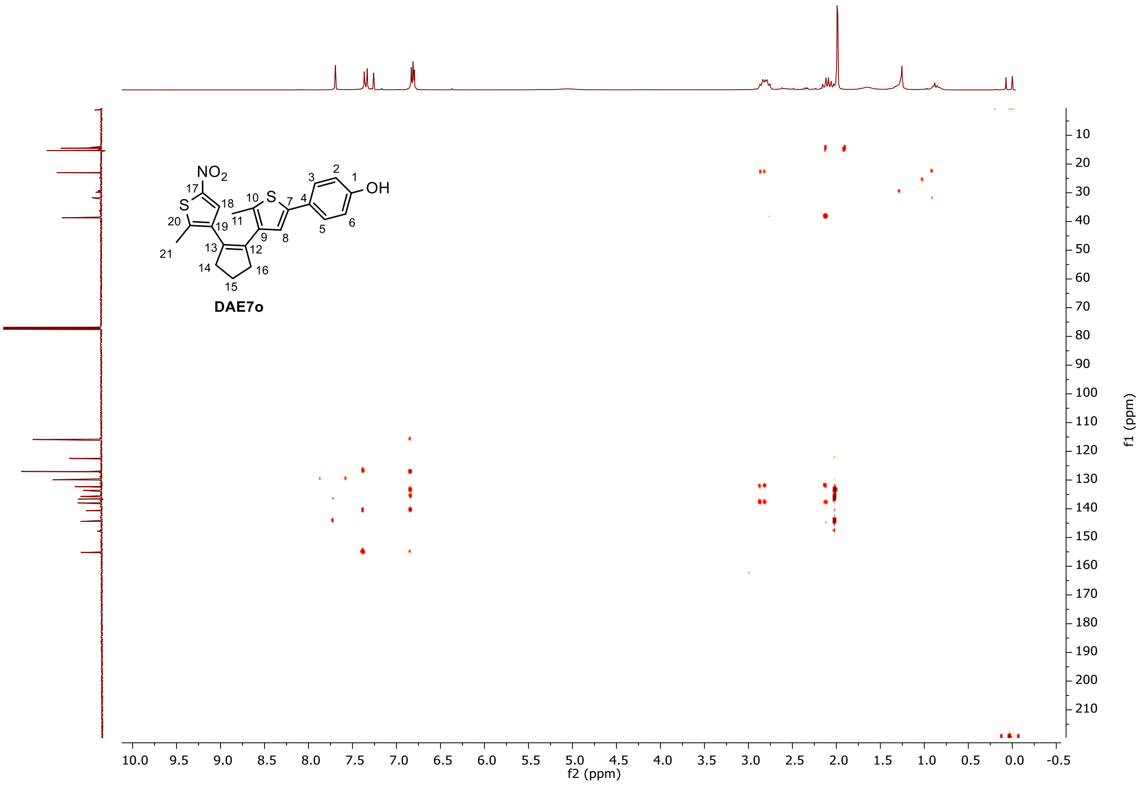

^{1}H-^{13}C HMBC NMR (600 MHz, CDCl$_3$)

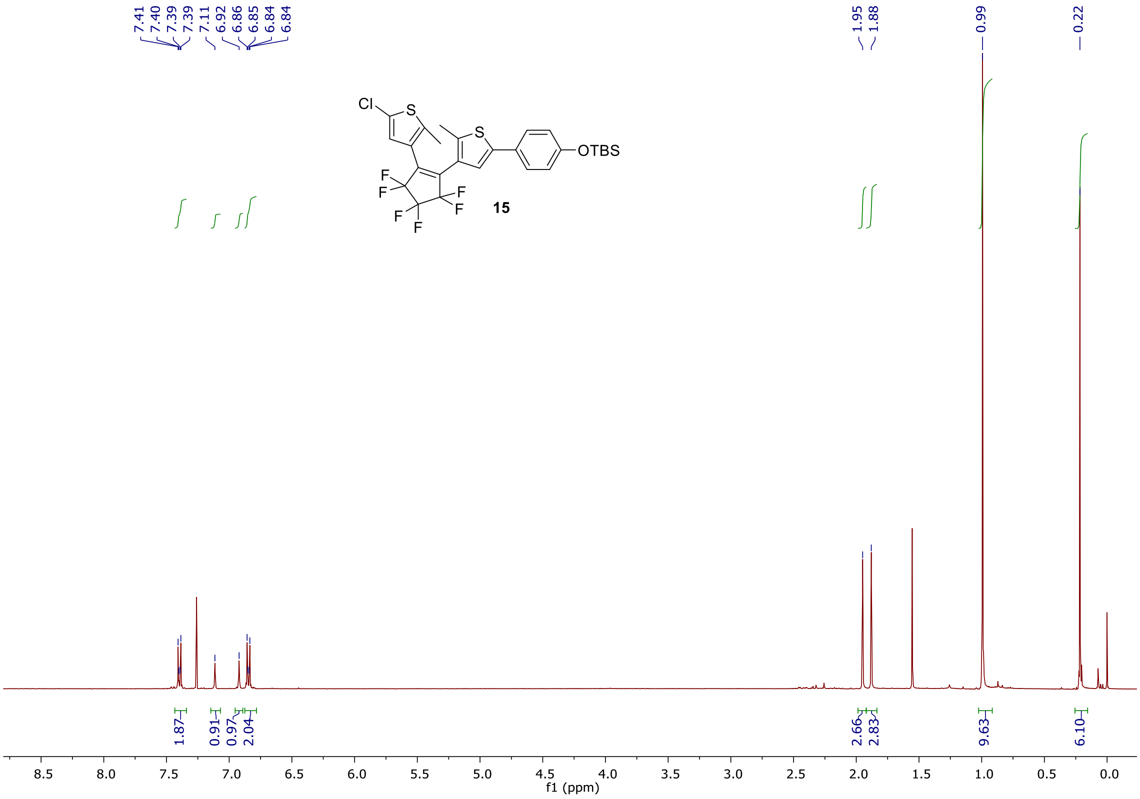

^{1}H NMR (400 MHz, CDCl$_3$)

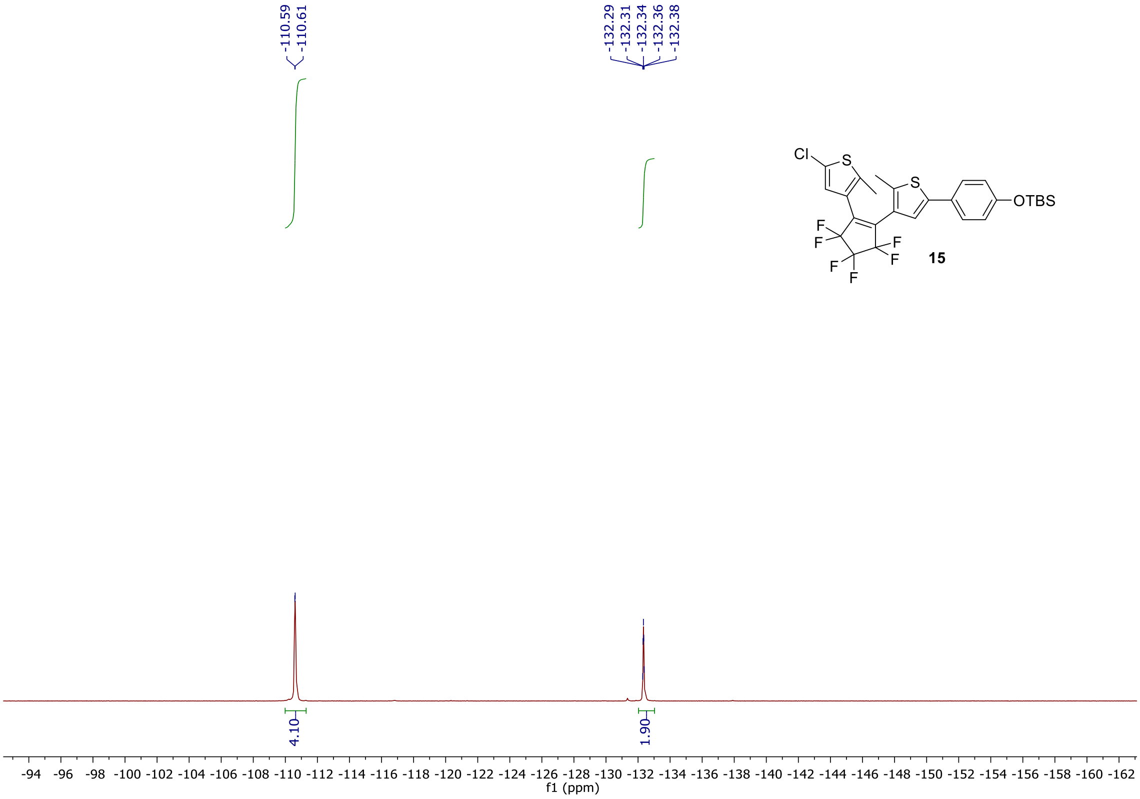

^{19}F NMR (235 MHz, $CDCl_3$)

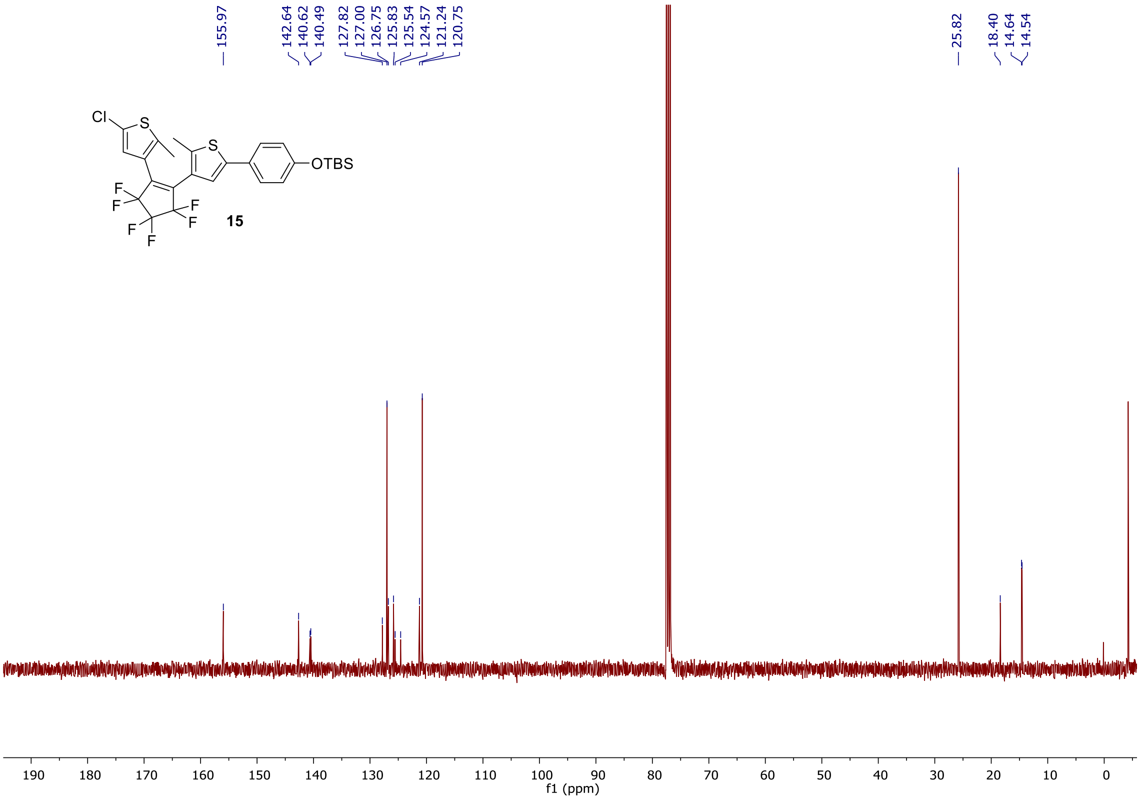

$^{13}C\{^1H\}$ NMR (101 MHz, $CDCl_3$)

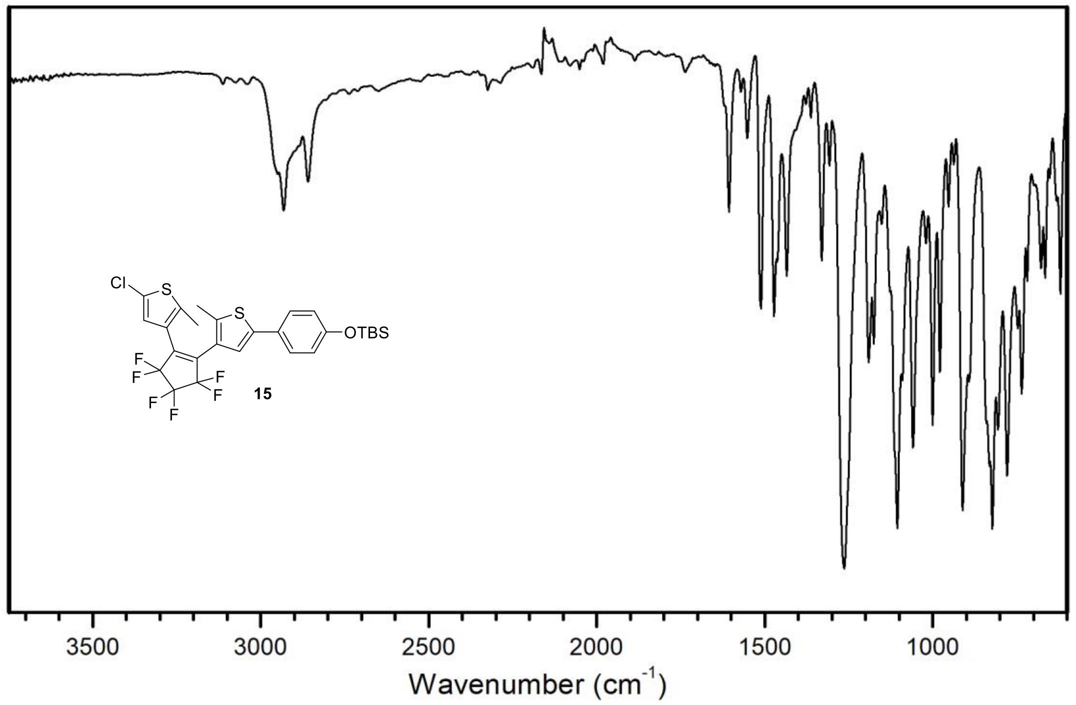

IR (ATR cm^{-1})

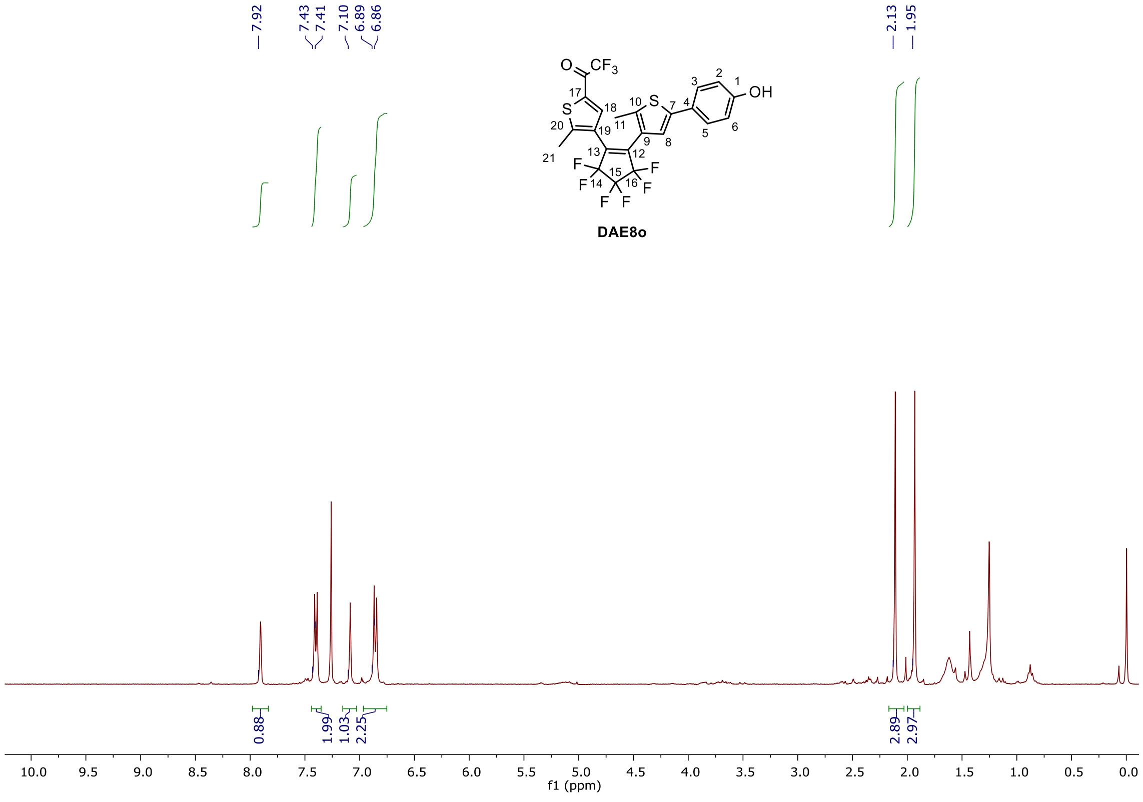

^{1}H NMR (360 MHz, $CDCl_3$)

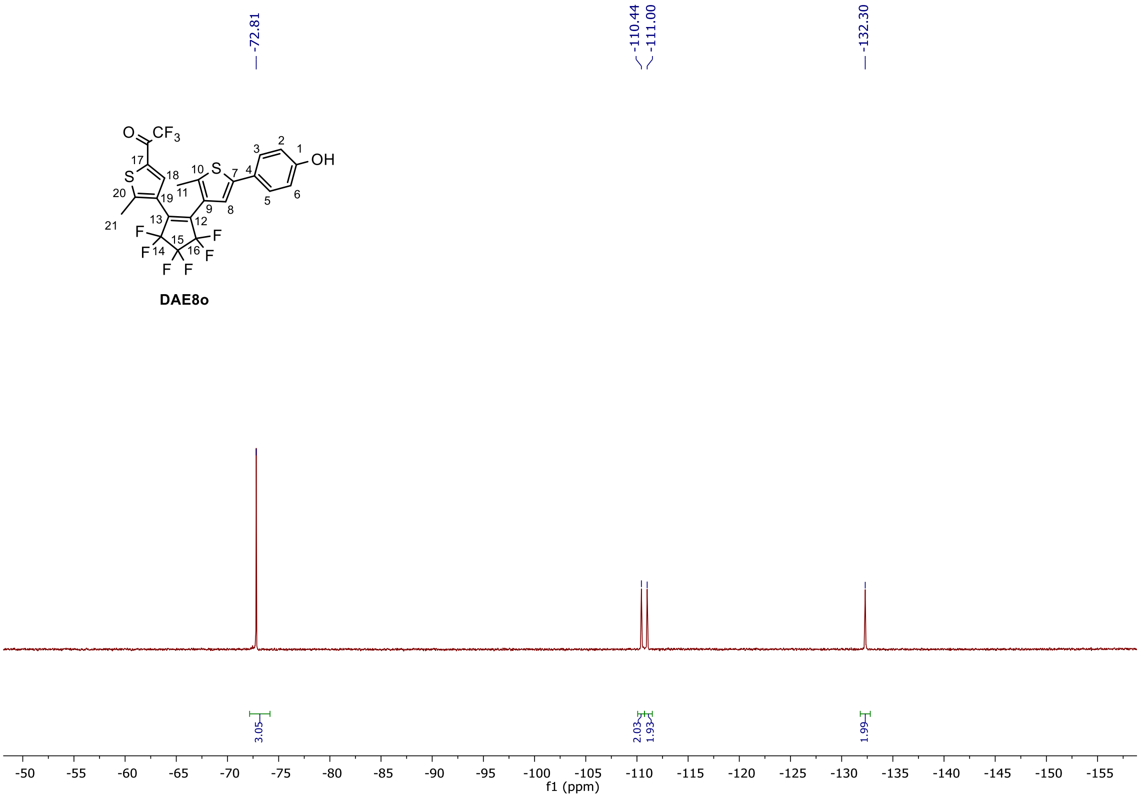

^{19}F NMR (235 MHz, $CDCl_3$)

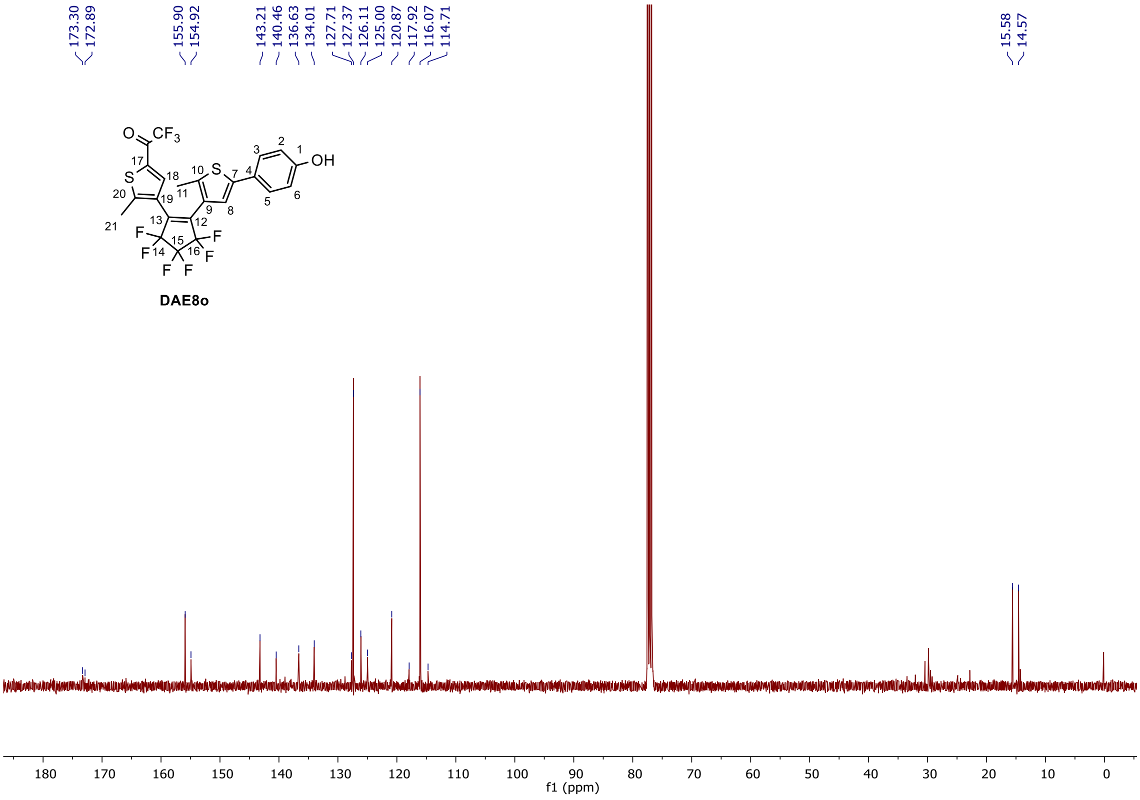

$^{13}C\{^1H\}$ NMR (91 MHz, $CDCl_3$)

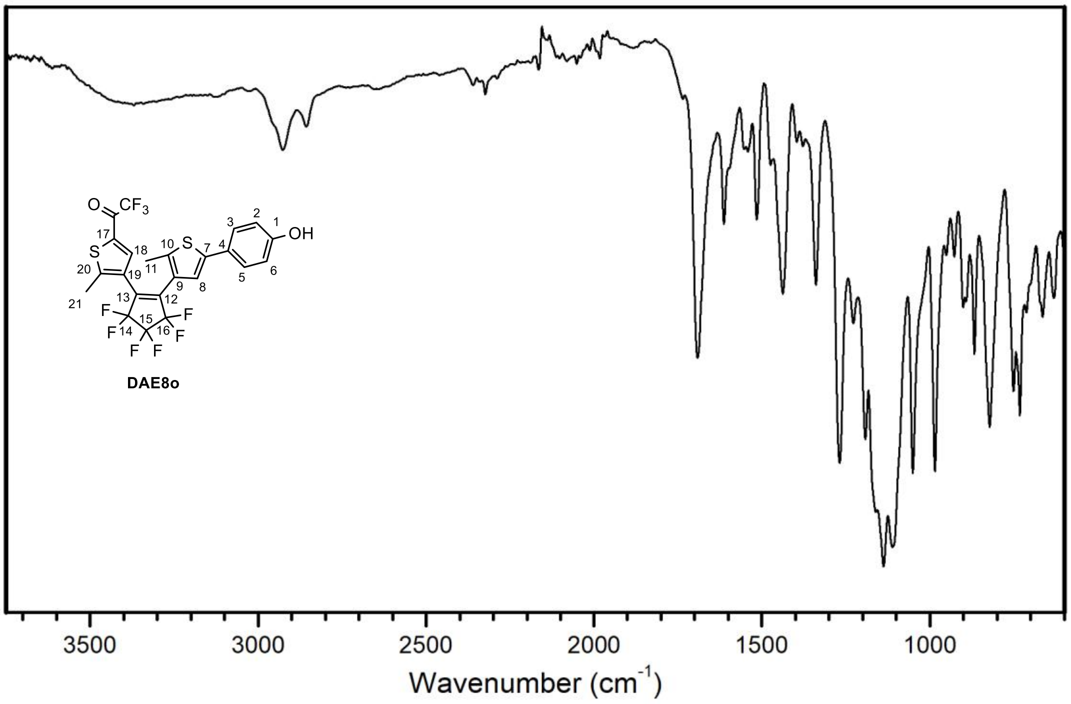

IR (ATR cm-1)

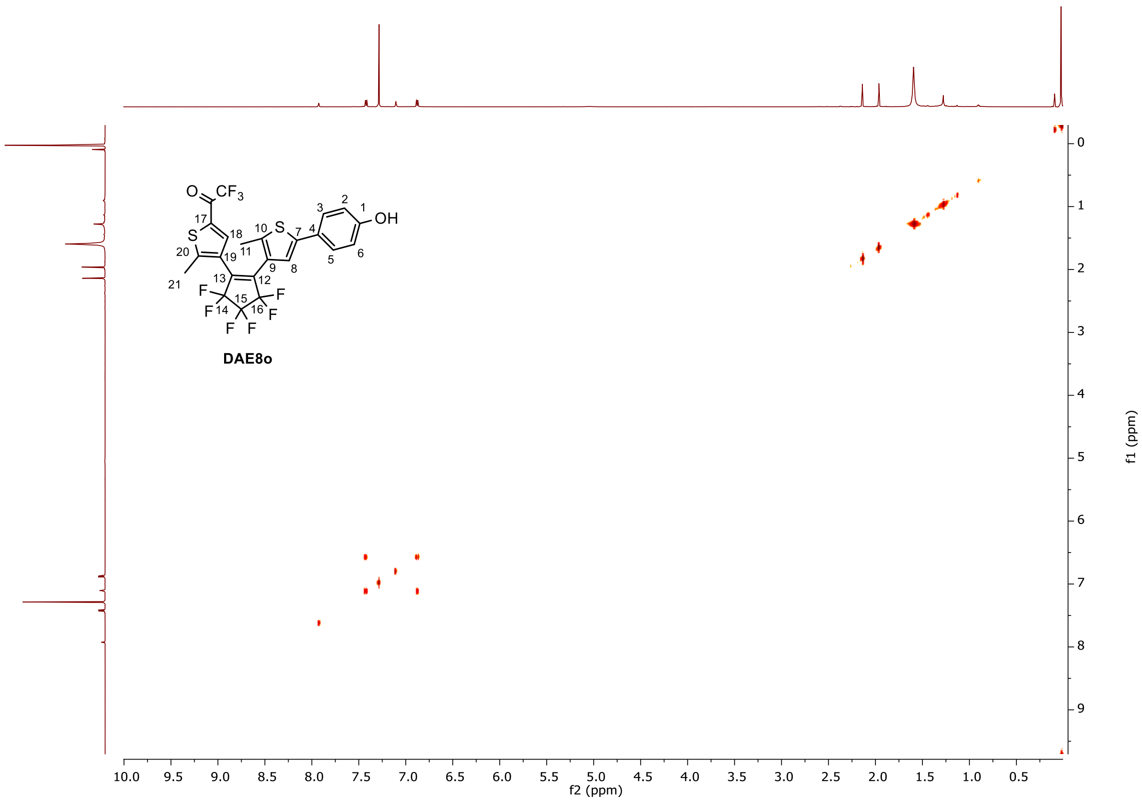

^{1}H-^{1}H COSY NMR (600 MHz, $CDCl_3$)

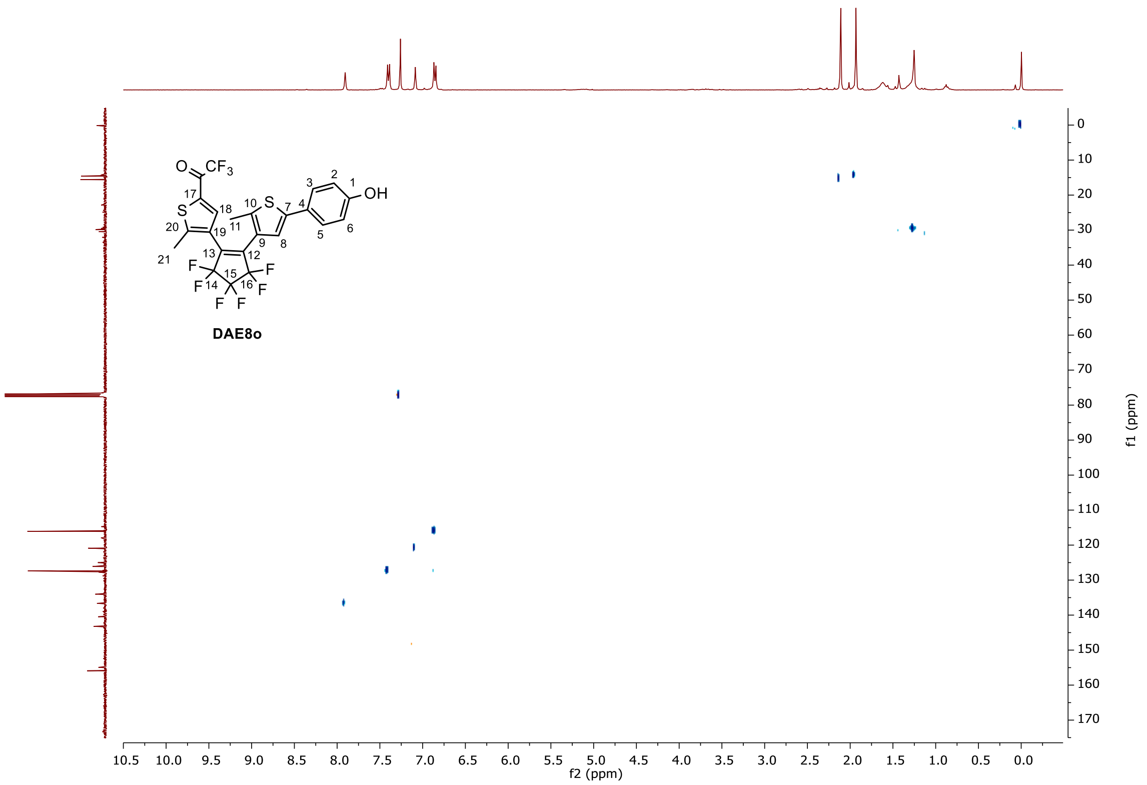

^1H-^{13}C HSQC NMR (600 MHz, $CDCl_3$)

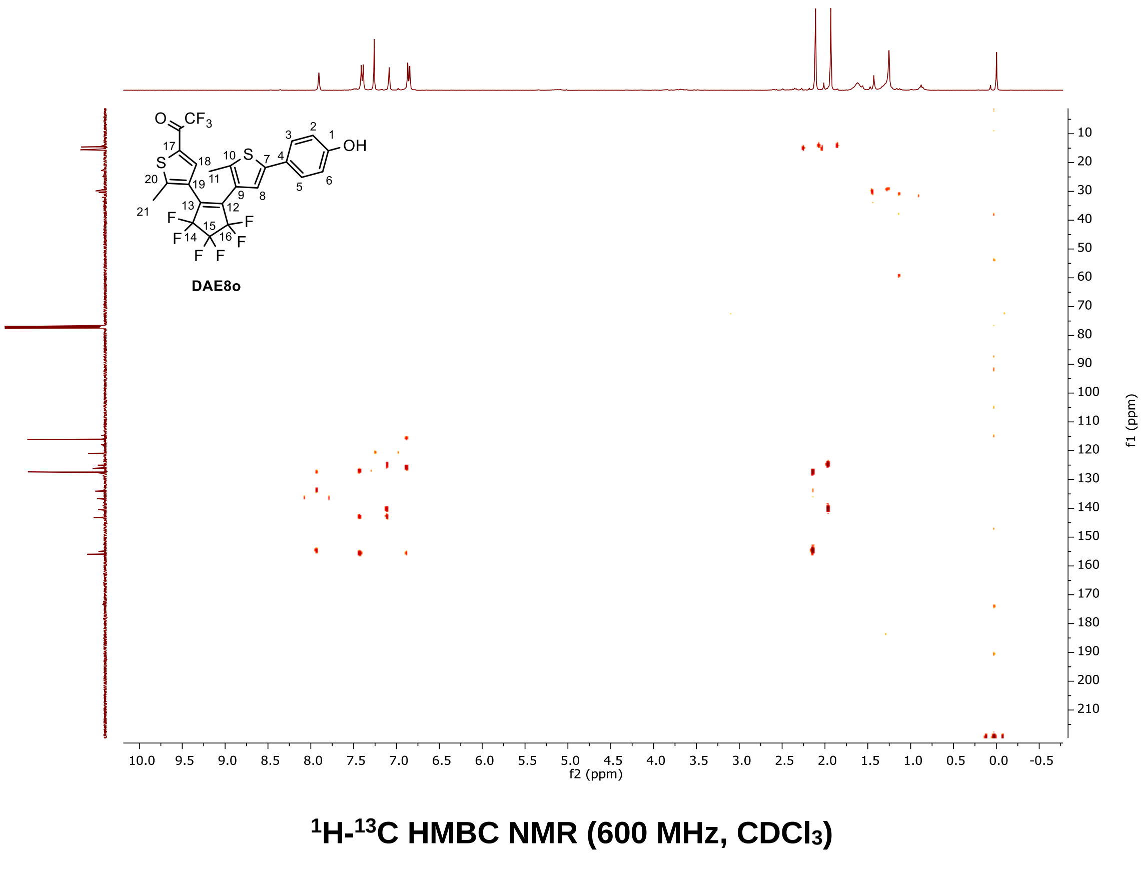

^1H-^{13}C HMBC NMR (600 MHz, $CDCl_3$)

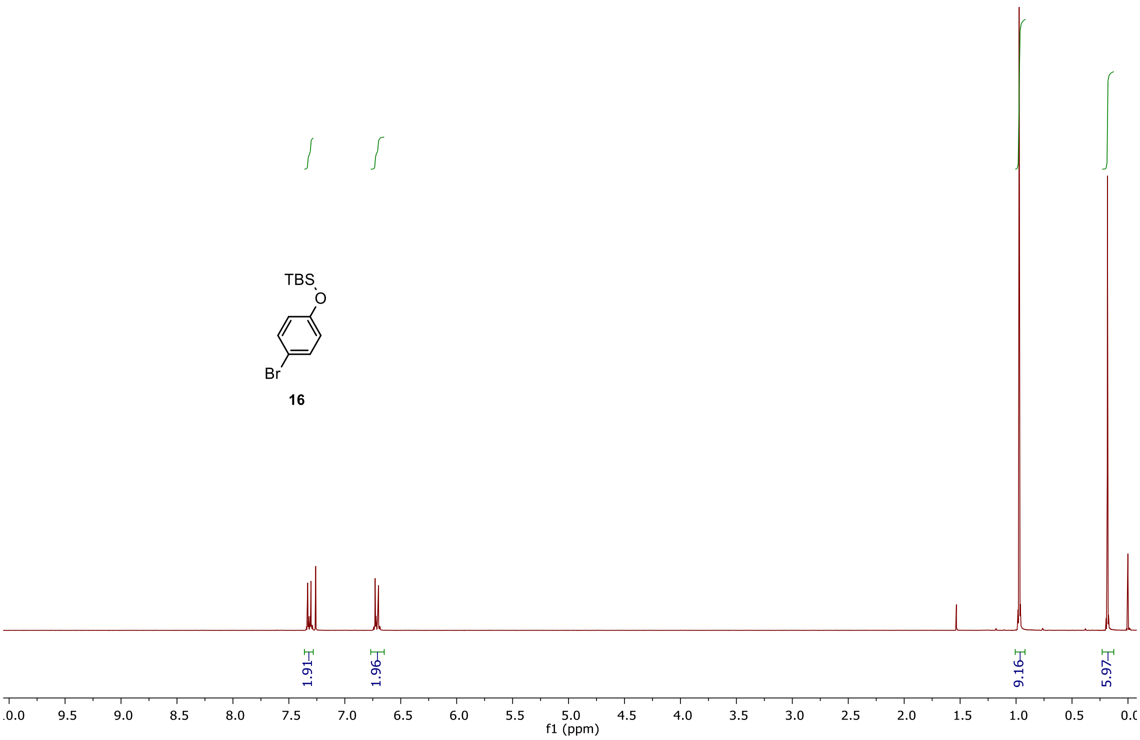

^{1}H NMR (300 MHz, CDCl$_3$)

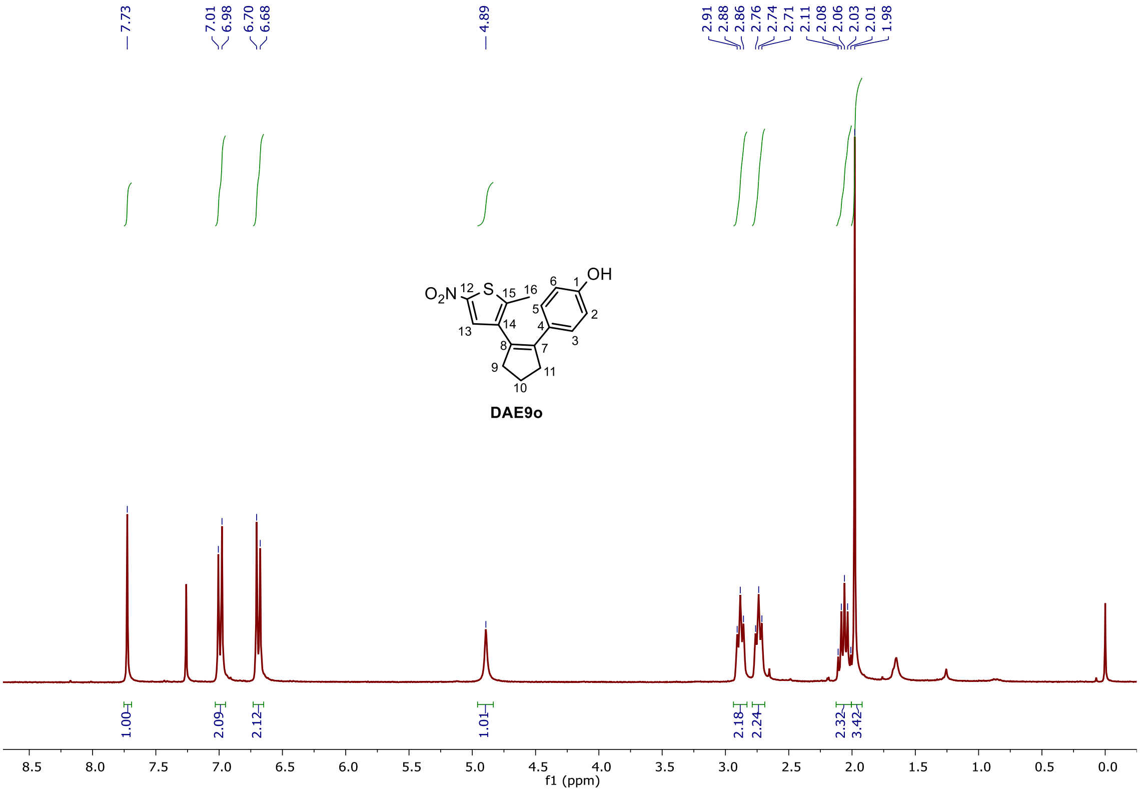

^{1}H NMR (300 MHz, CDCl$_3$)

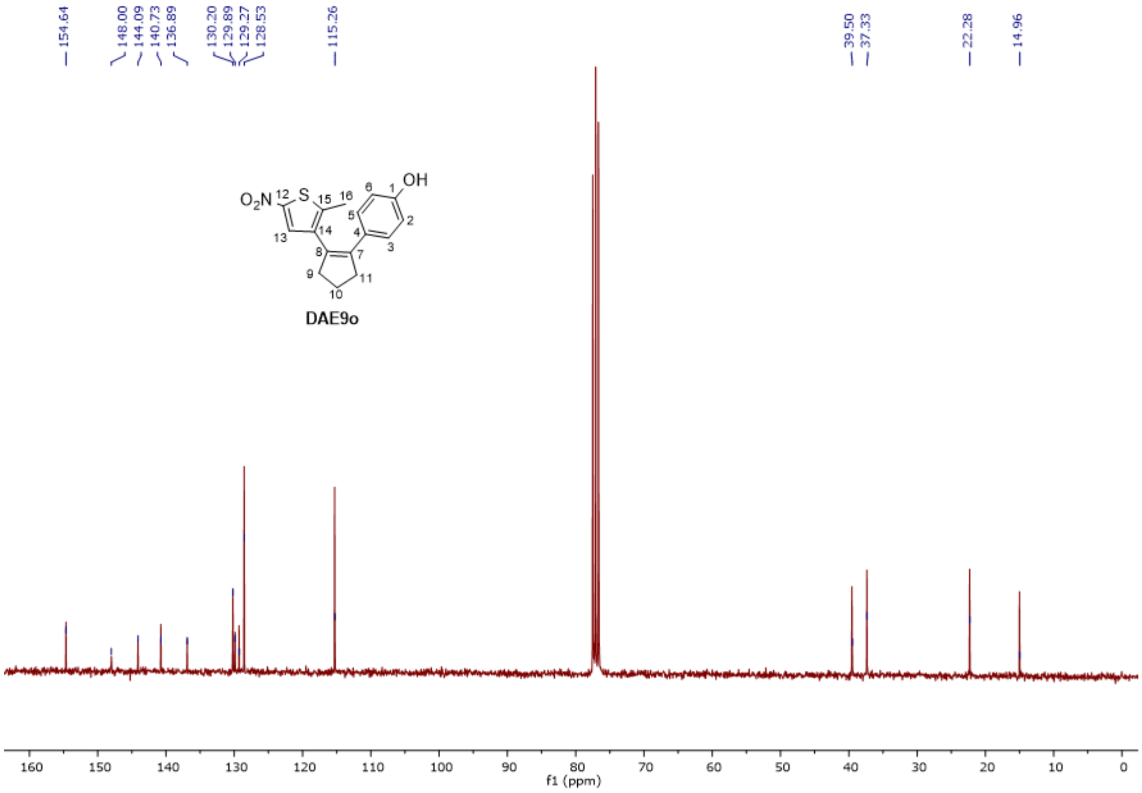

$^{13}C\{^{1}H\}$ NMR (75 MHz, $CDCl_3$)

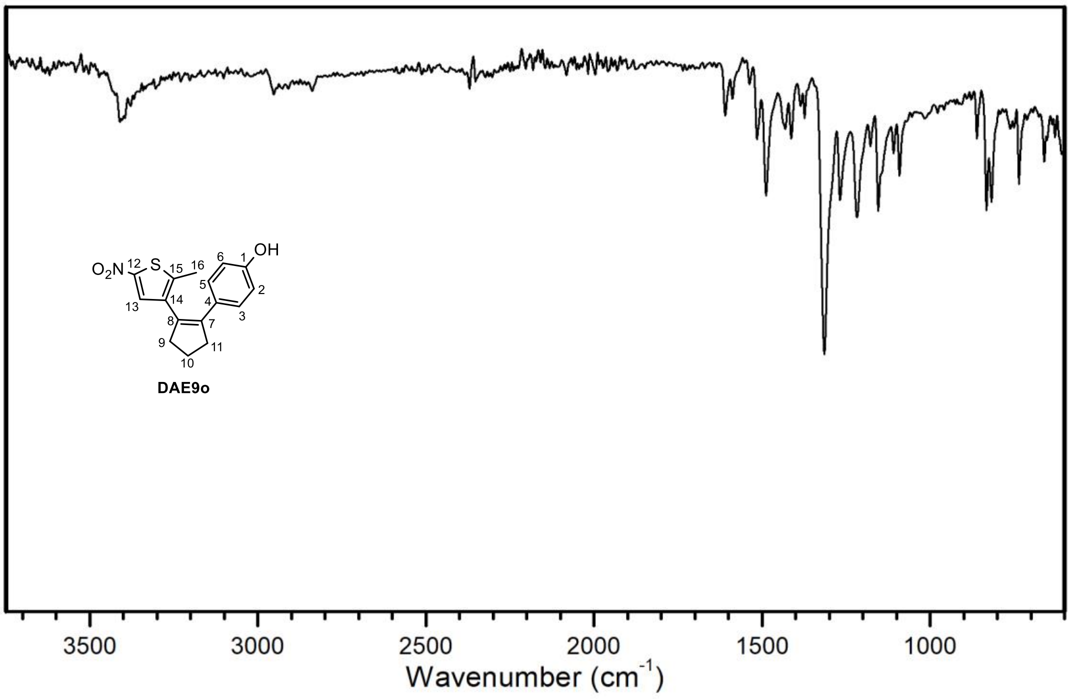

IR (ATR cm^{-1})

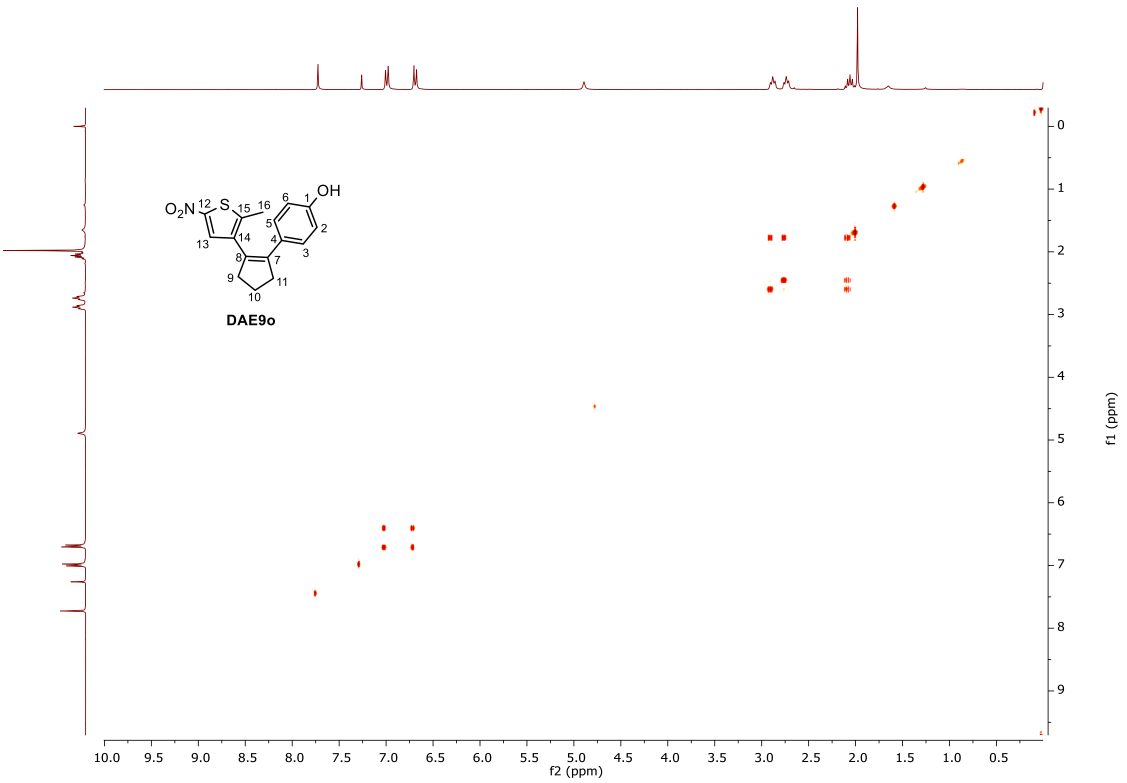

^{1}H-^{1}H COSY NMR (600 MHz, CDCl$_3$)

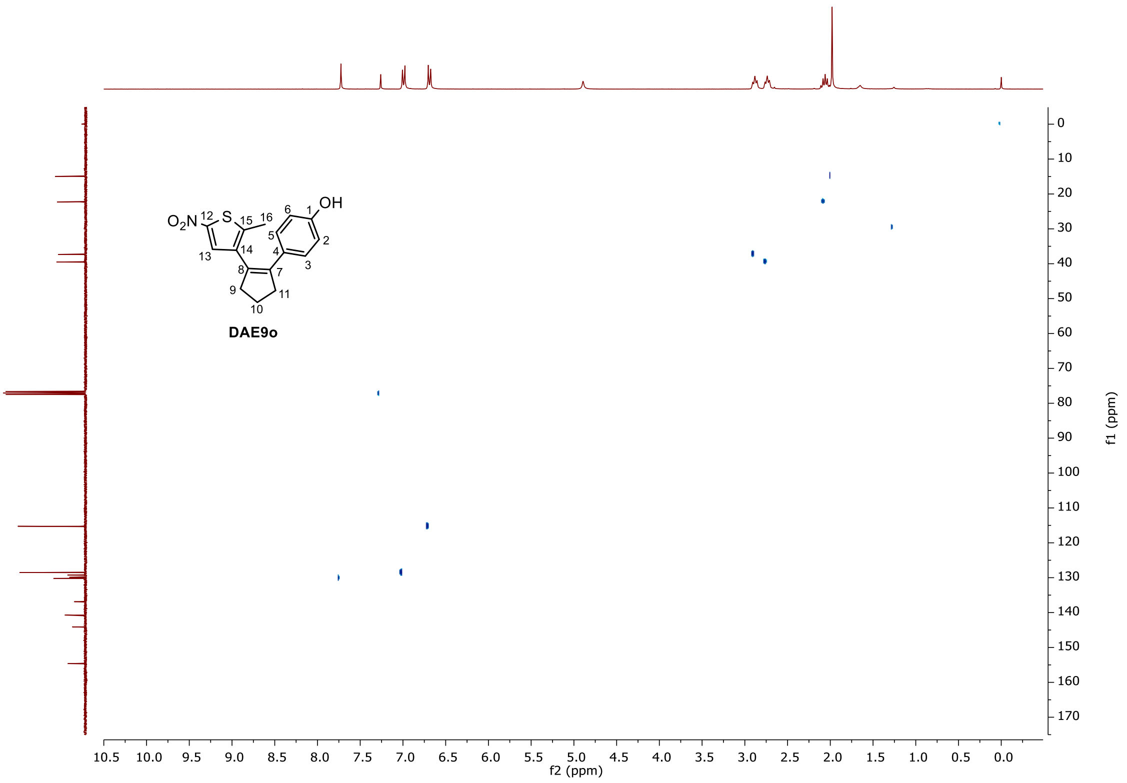

^{1}H-^{13}C HSQC NMR (600 MHz, CDCl$_3$)

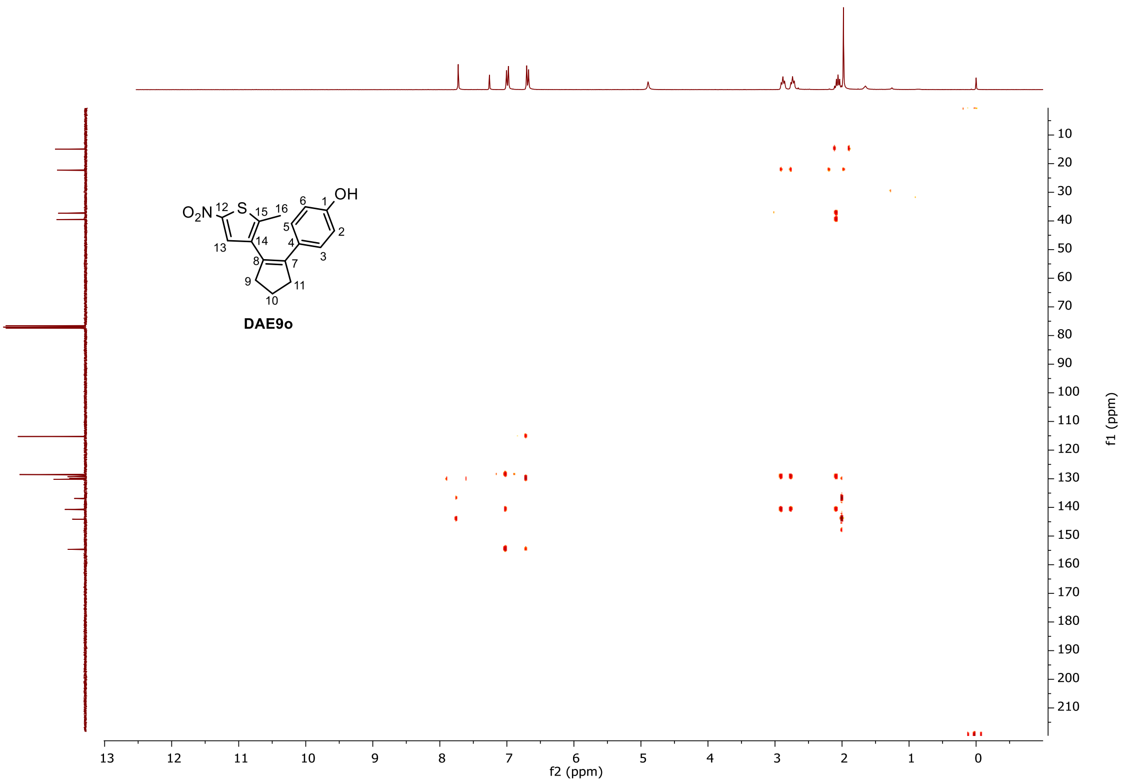

^1H-^{13}C HMBC NMR (600 MHz, $CDCl_3$)

6. References

(1) Hansch, C.; Leo, A.; Taft, R. W. A Survey of Hammett Substituent Constants and Resonance and Field Parameters. *Chem. Rev.* **1991**, *91* (2), 165– 195.

(2) Sevez, G.; Pozzo, J. L. Toward Multi-Addressable Molecular Systems: Efficient Synthesis and Photochromic Performance of Unsymmetrical Bisthienylethenes. *Dyes Pigm.* **2011**, *89* (3), 246–253.

(3) Kogami, M.; Watanabe, N. Convenient Method for the Preparation of the 2-Methyl Thiophen-3-yl Magnesium Bromide Lithium Chloride Complex and Its Application to the Synthesis of 3-Substituted 2-Methylthiophenes, *Synth. Commun.* **2012**, *43* (5), 681–688.

(4) Hermes, S.; Dassa, G.; Toso, G.; Bianco, A.; Bertarelli, C.; Zerbi, G. New Fast Synthesis Route for Symmetric and Asymmetric Phenyl-Substituted Photochromic Dithienylethenes Bearing Functional Groups such as Alcohols, Carboxylic Acids, or Amines. *Tetrahedron Lett.* **2009**, *50* (14), 1614–1617.

(5) Shrestha, T. B.; Troyer, D. L.; Bossmann, S. H. Strategies for Large-Scale Synthesis of Coelenterazine for in Vivo *Applications. Synthesis,* **2014**, *46* (5), 646–652.

(6) Lees, A. J. A Photochemical Procedure for Determining Reaction Quantum Efficiencies in Systems with Multicomponent Inner Filter Absorbances. *Anal. Chem.* **1996**, *68* (1), 226–229.

(7) Higashiguchi, K.; Matsuda, K.; Asano, Y.; Murakami, A.; Nakamura, S.; Irie, M. Photochromism of Dithienylethenes Containing Fluorinated Thiophene Rings. *Eur. J. Org. Chem.* **2005**, *2005* (1), 91–97.

(8) Zheng, C.; Pu, S.; Xu, J.; Luo, M.; Huang, D.; Shen, L. Synthesis and the Effect of Alkyl Chain Length on Optoelectronic Properties of Diarylethene Derivatives. *Tetrahedron* **2007**, *63* (25), 5437–5449.

(9) Irie, M.; Lifka, T.; Kobatake, S.; Kato, N. Photochromism of 1,2-Bis(2-methyl-5-phenyl-3-thienyl)perfluorocyclopentene in a Single-Crystalline Phase. *J. Am. Chem. Soc.* **2000**, *122* (20), 4871–4876.

(10) Espinosa, S.; Bosch, E.; Roses, M. Retention of Ionizable Compounds on HPLC. 5. pH Scales and the Retention of Acids and Bases with Acetonitrile−Water Mobile Phases. *Anal. Chem.* **2000**, *72* (21), 5193–5200.

(11) Hawai, S. H.; Gilat, S. L.; Lehn, J.-M. Photochemical pK_a-Modulation and Gated Photochromic Properties of a Novel Diarylethene Switch. *Eur. J. Org. Chem.* **1999**, *1999* (9), 2359-2366.

(12) Odo, Y.; Matsuda, K.; Irie, M. pK_a Switching Induced by the Change in the π-Conjugated System Based on Photochromism. *Chem. Eur. J.* **2006**, *12* (16), 4283 – 4288.

(13) Kobatake, S.; Terakawa, Y. Acid-Induced Photochromic System Switching of Diarylethene Derivatives between P- and T-Types. *Chem. Commun.* **2007**, *2007* (17), 1698-1700.

(14) Massaad, J.; Micheau, J.-C.; Coudret, C.; Sánchez, R.; Guirado, G.; Delbaere, S. Gated Photochromism and Acidity Photomodulation of a Diacid Dithienylethene Dye. *Chem. Eur. J.* **2012**, *18* (21), 6568 – 6575.

(15) Gurke, J.; Budzák, S.; Schmidt, B. M.; Jacquemin, D.; Hecht, S. Efficient Light-Induced pK_a Modulation Coupled to Base-Catalyzed Photochromism. *Angew. Chem. Int. Ed.* **2018**, *57* (17), 4797 –4801.

(16) Liu, D.; Sponza, A. D.; Yang, D.; Chiu, M. Modulating Polymer Dispersity with Light: Cationic Polymerization of Vinyl Ethers Using Photochromic Initiators. *Angew. Chem. Int. Ed.* **2019**, *58* (45), 16210 – 16216.

(17) Wilm, L. F. B.; Das, M.; Janssen-Müller, D.; Mück-Lichtenfeld, Glorius, F.; Dielmann, F. Photoswitchable Nitrogen Superbases: Using Light for Reversible Carbon Dioxide Capture. *Angew. Chem. Int. Ed.* **2022**, *61* (3), e202112344.

(18) Krishnan, C. G.; Kondo, M.; Yasuda, O.; Fan, D.; Nakamura, K.; Wakabayashi, Y.; Sasai, H.; Takizawa, S. Light-Controlled pK_a Value of Chiral Brønsted Acid Catalysts in Enantioselective aza-Friedel–Crafts Reaction. *Chem. Commun.* **2023**, *59* (66), 9956-9959.
