# Supplementary figures and images for: Amplified Light-Induced pKa Modulation with Diarylethene Photoswitches
Source: J Org Chem. 2024 Nov 30;89(24):17991–8002. doi: 10.1021/acs.joc.4c01606 (PMC11667731; doi:10.1021/acs.joc.4c01606)

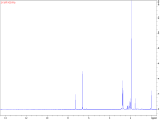

Supplement: Supplementary file 2 — jo4c01606_si_002.zip [file jo4c01606_si_002.zip › FID for publication part 1/Characterization zip part 1/1/1 HNMR/pdata/1/thumb.png]

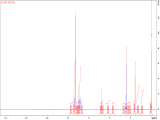

Supplement: Supplementary file 2 — jo4c01606_si_002.zip [file jo4c01606_si_002.zip › FID for publication part 1/Characterization zip part 1/11/11 HNMR/pdata/1/thumb.png]

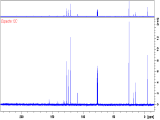

Supplement: Supplementary file 2 — jo4c01606_si_002.zip [file jo4c01606_si_002.zip › FID for publication part 1/Characterization zip part 1/12/12 CNMR/pdata/1/thumb.png]

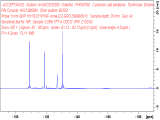

Supplement: Supplementary file 2 — jo4c01606_si_002.zip [file jo4c01606_si_002.zip › FID for publication part 1/Characterization zip part 1/14/14 FNMR/pdata/1/thumb.png]

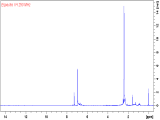

Supplement: Supplementary file 2 — jo4c01606_si_002.zip [file jo4c01606_si_002.zip › FID for publication part 1/Characterization zip part 1/14/14 HNMR/pdata/1/thumb.png]

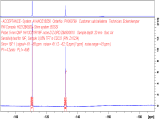

Supplement: Supplementary file 2 — jo4c01606_si_002.zip [file jo4c01606_si_002.zip › FID for publication part 1/Characterization zip part 1/15/15 FNMR/pdata/1/thumb.png]

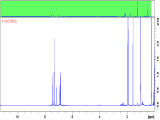

Supplement: Supplementary file 2 — jo4c01606_si_002.zip [file jo4c01606_si_002.zip › FID for publication part 1/Characterization zip part 1/15/15 HNMR/pdata/1/thumb.png]

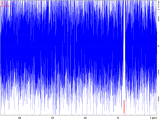

Supplement: Supplementary file 3 — jo4c01606_si_003.zip [file jo4c01606_si_003.zip › FID for publication part 2/Characterization zip part 2/DAE6o/DAE6o CNMR/pdata/1/thumb.png]

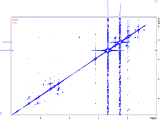

Supplement: Supplementary file 3 — jo4c01606_si_003.zip [file jo4c01606_si_003.zip › FID for publication part 2/Characterization zip part 2/DAE6o/DAE6o COSY/pdata/1/thumb.png]

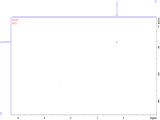

Supplement: Supplementary file 3 — jo4c01606_si_003.zip [file jo4c01606_si_003.zip › FID for publication part 2/Characterization zip part 2/DAE6o/DAE6o HMBC/pdata/1/thumb.png]

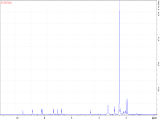

Supplement: Supplementary file 3 — jo4c01606_si_003.zip [file jo4c01606_si_003.zip › FID for publication part 2/Characterization zip part 2/DAE6o/DAE6o HNMR/pdata/1/thumb.png]

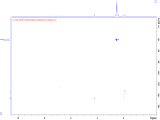

Supplement: Supplementary file 3 — jo4c01606_si_003.zip [file jo4c01606_si_003.zip › FID for publication part 2/Characterization zip part 2/DAE6o/DAE6o HSQC/pdata/1/thumb.png]

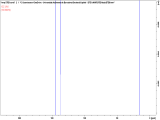

Supplement: Supplementary file 3 — jo4c01606_si_003.zip [file jo4c01606_si_003.zip › FID for publication part 2/Characterization zip part 2/DAE7o/DAE7o CNMR/pdata/1/thumb.png]

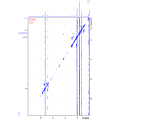

Supplement: Supplementary file 3 — jo4c01606_si_003.zip [file jo4c01606_si_003.zip › FID for publication part 2/Characterization zip part 2/DAE7o/DAE7o COSY/pdata/1/thumb.png]

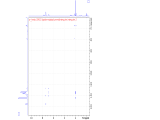

Supplement: Supplementary file 3 — jo4c01606_si_003.zip [file jo4c01606_si_003.zip › FID for publication part 2/Characterization zip part 2/DAE7o/DAE7o HMBC/pdata/1/thumb.png]
